# Machine Learning Enabled Prediction of Biologically Relevant Gene Expression Using CT‐Based Radiomic Features in Non‐Small Cell Lung Cancer

**DOI:** 10.1002/cam4.70509

**Published:** 2024-12-24

**Authors:** Shrey S. Sukhadia, Christopher Sadee, Olivier Gevaert, Shivashankar H. Nagaraj

**Affiliations:** ^1^ Centre for Genomics and Personalized Health and School of Biomedical Sciences Queensland University of Technology Brisbane Queensland Australia; ^2^ Department of Pathology and Laboratory Medicine Dartmouth‐Hitchcock Medical Center Lebanon New Hampshire USA; ^3^ Stanford Center for Biomedical Informatics Research, Department of Medicine and Biomedical Data Science Stanford University California USA; ^4^ Department of Biomedical Data Science Stanford University California USA

**Keywords:** gene expression and non‐small cell lung cancer, machine learning, radiogenomics, radiomics

## Abstract

**Background:**

Non‐small‐cell lung cancer (NSCLC) remains a global health challenge, driving morbidity and mortality. The emerging field of radiogenomics utilizes statistical methods to correlate radiographic tumor features with genomic characteristics from biopsy samples. Radiomic techniques automate the precise extraction of imaging features from tumor regions in radiographic scans, which are subjected to machine learning (ML) to predict genomic attributes.

**Methods:**

In a retrospective study of two NSCLC patient cohorts separated by 5 years, we performed a radiogenomic analysis of previously disseminated data from 2018 (*n* = 116) and newly acquired data from 2023 (*n* = 44) using RNA sequencing and lung CT images. Combining the data from two cohorts post binarization (of gene expression) or batch normalization (of radiomic features) in each cohort proved to be a better approach as compared to training the model on one cohort and validating on the other.

**Results:**

Our ML‐based radiogenomic modeling identified specific imaging features—wavelet, three‐dimensional local binary patterns, and logarithmic sigma of gray‐level variance—as predictive indicators for high (1) vs. low (0) gene expression of pivotal NSCLC‐related genes: *SLC35C1*, *BCL2L1*, and *MAPK1*. These genes have recognized implications in a variety of biological pathways and mechanisms of drug resistance pertinent to NSCLC.

**Conclusion:**

The successful integration of heterogeneous radiogenomic datasets underscores the potential of imaging biomarkers in uncovering NSCLC biological processes through gene expression profiles.

## Graphical abstract

In the present study, we used two heterogeneous NSCLC cohorts to make two substantial advances in the field of radiogenomics: (1) we successfully combined two temporally distinct cohorts, thereby addressing a long‐standing problem in radiogenomics, and (2) we show that radiomic features extracted from tumor regions of interest from SOC images can be used as surrogate biomarkers for the individual expression of several genes known to play key roles in NSCLC and other human malignancies. We believe that our study makes a significant contribution to the literature because it represents substantial advances in the field of radiogenomics and provides a blueprint for homogenizing temporally distinct patient cohorts and using predictions of individual gene expression as surrogate biomarkers in clinical oncology workflows.

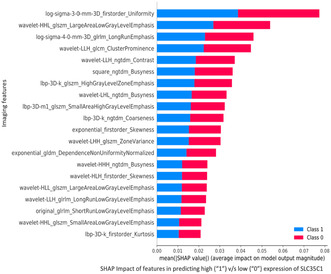

## 1 Introduction

Non‐small cell lung cancer (NSCLC) is the most common type of lung cancer and remains a leading cause of cancer‐related deaths worldwide [1, 2]. The prognosis for NSCLC patients remains poor, with a 5‐year survival rate of approximately 20% [3]. While recent advances in targeted therapies and immunotherapies have shown promise in improving outcomes for NSCLC patients, identifying the most effective treatment for each patient remains a challenge [4].

Radiogenomics is an emerging field that combines standard‐of‐care (SOC) radiological imaging with tumor genetics to identify genetic mutations and molecular pathways associated with imaging features [5]. The field of radiogenomics has the potential to improve patient care by providing non‐invasive predictions of gene expression profiles and identifying potential therapeutic targets for personalized treatment [6]. In recent years, radiogenomics has gained interest in NSCLC owing to its potential for identifying associations between imaging features and genetic mutations or molecular pathways that drive tumor growth and metastases [6, 7, 8, 9, 10, 11]. Several studies have demonstrated the potential of radiogenomics to predict gene expression profiles in NSCLC, suggesting that radiogenomics may provide valuable insights into the biological mechanisms of NSCLC to identify potential therapeutic targets for personalized treatment [6, 11]. However, the majority of these studies cluster genes co‐expressed in biological pathways prior to performing their correlation and modeling with imaging features [6, 11], which could potentially suppress the associations that are likely to exist between imaging features and the expression of individual genes. The predicted genes could be analyzed for their connections with each other in the biological pathways that are crucial in NSCLC. Thus, although the association between computed tomography (CT)‐based imaging features and expression profiles of individual genes in NSCLC could facilitate the monitoring of both individual and grouped genomic markers linked to tumor cell progression and metastasis, this has been poorly investigated in NSCLC.

Several statistical and Machine learning (ML) techniques have been previously employed to associate and predict genetic mutations and the expression of several genes using radiomic features from PET and CT scans in NSCLC, respectively [8, 10]. However, best practices for integrating diverse ML and statistical techniques to harmonize radiogenomic datasets with considerable time gaps between distinct cohorts or between centers have not been established. Furthermore, the lack of adequate data for training, testing, and validation of radiogenomic models hampers the clinical utility of these technologies [7]. These limitations are also an obstacle to building robust ML models based on diverse radiogenomic datasets, thereby preventing adequate validation or their translation into clinical practice. Moreover, available datasets are outdated and should be augmented with current data to account for advancements in imaging and genomic sequencing technologies [7], for which batch‐effect normalization would be required to arrive at robust and reproducible radiogenomic associations [12]. Lastly, establishing the predictive power of imaging features to serve as surrogate biomarkers of individual gene expression in radiogenomics is also a known limitation [7].

To address the aforementioned limitations, we sought to investigate the association between radiologic features extracted from tumor regions of interest (ROIs) on CT images and gene expression profiles obtained from the respective tumor tissue biopsies in NSCLC patients by combining the two distinct NSCLC cohorts from the same institution separated temporally by 5 years: (1) a publicly available NSCLC radiogenomic dataset (hosted at The Cancer Imaging Archive [TCIA]) from 2018 [14, 15] and (2) a newly generated NSCLC dataset from 2023. To do this, we implemented and tested both regression‐ and classification‐based ML approaches, to evaluate whether radiomic features extracted from lung computed tomography (CT) scans accurately predicted gene expression using RNA‐seq data from biopsied lung tumors. Our study proposes best practices for combining the two temporally distinct radiogenomic datasets, representing a considerable advancement in the integration of radiomic and genomic technologies to overcome known limitations of building and testing ML models using combined radiogenomic datasets [7]. Furthermore, we demonstrate the ability of specific imaging features to predict the individual expression (high vs. low) of several genes known to elevated or co‐expressed in crucial biological pathways involved in NSCLC.

## 2 Materials and Methods

### 2.1 Generation of Radiomic Data

The CT scans from an NSCLC cohort (*n* = 116) and their corresponding segmentation labels, illustrating tumor regions of interest (ROIs), were obtained from The Cancer Imaging Archive (TCIA) portal [15]. The CT scans of a second cohort (*n* = 44) were processed using Dune‐AI [16] to generate tumor segmentation labels, which were then carefully reviewed and refined for boundary accuracy using ITK‐SNAP software [17] (Figure S1). Radiomic features were subsequently extracted from these segmentation labels using Pyradiomics software (v3.0.1) [18]. Radiomic features underwent analysis using the correlation module in ImaGene [19]. Pearson‐based correlations were computed across various classes of radiomic features, including shape, size, gradient, wavelength, and local binary pattern‐3D. Subsequently, a hierarchical clustering based on the Euclidean distance method was employed to organize and visualize the relationships between these features.

### 2.2 Generation of Gene Expression Data

The RNA‐seq data, measured as fragments per kilobase per million reads (FPKM), that were available for the respective tissue‐biopsies for the old cohort, were downloaded [14]. Genes with FPKM value not reported in one or more than one sample were eliminated from the dataset. For the new cohort, the total Ribonucleic acid (RNA) was isolated from FFPE tissues using Promega Maxwell RSC RNA FFPE Kit (cat# AS1440). The quality of the total RNA was evaluated by generating the DV200 score using Tapestation 4200 (Agilent Technologies), which assessed the percentage of fragment lengths greater than 200 nt, and the quantification of total RNA was performed by Qubit (Invitrogen). Each sample's quality was evaluated and the samples were passed based on the validated manufacturer's quality requirements of DV200 > 20% and quantity requirements of > 250 pg. in 15 μL solution for compatibility of library preparation using the Takara SMARTer Stranded Total RNA‐Seq Kit v2—Pico Input Mammalian kit which uses random priming and does not require polyA tails. During the library preparation stage, 10 ng total RNA input was used following manufacturer's instructions to synthesize cDNA fragments using random primers. SMART technology is used to preserve the strand orientation information. Adapters for Illumina sequencing (with specific barcodes) was added through PCR using only a limited number of cycles (5 cycles). The ribosomal cDNA fragments were then cleaved using ZapR v2 enzyme in the presence of rRNA specific probes. The library fragments from non‐rRNA molecules were then enriched by a second round of amplification. The final library quality was estimated using Agilent Tapestation 4200 for single peak ranging from 300 to 350 bp and quantification was done using Qubit Flex (Invitrogen) for > 4 nM. Prior to sequencing, libraries were diluted to four nmoles and pooled. Pooled libraries were sequenced on NovaSeq 6000 (Illumina) following manufacturer's instructions using 300 cycle kit, paired end 100 basepair reads. Raw reads were generated from run base‐call (BCL) files using the bcl2fastq tool version v2.20.0.422. Quality of the reads was assessed using in‐house fastqc scripts. Fastq file QC was evaluated against Illumina's manufacturer's guideline that states Q30 > 85% for passing metrics. Majority of samples had Q30 > 90%. The resulting Fastq files were processed for Illumina‐adapter trimming using TrimGalore software (version 0.6.6). The adapter‐trimmed FASTQs were aligned to human reference genome (version hg19) using STAR Aligner (version 2.6.0) to yield Binary Alignment Map (BAM) files. The percent reads aligned was found to be 97% on average across all samples. Gene‐expression (FPKM) values were called from BAMs using Cufflinks (version 2.2.1).

### 2.3 Correlation Between Radiomic Features and Gene Expression Data

The radiomic features and gene‐expression data were correlated using Pearson's correlation method and were then filtered using an absolute correlation coefficient threshold, |*r*| > = 0.5 [16] with a Bonferroni‐Hochberg corrected *p* value of less than 0.05, followed by hierarchical clustering based on Euclidian distance to obtain significantly correlated radiogenomic feature‐clusters. Pearson‐based correlation technique is one of the most common methods used in previous radiogenomic correlational studies [10, 16]. Hierarchical clustering technique is widely adopted in radiogenomic studies as well [12, 13]. We combined these two techniques to increase robustness of associations between radiomic and gene‐expression features.

Also, as the features are derived from two different modalities (radiology and genomics), they could potentially have their signals biased due to the way they are measured or extracted, that is, FPKMs for gene‐expression and handicraft radiomic features (based on pre‐defined statistical formulas) extracted from Pyradiomics. Therefore, it is important to focus on the higher rather than the lower end of the correlation co‐efficient between these features which is tightly regulated by the FDR adjusted *p* values to limit the correlations based on their significance. This has been a common strategy used in several radiogenomic studies previously [2, 3, 4, 6]. Setting the absolute correlation co‐efficient threshold to greater than or equal to ‘0.5’ and FDR adjusted *p* value to less than 0.05 ensured that we considered the most significant radiogenomic correlations for our study yielding robust outcomes downstream.

### 2.4 Building a Multitask Elastic Net Model

We built the Multitask Elastic‐Net (MTEN) model using ImaGene software [19], using the significantly correlated radiomic and gene expression features in the old cohort. The cohort was split into a 80:20 (training: testing) ratio. Both, radiomic and gene‐expression features were normalized using Standard Scaler technique. Additionally, we conducted a three‐fold cross validation of our training set to train through the default model hyperparameters as referenced in the scikit‐learn library (https://scikit‐learn.org/stable/modules/generated/sklearn.linear_model.MultiTaskElasticNet.html) and ImaGene [19]. The area under the receiver operating curve (AUROC or AUC) and the co‐efficient of determination (*R* ^2^) were measured using the testing set. The validation of the model was performed on the new cohort.

### 2.5 Targeting Genes Known to Be Expressed in NSCLC

Next, we targeted a set of 67 genes that were identified to be either high or low expressed in NSCLC based on the literature [17, 21]. Only 33 out of 67 genes were found to be present on our list of genes in the gene‐expression dataset. The expression of each of the 33 genes in each individual cohort were binarized to yield either low (‘0’) or high (‘1’) expression values depending on whether they fell either below or above the median‐expression of the respective gene in the respective cohort. The binarized gene‐expression from both the cohorts were merged into a single robust cohort of 160 samples in total.

Further, we built a multiclass Random Forest (RF) classifier, using 85:15 as training: testing ratio. The training was conducted using the following hyperparameters for a grid‐search: (a) max‐depth = [4, 5, 6, 7, 8, 9, 10, 11, 12, 14, 15, 16, 17, 18, 19, 25, 28, 35, 40, 51, 55] and (b) six‐fold cross validation. The model was tested using the testing set. The SHapley Additive exPlanations (SHAP) scores depicting the contribution of radiomic features toward the classification of gene‐expression into high and low expression was measured using a python‐based SHAP tool [20, 22]. The top scoring radiomic features were reviewed.

Furthermore, a biological pathway analysis was conducted for the genes best predicted by RF classifier using STRING‐DB software/web‐portal [23], with the aim of identifying new neighboring genes for which the prediction could be established using radiomic features using the same RF classification technique. To be more specific, we used the STRING version 12.0 web‐portal: “https://string‐db.org/cgi/input?sessionId=bnk9Mx2hKphn.”

## 3 Results

### 3.1 Generation of Radiomic Features

**FIGURE 1:**
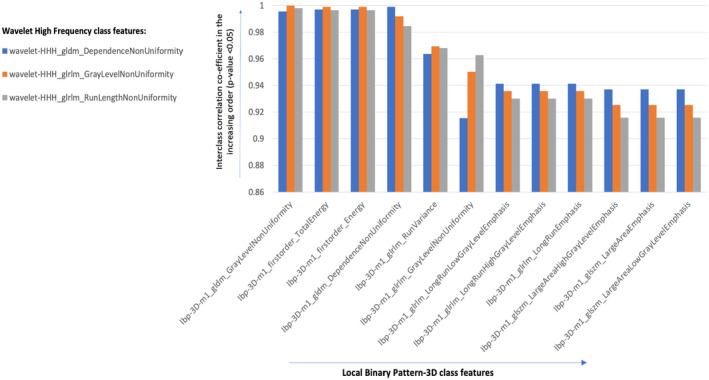
High interclass correlations (|*r*| > 0.9) between radiomic features belonging to local binary pattern (LBP) 3D and wavelet high frequency (HHH) classes at False discovery rate adjusted *p* value < 0.05.

Feature‐extraction performed using segmentation labels on CT‐scans using Pyradiomics [18] yielded 2105 radiomic features belonging to the following classes: shape, size, gradient, wavelet, and local‐binary pattern‐3D. The feature classes that had Pearson's correlation co‐efficient threshold |*r*| > 0.9 (*Bonferroni‐Hochberg corrected p* < 0.05) got clustered hierarchically using Euclidian distance method yielding 583 highly redundant interclass radiomic feature‐clusters that were filtered out. For example, Figure 1 depicts the high interclass correlation between features from local binary pattern‐3D (LBP‐3D) and wavelet higher frequency (HHH) classes that were filtered out. Consequently, a total of 1522 radiomic features were retained post filtration (Table S3). These contain feature from variety of classes such as wavelet, shape, size, and gradient class.

### 3.2 Analysis of Gene Expression Data

RNA‐seq based gene expression (FPKM) data for the first cohort were downloaded from a previously published lung radiogenomic study [14]. This data were reviewed, and the genes having FPKM values annotated “not‐available” (Nas) were eliminated, thereby retaining a total of 5160 genes bearing quantifiable (non‐NA) FPKM values. For the second cohort, the custom bioinformatics pipeline that matched closely with the previous study [14] generated FPKMs for all genes in human genome (hg19 version) using FASTQs from the RNA‐seq performed on the respective FFPE tissue slides. Only the genes with the names matching those from the gene‐list of the older cohort (i.e., 5160 genes) were retained to achieve consistency in gene‐set between the two cohorts.

### 3.3 Correlation Between Radiomic Features and Gene Expression Data

**FIGURE 2:**
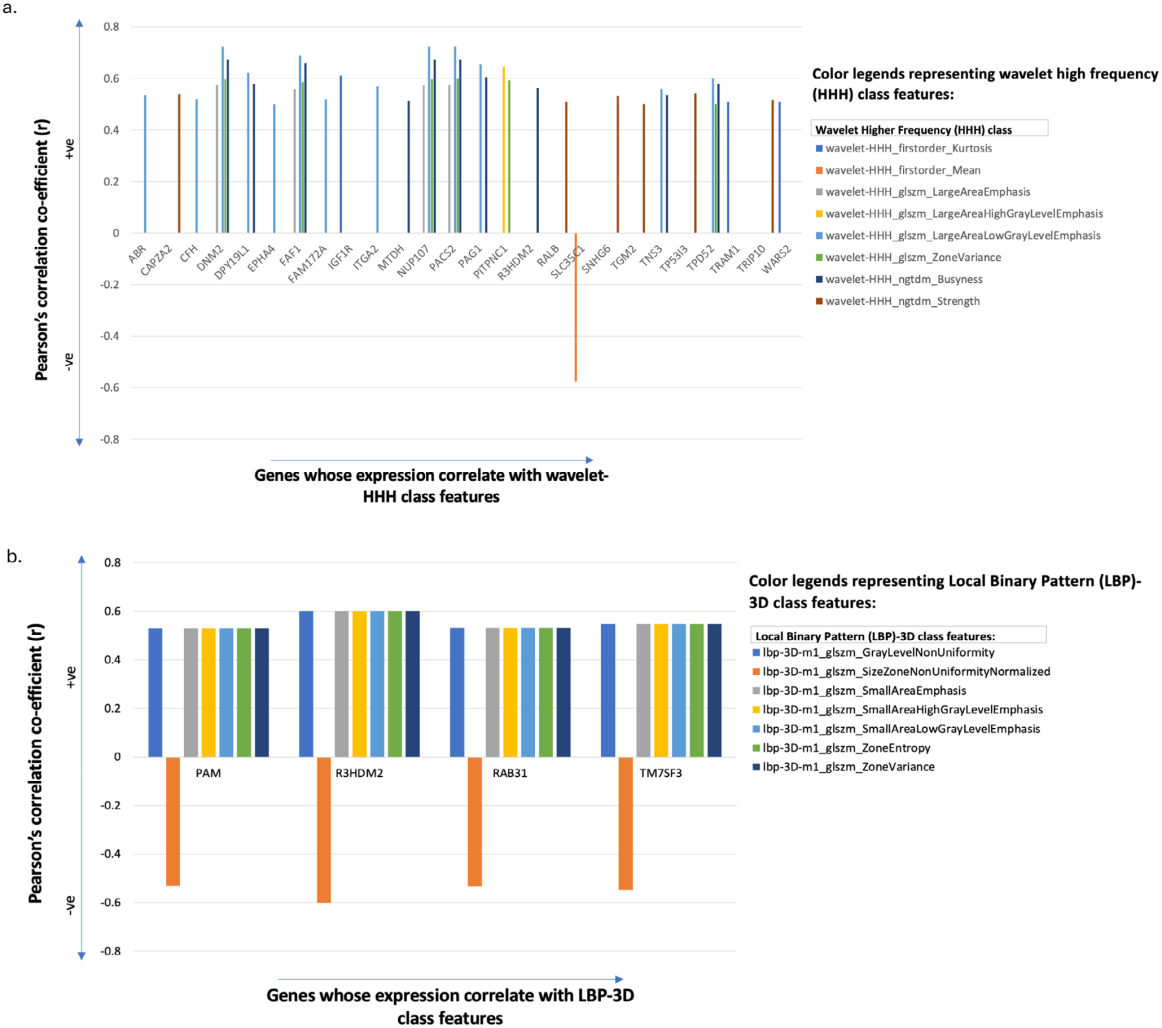
Wavelet high frequency class (a) and local binary pattern (LBP) 3D class (b) feature‐clusters correlating with the expression of multiple genes at Pearson's correlation co‐efficient threshold (|*r*| > 0.5) and FDR adjusted *p* value < 0.05. The correlation co‐efficient (*r*) and gene‐expression are plotted on *y* and *x* axes, respectively.

Next, a hierarchical clustering performed on significantly correlated radiomic features and gene‐expression data (|*r*| > 0.5, FDR adjusted *p* value < 0.05) revealed clusters of radiomic features correlating with the gene expression. For example, Figure 2a,b depicts wavelet higher frequency (HHH) and local binary pattern (LBP) 3D feature‐clusters correlating with expression of multiple genes, respectively. As a result, a total of 211 radiomic features (Table S2) were found to be correlated with the expression of 137 genes (Table S1).

### 3.4 Building a Multitask Elastic Net Model

We built a Multitask Elastic‐Net (MTEN) model using the significantly correlated radiomic and gene expression features in the training set (*n* = 93) from the first cohort. While we found a high AUC (> 0.8) in predicting the individual expression of SLC35C1 when testing the model with the testing set of the first cohort, validating with the second cohort resulted in low AUC values (AUC < 0.5). Thus, we investigated the data further and noted that the validation of the model may have failed owing to the skewness in the distribution of the expression of *SLC35C1* between the two cohorts (i.e., old, and new). This was rectified by binarizing the expression of *SLC35C1* as ‘0’ (low) or ‘1’ (high) expression depending on whether the expression fell below or above the median of *SLC35C1* expression in the samples in individual cohorts. The binarized expression of *SLC35C1* from both the cohorts were merged into a single cohort of 160 samples. Likewise, the radiomic features from each cohort were batch‐normalized using a “Standard Scaler” normalization technique and merged into a single cohort of 160 samples. The binarized gene‐expression warranted the use of a classification‐based model next, therefore, we chose to build a Random Forest Classifier on the merged cohort.

**FIGURE 3:**
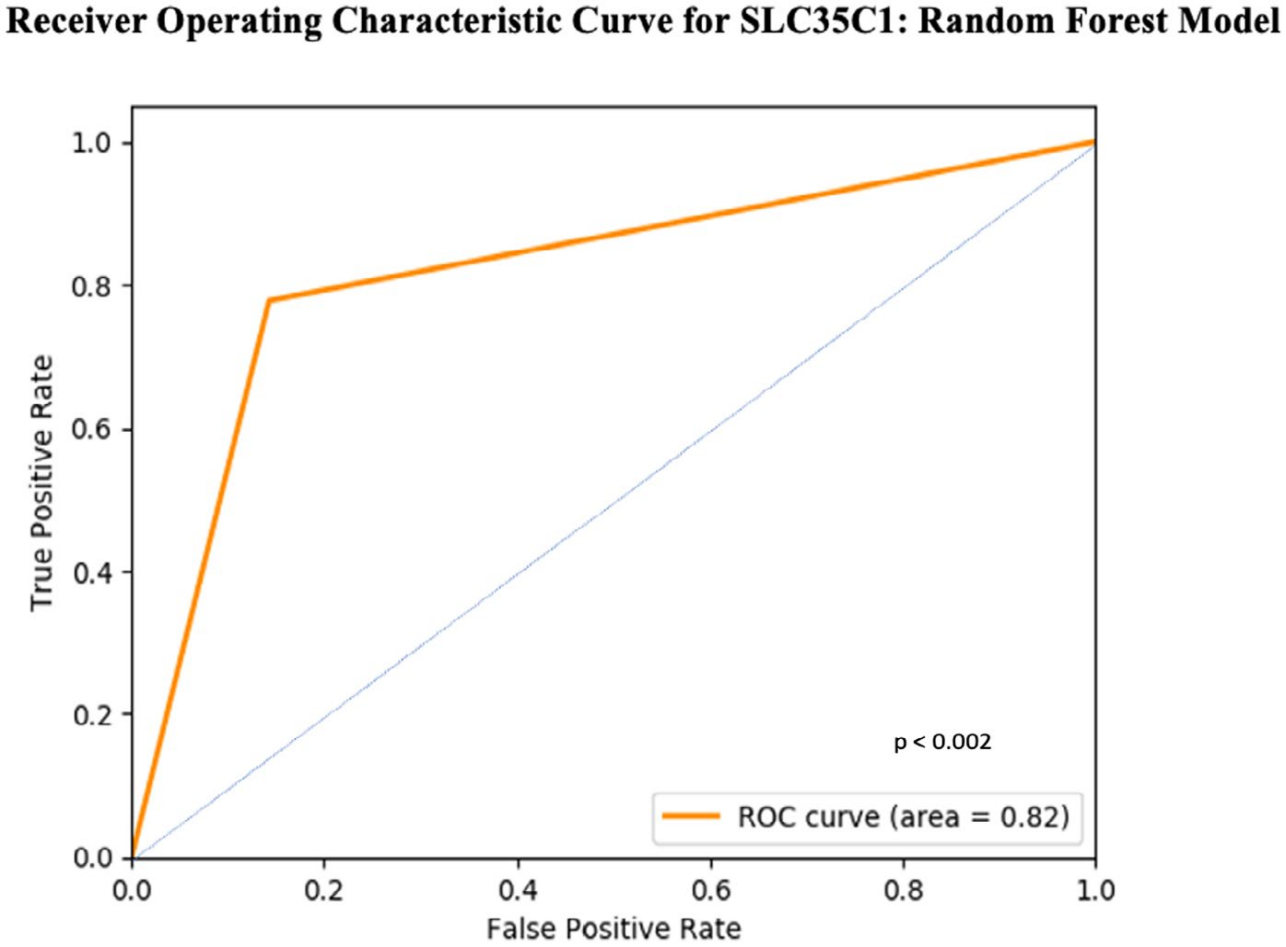
Receiver operating curve indicating high true positives and low false positives for the classification of *SLC35C1* expression (i.e., predicted class).

**FIGURE 4:**
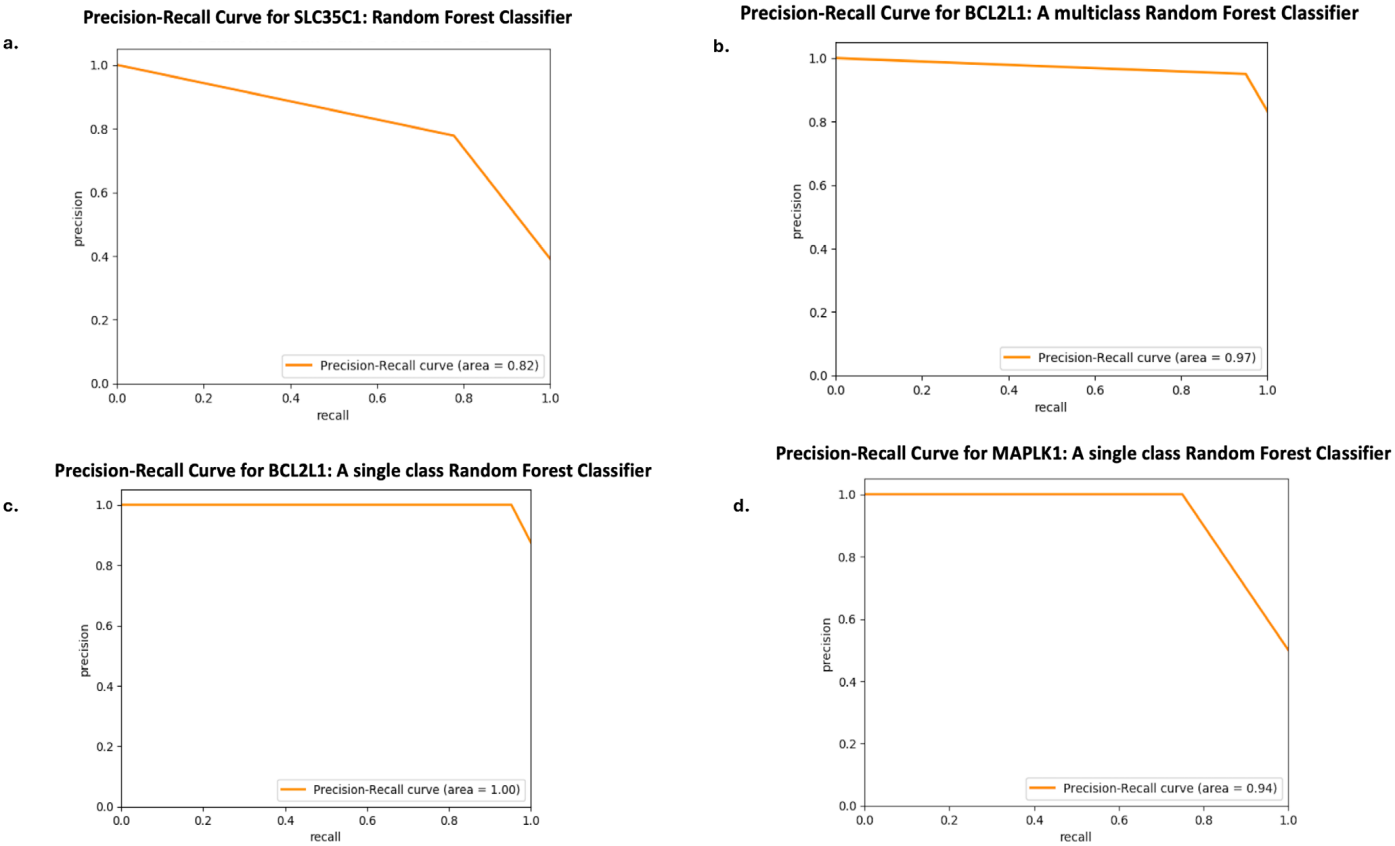
Precision‐Recall (PR) curve for the prediction of all three genes: SLC35C1, BCL2L1, and MAPK1 as displayed in (a–d), respectively, using their respective classifiers.

We built a Random Forest (RF) classifier by splitting the merged cohort into an 80:20 (training: testing) ratio. The classifier was trained using six‐fold cross validation with hyperparameters. Testing the model using the testing set (*n* = 31) classified the expression of *SLC35C1* into high and low levels of expression at AUC (and AUC_PR) > 0.8 and *R* ^2^ > 0.25 (*p* < 0.002) (Figures 3 and 4a).

**FIGURE 5:**
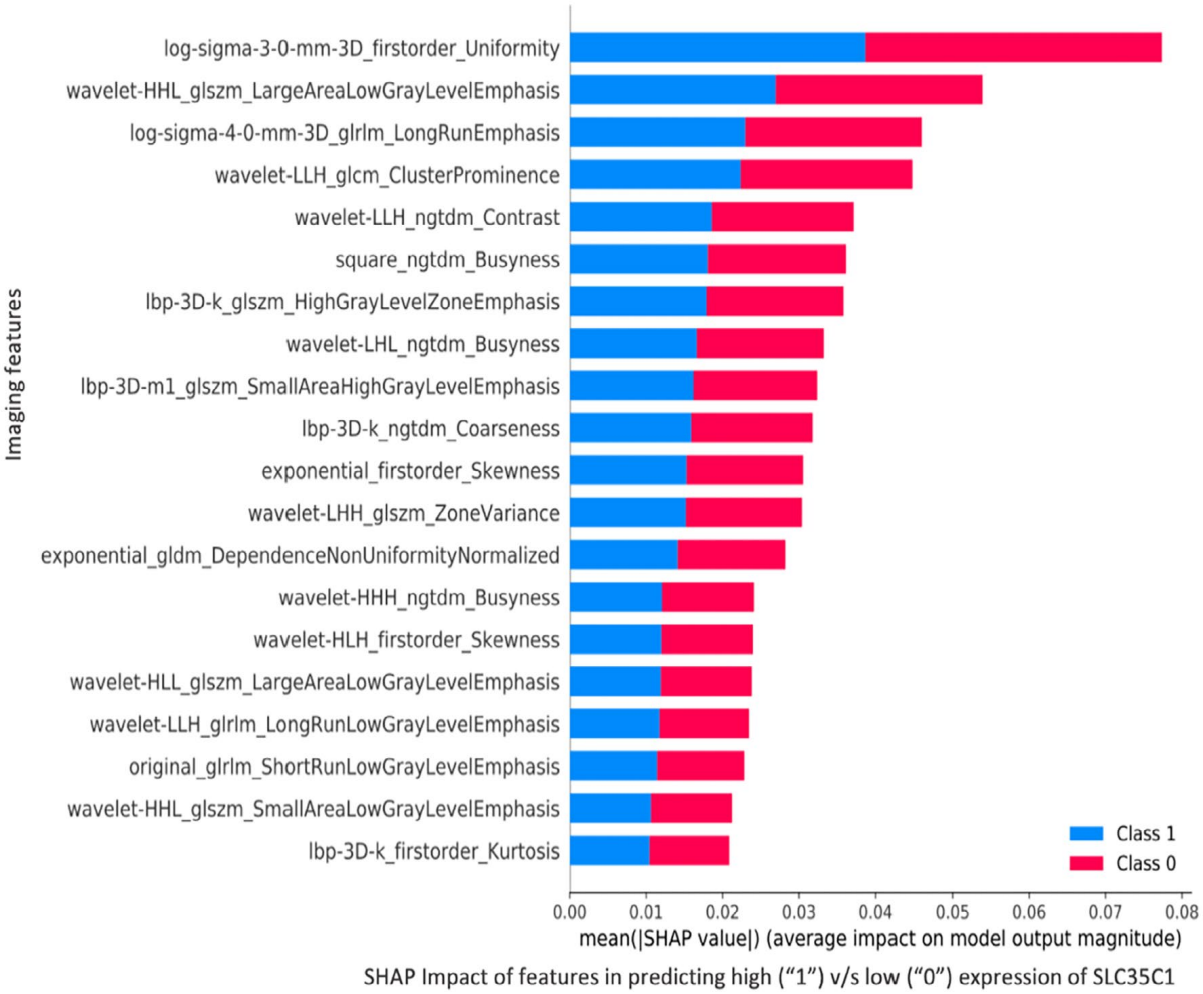
Measuring impact of radiomic (or imaging) features in predicting high (1) versus low (0) expression for *SLC35C1*. The top performing radiomic features belonged to the following classes of radiomic features: Wavelet frequency, 3DLBP, and 3D log sigma of first‐order and gray‐level variance.

SHAP‐based scores showed the top 20 radiomic features that contributed the most to the classification of *SLC35C1* into high and low expression (Figure 5). These impactful radiomic features mainly belonged to the following three radiomic classes: Wavelet frequency, LBP‐3D, and 3D log sigma of first‐order and gray‐level variance (Figure 5).

### 3.5 Targeting Genes Known to Be Expressed in NSCLC

Next, we gathered a targeted set of 67 genes that exhibited either elevated or decreased expression in NSCLC as presented in the literature [17, 21]. Only 33 out of 67 genes were found to be present on our original list of 5160 genes. The expression of those 33 genes had to be binarized in the old and new cohorts individually and subsequently combined into a merged cohort before we could build a multi‐class (multi‐gene) Random Forest classifier for their prediction.

**FIGURE 6:**
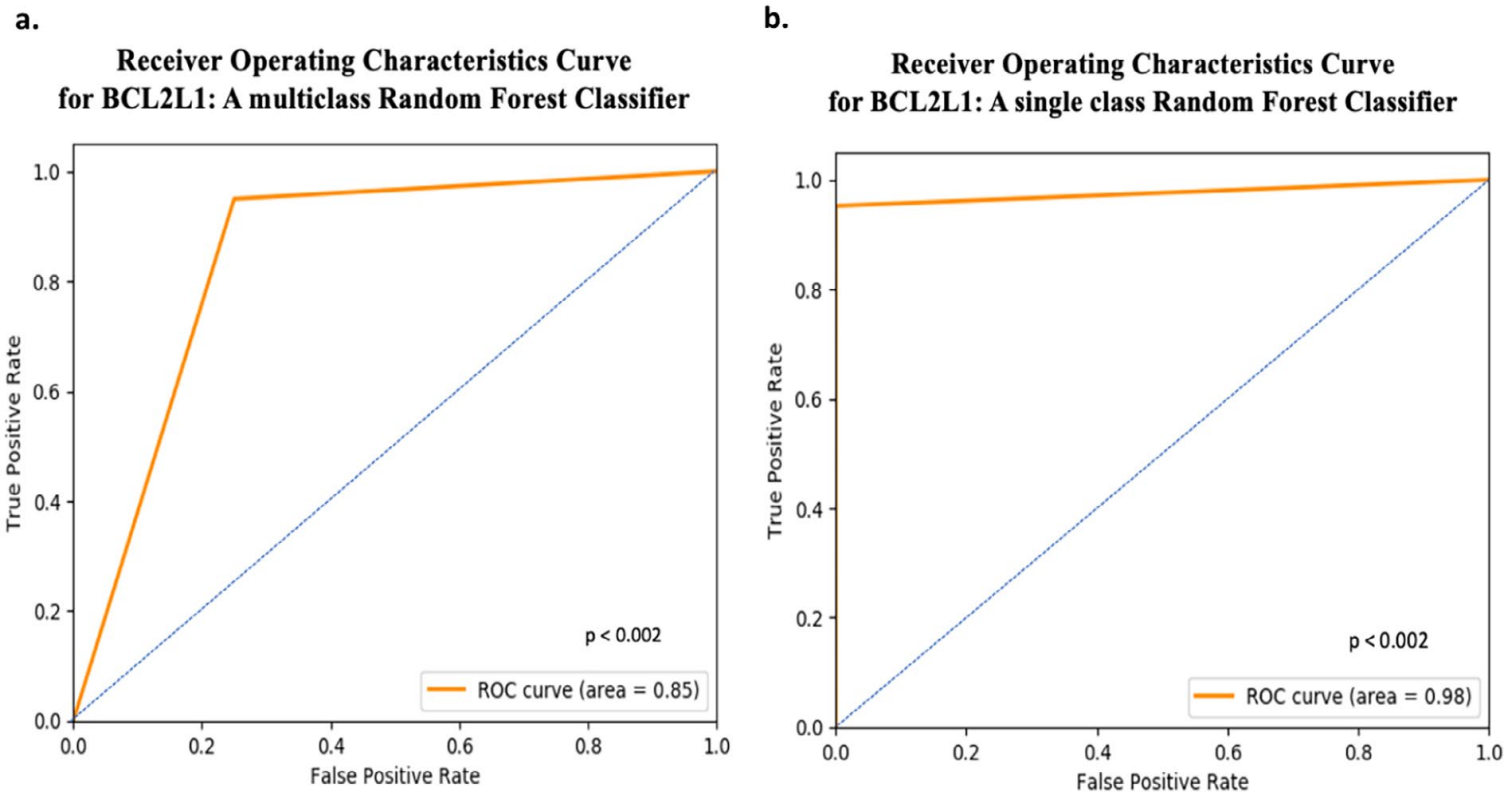
Receiver operator curves (ROC) for classification of *BCL2L1* expression (into high and low expression) using both multi‐ and single class Random Forest classifier: (a) ROC indicating high true positives and low false positives for the classification of *BCL2L1* expression using a multitask classifier, and (b) ROC indicating high true positives and low false positives for the classification of *BCL2L1* expression using a single‐task classifier.

The merged cohort was split 85:15 (training: testing) ratio and a multi‐class RF classifier was built using a six‐fold cross validation with hyperparameters on the training set. Testing the model using the testing set predicted the gene *BCL2L1* (a BCL‐2 family member) at an AUC of 0.85, AUC_PR of 0.97 and *R* ^2^ of 0.4, at *p* < 0.002 (Figures 4b and 6a). Next, we built a single class (*BCL2L1* gene‐label only) classifier using the same training set and tested with the same testing set, which yielded a test‐AUC of 0.95, test‐AUC_PR of 1.0 and test‐*R* ^2^ to 0.62, at *p* < 0.002 (Figures 4c and 6b), marking a significant increase in performance compared to the multi‐class RF classifier.

**FIGURE 7:**
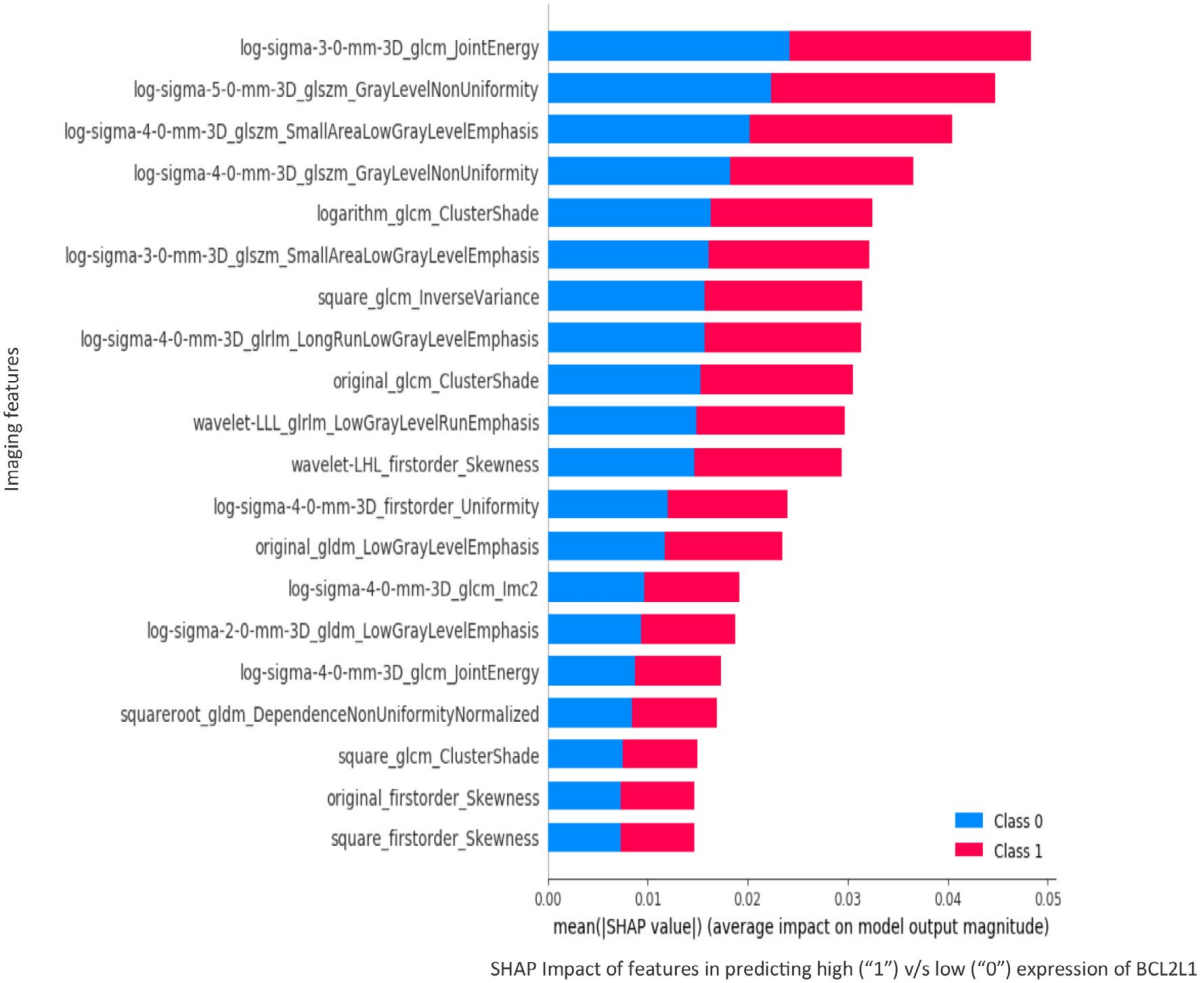
Measuring the impact of imaging features in predicting high (1) versus low (0) class of expression for *BCL2L1*, using SHapley Additive exPlanations (SHAP) scores.

The radiomic features that exerted the most impact on the classification of high and low expression of *BCL2L1* belonged mainly to the following feature classes: wavelet frequency and 3D log sigma first‐order and gray‐level emphasis (Figure 7).

Further, the exploration of biological pathways to identify gene neighbors for *BCL2L1* was conducted using the web‐version of STRING database (STRING‐DB) [23], where a k‐means clustering followed by a co‐expression analysis yielded nine closest gene‐neighbors: *CDKN1A*, *FOXO3*, *MAPK1*, *BAX*, *TP53*, *CYCS*, *GADD45A*, *CDK4*, and *CASP*, which are known to be co‐expressed in several pathways in cancer (Figure S2).

**FIGURE 8:**
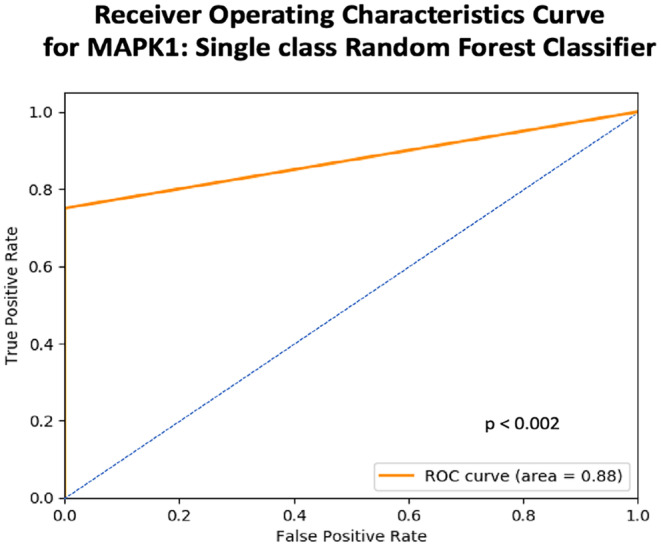
Receiver operating curve (ROC) indicating high true positives and low false positives for the single‐task classification of *MAPK1* expression using a grid‐search approach.

**FIGURE 9:**
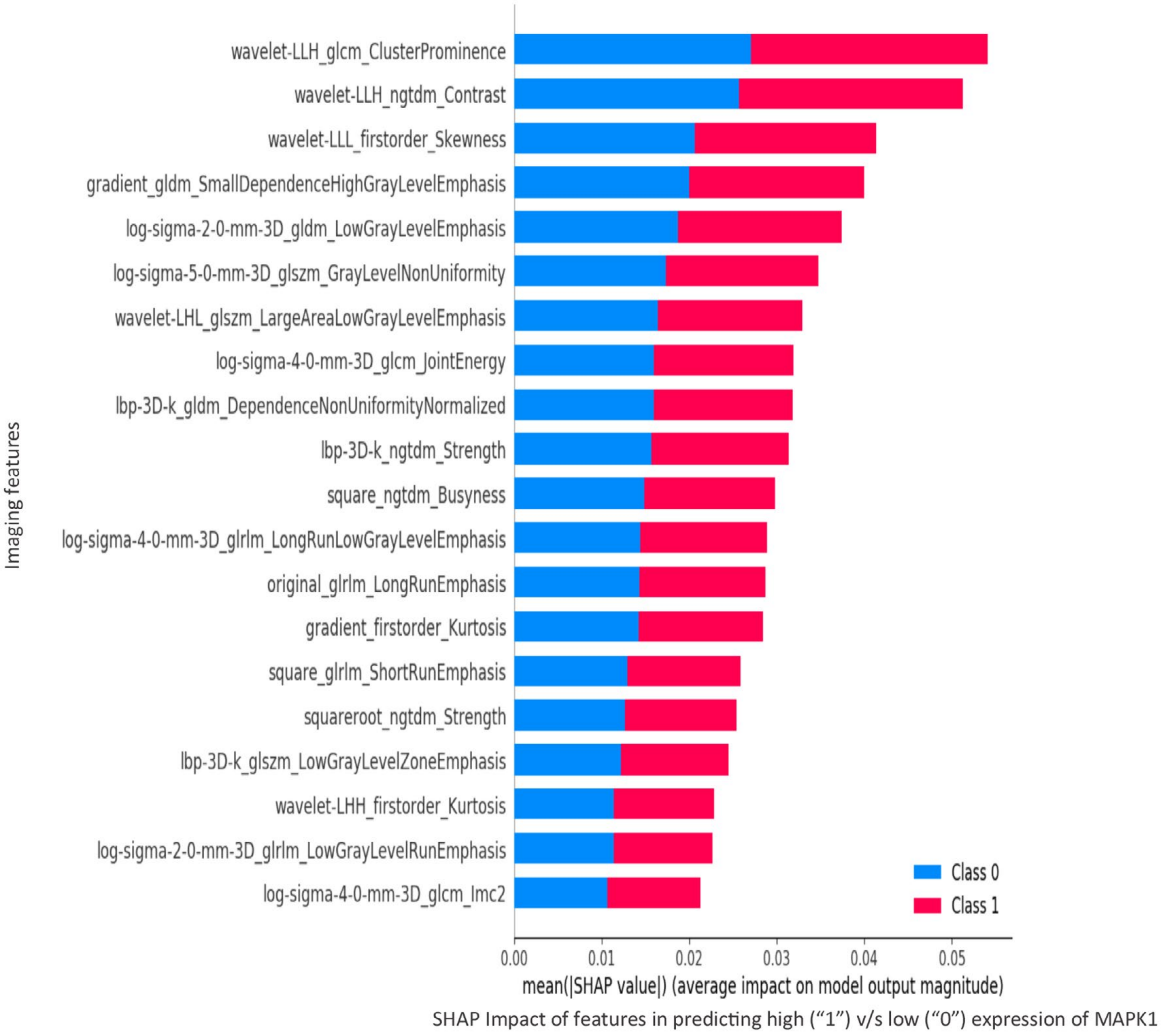
Measuring the impact of imaging features for prediction of high (1) versus low (0) expression of *MAPK1* gene.

A single‐task RF classifier was built for the prediction of each of the gene‐neighbors using a 95:5 (training: testing) split of the merged cohort. Training of the model was conducted using 18‐fold cross‐validation of the training set to train through hyperparameters. Testing the model identified mitogen‐activated protein kinase (MAPK1) as the best predicted gene using the radiomic features, yielding an AUC of 0.88, AUC_PR of 0.94 and *R* ^2^ of 0.5 at *p* < 0.002 (Figures 4d and 8). Wavelet frequency and 3D gray‐level based radiomic feature‐classes contributed the most to the classification of *MAPK1* expression into high and low expression categories, as depicted in the SHAP‐score distribution plot (Figure 9), indicating the potential of these imaging markers to act as surrogates for *MAPK1* expression.

Lastly, the exploration of the interaction between *BCL2L1* and *MAPK1* in several biological pathways using STRING‐DB indicated their co‐expression in the following pathways: ‘EGFR tyrosine kinase inhibitor resistance’ (False discovery rate [FDR] = 0.004), ‘Platinum Drug Resistance’ (FDR = 0.04), ‘Intrinsic Pathway for Apoptosis’ (FDR = 0.02), and PIK3‐AKT signaling pathway (FDR = 0.02) (Figure S3).

## 4 Discussion

The present study makes two substantial advances in the field of radiogenomics. First, we combined two radiogenomic datasets, thereby addressing a known challenge in radiogenomics for ML models: the lack of large data sets, which represents a substantial challenge owing to continual advances in radiomic, genomic, and imaging technologies [7]. Secondly, we show that radiomic features extracted from tumor ROIs on SOC images can be used as surrogate biomarkers for the individual expression of several genes known to play key roles in NSCLC and other human malignancies. We built both regression (MTEN) and classification‐based (multi‐ and single‐task RF classifiers) ML models. We detected significantly elevated expression of the gene *SLC35C1* on the old cohort. As separate validation between the two cohorts failed owing to skewness in the distribution of gene expression, we subsequently binarized gene expression (i.e., low vs. high) using its median expression in individual cohorts, and then batch‐normalized cohort‐specific radiomic features, an effective strategy to mitigate ‘center effect’, variability between temporally or spatially distinct datasets, and to increase generalizability for classification [43]. The binarized and batch‐normalized cohorts were combined to form a homogenized combined cohort, which was split into new training and testing cohorts representative of a heterogeneous mix from our original (i.e., temporally separated) cohorts. The training cohort was used to build an RF classifier, which classified the *SLC35C1* gene into high and low expression categories using several radiomic feature classes. These features exhibited considerable SHAP values, indicating the importance of these features as surrogates for predicting *SLC35C1* expression in our NSCLC cohort.

*SLC35C1*—an immunological and prognostic biomarker that has been shown to play a key role in multiple types of cancer—encodes GDP‐amylose transporter protein 1 and is involved in the transport of GDP‐fucose from the cytosol to cellular structures involved in secretion, such as the Golgi apparatus, endoplasmic reticulum and, endosomes [24]. Furthermore, *SLC35C1* expression has been previously found to be elevated in lung tumors compared to normal tissues in the TCGA cohort, and was also correlated with the tumor microenvironment and tumor molecular burden, microsatellite instability, and antitumor drug sensitivity in cancer [24]. The inhibition of *SLC35C1* in glioma cells increased tumor cell proliferation, migration, and invasion [24]. Additionally, elevated *SLC35C1* expression is known to be a key factor for increased fucosylation in hepatocellular carcinoma (HCC), and thus could be a potential target for the treatment and diagnosis of HCC [25]. In NSCLC, the modulation in expression of the genes in fuscosylation pathway have been associated with a poor prognosis and metastasis [26].

Our analysis of 33 target genes known to be important drivers of NSCLC classified *BCL2L1* and *MAPK1*, indicating that several imaging features from lung CT scans in a combined NSCLC cohort predicted the individual binarized expression of *BCL2L1* and *MAPK1*. *BCL2L1* is an anti‐apoptotic member of the well‐known family of BCL2 apoptotic regulatory proteins and is a promising prognostic biomarker and drug target in NSCLC [27, 28, 29]. MAPK1 (ERK2) is in the extracellular signal‐regulated kinase (ERK) subfamily of MAPKs, where ERK signaling has been referred to as “a master regulator of cell behavior, life, and fate” [30]. MAPK1/ERK2 has been implicated in myriad cancers through involvement in key pathways and via miRNA regulation [31, 32, 33], and has been implicated as an oncogene during NSCLC progression and significantly promoted the proliferation, migration, and invasion of NSCLC cell lines in vitro [34]. Furthermore, ERK signaling has been shown to upregulate anti‐apoptotic proteins by regulating the expression of *BCL2* and *BCL2L1* [30, 35, 36]. Our findings that both *BCL2L1* and *MAPK1* are co‐expressed in key biological pathways related to drug resistance, apoptosis and PIK3‐AKT signaling are in agreement with other studies in both NSCLC and other cancer types [27, 37, 38, 39, 40]. The literature suggests that the efficacy of the chemical agents G‐963 and GDC‐0941, which target MAPK and PI3K pathways, respectively, can be improved by the addition of a BCL‐2 family inhibitor (i.e., navitoclax [ABT‐263]), further supporting the co‐expression of these genes [41]. Additionally, the Hippo Pathway effector YAP1 (yes‐associated protein) has been shown to mediate resistance to RAF–MEK inhibitor therapy in NSCLC by suppressing the gene product of *BCL2L1*, the anti‐apoptotic protein BCL‐xL, together with MAPK signaling [42], highlighting the potential for the radiomic features identified in the present study to be used as non‐invasive, surrogate markers of *BCL2L1* and *MAPK1* and to ultimately predict potential resistance to therapy in NSCLC patients.

From a statistical standpoint, the Pearson‐based correlation and Hierarchical clustering methods used in our study define linear relationships between radiomic and gene‐expression features, which coheres with the method used in several radiogenomic studies presented in literature [11, 12, 13, 16]. However, studying non‐linear relationships between these features may allude to additional radiogenomic associations in NSCLC, indicating a limitation of our study.

In conclusion, we show that heterogeneous radiogenomic cohorts can be effectively combined to predict the binarized expression of individual genes from several radiomic features using multiple ML models with a high degree of AUC. Furthermore, our findings, in conjunction with considerable biological and experimental evidence in the literature, strengthens the argument that certain radiomic features from routine radiologic images can be used as surrogate predictors of the expression of key genes in NSCLC (i.e., *SLC35C1*, *BCL2L1* and *MAPK1*), which in turn could serve as biomarkers to predict clinical factors such as tumor molecular burden, response to therapies and metastatic potential. Our models also showcase the need to further research for the role of these genes in the progression of NSCLC. Larger datasets using images and gene expression data in collaboration with multiple hospitals and clinics will be required to further validate these findings to facilitate the translation of our findings into clinical oncology workflows, increase access to personalized medicine, and ultimately improve outcomes for NSCLC patients.

## Author Contributions

**Shrey S. Sukhadia:** conceptualization (equal), data curation (equal), formal analysis (equal), investigation (equal), methodology (equal), project administration (equal), resources (equal), software (equal), validation (equal), visualization (equal), writing – original draft (equal), writing – review and editing (equal). **Christopher Sadee:** data curation (equal), resources (equal), visualization (equal), writing – review and editing (equal). **Olivier Gevaert:** resources (equal), supervision (equal), writing – review and editing (equal). **Shivashankar H. Nagaraj:** resources (equal), supervision (equal), visualization (equal), writing – review and editing (equal).

## Ethics Statement

The current study was approved by the Institutional Review Board (IRB): IRB‐64916 at Stanford University. Informed Consent: The IRB waived the informed consent. Registry and the registration no. of the study/trial: N/A. Animal studies: N/A.

## Conflicts of Interest

The authors declare no conflicts of interest.

## Supporting information


Data S1.

## Data Availability

The lung radiomic and gene expression data used in the study are available at the github page of IMAGENE (https://github.com/skr1/Imagene) as CSV files named ‘Lung_radiomic_features_old_cohort_n116.csv’, ‘Lung_gene_expressions_old_cohort_n116.csv’, ‘Lung_radiomic_features_new_cohort_n44.csv,’ and ‘Lung_gene_expression_new_cohort_n44.csv’.
