# Supplementary material for: Machine Learning Enabled Prediction of Biologically Relevant Gene Expression Using CT‐Based Radiomic Features in Non‐Small Cell Lung Cancer
Source: Cancer Med. 2024 Dec 24;13(24):e70509. doi: 10.1002/cam4.70509 (PMC11667219; doi:10.1002/cam4.70509)

**Supplementary Table S1:**

List of 137 genes that correlated significantly (|r|>0.5, FDR adjusted *p-value* < 0.05) with 211 imaging features mentioned in Table S2 below.

| ABCC5 | HIBADH | SERPINB1 |
| --- | --- | --- |
| ABR | HIST1H2AC | SGTA |
| ACSS2 | IGF1R | SIPA1L3 |
| ALDH1A1 | IMPAD1 | SLC16A3 |
| ANKRD36 | ITGA2 | SLC25A43 |
| AP1B1 | ITPR2 | SLC35C1 |
| ARFGAP2 | JDP2 | SLC9A3R1 |
| ARHGEF11 | KDM5A | SMARCA4 |
| ASAP3 | LACTB2 | SMURF2 |
| ATP2B4 | LRPPRC | SNHG6 |
| BAZ2B | MAD1L1 | SNRPB2 |
| BZW1 | MAP1B | SNX30 |
| CAPRIN1 | MAPK6 | SPAST |
| CAPZA2 | MMS19 | SRP54 |
| CARHSP1 | MTDH | STUB1 |
| CC2D1B | MYO19 | TATDN1 |
| CD164 | NCOA3 | TGM2 |
| CDK5RAP1 | NDUFV1 | TIA1 |
| CFH | NNMT | TIAM1 |
| CPSF3 | NPC2 | TM7SF3 |
| DCUN1D4 | NUMA1 | TNFRSF21 |
| DDX60 | NUP107 | TNS3 |
| DHFR | NUP85 | TP53I3 |
| DNM2 | PACS2 | TPD52 |
| DNMT1 | PAG1 | TRAM1 |
| DPY19L1 | PAM | TRIM2 |
| EHD1 | PDE4D | TRIP10 |
| EIF3A | PDGFRA | TRPM7 |
| EIF5A | PITPNC1 | UQCRH |
| EPHA4 | PLIN3 | USP36 |
| ERC1 | PLXND1 | USP46 |
| EXOC3 | POLD4 | WARS2 |
| EYA3 | PPP6R2 | WHSC1L1 |
| FAF1 | PRCC | WIPI1 |
| FAF2 | PRKCI | ZDHHC4 |
| FAM168A | R3HDM2 | ZNF121 |
| FAM172A | RAB31 | ZNF7] |
| FAM98A | RAB3GAP1 |  |
| FMR1 | RALB |  |
| GATC | RALY |  |
| GDAP2 | RASSF3 |  |
| GLCE | RBM15 |  |
| GLRX3 | RNF149 |  |
| GNE | RNF185 |  |
| GOLGA3 | RPS24 |  |
| GOLT1B | RPS4X |  |
| GPR108 | RPS6KA4 |  |
| GRPEL1 | RPS6KB1 |  |
| GSTO1 | SAV1 |  |
| HERC3 | SBF2 |  |

**Supplementary Table S2:**

List of 211 imaging features that correlated significantly (|r|>0.5, FDR adjusted *p-value* < 0.05) with 137 genes mentioned in Table S1 above.

| exponential_firstorder_90Percentile | original_glcm_ClusterShade |
| --- | --- |
| exponential_firstorder_Entropy | original_gldm_DependenceNonUniformity |
| exponential_firstorder_Kurtosis | original_gldm_DependenceNonUniformityNormalized |
| exponential_firstorder_Skewness | original_gldm_LargeDependenceLowGrayLevelEmphasis |
| exponential_firstorder_Uniformity | original_gldm_LowGrayLevelEmphasis |
| exponential_glcm_ClusterProminence | original_glrlm_LongRunEmphasis |
| exponential_glcm_ClusterShade | original_glrlm_LongRunHighGrayLevelEmphasis |
| exponential_glcm_SumEntropy | original_glrlm_LongRunLowGrayLevelEmphasis |
| exponential_gldm_DependenceNonUniformityNormalized | original_glrlm_LowGrayLevelRunEmphasis |
| exponential_glrlm_GrayLevelNonUniformityNormalized | original_glrlm_RunVariance |
| exponential_glrlm_LongRunEmphasis | original_glrlm_ShortRunLowGrayLevelEmphasis |
| exponential_glrlm_LongRunHighGrayLevelEmphasis | original_glszm_LargeAreaLowGrayLevelEmphasis |
| exponential_glrlm_LongRunLowGrayLevelEmphasis | original_ngtdm_Busyness |
| exponential_glrlm_RunVariance | square_firstorder_90Percentile |
| exponential_glszm_LargeAreaEmphasis | square_firstorder_Entropy |
| exponential_glszm_LargeAreaHighGrayLevelEmphasis | square_firstorder_Kurtosis |
| exponential_glszm_LargeAreaLowGrayLevelEmphasis | square_firstorder_Skewness |
| exponential_glszm_SmallAreaLowGrayLevelEmphasis | square_firstorder_Uniformity |
| exponential_glszm_ZoneVariance | square_glcm_ClusterProminence |
| exponential_ngtdm_Coarseness | square_glcm_ClusterShade |
| exponential_ngtdm_Strength | square_glcm_InverseVariance |
| gradient_firstorder_Kurtosis | square_glcm_JointEnergy |
| gradient_firstorder_Skewness | square_glcm_MaximumProbability |
| gradient_glcm_ClusterProminence | square_glcm_SumEntropy |
| gradient_gldm_DependenceNonUniformityNormalized | square_gldm_DependenceEntropy |
| gradient_gldm_SmallDependenceHighGrayLevelEmphasis | square_gldm_LargeDependenceEmphasis |
| gradient_glszm_GrayLevelNonUniformity | square_gldm_LargeDependenceLowGrayLevelEmphasis |
| gradient_ngtdm_Strength | square_gldm_LowGrayLevelEmphasis |
| lbp-3D-k_firstorder_Kurtosis | square_glrlm_GrayLevelNonUniformityNormalized |
| lbp-3D-k_gldm_DependenceEntropy | square_glrlm_LongRunEmphasis |
| lbp-3D-k_gldm_DependenceNonUniformityNormalized | square_glrlm_LongRunLowGrayLevelEmphasis |
| lbp-3D-k_glrlm_LongRunEmphasis | square_glrlm_LowGrayLevelRunEmphasis |
| lbp-3D-k_glrlm_LongRunHighGrayLevelEmphasis | square_glrlm_RunVariance |
| lbp-3D-k_glrlm_LongRunLowGrayLevelEmphasis | square_glrlm_ShortRunEmphasis |
| lbp-3D-k_glrlm_RunVariance | square_ngtdm_Busyness |
| lbp-3D-k_glszm_HighGrayLevelZoneEmphasis | square_ngtdm_Strength |
| lbp-3D-k_glszm_LowGrayLevelZoneEmphasis | squareroot_firstorder_90Percentile |
| lbp-3D-k_ngtdm_Coarseness | squareroot_firstorder_Kurtosis |
| lbp-3D-k_ngtdm_Strength | squareroot_gldm_DependenceNonUniformityNormalized |
| lbp-3D-m1_glszm_GrayLevelNonUniformity | squareroot_glrlm_LongRunEmphasis |
| lbp-3D-m1_glszm_SizeZoneNonUniformityNormalized | squareroot_glrlm_LongRunLowGrayLevelEmphasis |
| lbp-3D-m1_glszm_SmallAreaEmphasis | squareroot_glszm_LargeAreaLowGrayLevelEmphasis |
| lbp-3D-m1_glszm_SmallAreaHighGrayLevelEmphasis | squareroot_ngtdm_Strength |
| lbp-3D-m1_glszm_SmallAreaLowGrayLevelEmphasis | wavelet-HHH_firstorder_Kurtosis |
| lbp-3D-m1_glszm_ZoneEntropy | wavelet-HHH_firstorder_Mean |
| lbp-3D-m1_glszm_ZoneVariance | wavelet-HHH_glszm_LargeAreaEmphasis |
| lbp-3D-m2_glszm_GrayLevelNonUniformity | wavelet-HHH_glszm_LargeAreaHighGrayLevelEmphasis |
| lbp-3D-m2_glszm_SizeZoneNonUniformityNormalized | wavelet-HHH_glszm_LargeAreaLowGrayLevelEmphasis |
| lbp-3D-m2_glszm_SmallAreaEmphasis | wavelet-HHH_glszm_ZoneVariance |
| lbp-3D-m2_glszm_SmallAreaHighGrayLevelEmphasis | wavelet-HHH_ngtdm_Busyness |
| lbp-3D-m2_glszm_SmallAreaLowGrayLevelEmphasis | wavelet-HHH_ngtdm_Strength |
| lbp-3D-m2_glszm_ZoneEntropy | wavelet-HHL_glcm_ClusterProminence |
| lbp-3D-m2_glszm_ZoneVariance | wavelet-HHL_glcm_ClusterShade |
| log-sigma-1-0-mm-3D_firstorder_Kurtosis | wavelet-HHL_glszm_LargeAreaEmphasis |
| log-sigma-1-0-mm-3D_glcm_Idmn | wavelet-HHL_glszm_LargeAreaLowGrayLevelEmphasis |
| log-sigma-1-0-mm-3D_ngtdm_Contrast | wavelet-HHL_glszm_SmallAreaLowGrayLevelEmphasis |
| log-sigma-2-0-mm-3D_gldm_LowGrayLevelEmphasis | wavelet-HHL_glszm_ZoneVariance |
| log-sigma-2-0-mm-3D_glrlm_LowGrayLevelRunEmphasis | wavelet-HHL_ngtdm_Busyness |
| log-sigma-2-0-mm-3D_glrlm_ShortRunLowGrayLevelEmphasis | wavelet-HLH_firstorder_Kurtosis |
| log-sigma-2-0-mm-3D_glszm_LowGrayLevelZoneEmphasis | wavelet-HLH_firstorder_Mean |
| log-sigma-2-0-mm-3D_glszm_SmallAreaLowGrayLevelEmphasis | wavelet-HLH_firstorder_Skewness |
| log-sigma-2-0-mm-3D_glszm_ZonePercentage | wavelet-HLH_glcm_ClusterProminence |
| log-sigma-3-0-mm-3D_firstorder_Uniformity | wavelet-HLH_glcm_ClusterShade |
| log-sigma-3-0-mm-3D_glcm_Imc2 | wavelet-HLH_gldm_SmallDependenceHighGrayLevelEmphasis |
| log-sigma-3-0-mm-3D_glcm_JointEnergy | wavelet-HLH_gldm_SmallDependenceLowGrayLevelEmphasis |
| log-sigma-3-0-mm-3D_glrlm_GrayLevelNonUniformityNormalized | wavelet-HLH_glszm_LargeAreaEmphasis |
| log-sigma-3-0-mm-3D_glszm_SmallAreaLowGrayLevelEmphasis | wavelet-HLH_glszm_LargeAreaHighGrayLevelEmphasis |
| log-sigma-4-0-mm-3D_firstorder_Kurtosis | wavelet-HLH_glszm_LargeAreaLowGrayLevelEmphasis |
| log-sigma-4-0-mm-3D_firstorder_Uniformity | wavelet-HLH_glszm_ZoneVariance |
| log-sigma-4-0-mm-3D_glcm_Imc2 | wavelet-HLH_ngtdm_Busyness |
| log-sigma-4-0-mm-3D_glcm_JointEnergy | wavelet-HLL_firstorder_Kurtosis |
| log-sigma-4-0-mm-3D_gldm_LargeDependenceHighGrayLevelEmphasis | wavelet-HLL_firstorder_Median |
| log-sigma-4-0-mm-3D_glrlm_LongRunEmphasis | wavelet-HLL_glszm_LargeAreaLowGrayLevelEmphasis |
| log-sigma-4-0-mm-3D_glrlm_LongRunHighGrayLevelEmphasis | wavelet-HLL_ngtdm_Busyness |
| log-sigma-4-0-mm-3D_glrlm_LongRunLowGrayLevelEmphasis | wavelet-LHH_firstorder_Kurtosis |
| log-sigma-4-0-mm-3D_glszm_GrayLevelNonUniformity | wavelet-LHH_glszm_LargeAreaEmphasis |
| log-sigma-4-0-mm-3D_glszm_SizeZoneNonUniformity | wavelet-LHH_glszm_LargeAreaLowGrayLevelEmphasis |
| log-sigma-4-0-mm-3D_glszm_SmallAreaLowGrayLevelEmphasis | wavelet-LHH_glszm_ZoneVariance |
| log-sigma-5-0-mm-3D_firstorder_Kurtosis | wavelet-LHH_ngtdm_Busyness |
| log-sigma-5-0-mm-3D_firstorder_Skewness | wavelet-LHH_ngtdm_Strength |
| log-sigma-5-0-mm-3D_glcm_ClusterShade | wavelet-LHL_firstorder_Kurtosis |
| log-sigma-5-0-mm-3D_gldm_LargeDependenceHighGrayLevelEmphasis | wavelet-LHL_firstorder_Skewness |
| log-sigma-5-0-mm-3D_gldm_SmallDependenceLowGrayLevelEmphasis | wavelet-LHL_glcm_ClusterProminence |
| log-sigma-5-0-mm-3D_glrlm_LongRunLowGrayLevelEmphasis | wavelet-LHL_glszm_LargeAreaEmphasis |
| log-sigma-5-0-mm-3D_glszm_GrayLevelNonUniformity | wavelet-LHL_glszm_LargeAreaLowGrayLevelEmphasis |
| log-sigma-5-0-mm-3D_glszm_SizeZoneNonUniformity | wavelet-LHL_glszm_ZoneVariance |
| logarithm_firstorder_90Percentile | wavelet-LHL_ngtdm_Busyness |
| logarithm_firstorder_Kurtosis | wavelet-LLH_glcm_ClusterProminence |
| logarithm_glcm_ClusterShade | wavelet-LLH_gldm_LargeDependenceLowGrayLevelEmphasis |
| logarithm_glrlm_LongRunEmphasis | wavelet-LLH_gldm_LowGrayLevelEmphasis |
| logarithm_glrlm_LongRunLowGrayLevelEmphasis | wavelet-LLH_glrlm_LongRunLowGrayLevelEmphasis |
| logarithm_glrlm_RunVariance | wavelet-LLH_glrlm_LowGrayLevelRunEmphasis |
| logarithm_glszm_LargeAreaEmphasis | wavelet-LLH_glszm_LargeAreaLowGrayLevelEmphasis |
| logarithm_glszm_LargeAreaLowGrayLevelEmphasis | wavelet-LLH_glszm_SmallAreaLowGrayLevelEmphasis |
| logarithm_glszm_ZoneVariance | wavelet-LLH_ngtdm_Busyness |
| logarithm_ngtdm_Complexity | wavelet-LLH_ngtdm_Contrast |
| original_firstorder_90Percentile | wavelet-LLL_firstorder_90Percentile |
| original_firstorder_Kurtosis | wavelet-LLL_firstorder_Kurtosis |
| original_firstorder_Skewness | wavelet-LLL_firstorder_Skewness |
| original_glcm_ClusterProminence | wavelet-LLL_glcm_ClusterProminence |
| original_glcm_ClusterShade | wavelet-LLL_glcm_ClusterShade |
| original_gldm_DependenceNonUniformity | wavelet-LLL_glcm_JointEnergy |
| original_gldm_DependenceNonUniformityNormalized | wavelet-LLL_gldm_LargeDependenceLowGrayLevelEmphasis |
| original_gldm_LargeDependenceLowGrayLevelEmphasis | wavelet-LLL_gldm_LowGrayLevelEmphasis |
| original_gldm_LowGrayLevelEmphasis | wavelet-LLL_glrlm_GrayLevelNonUniformityNormalized |
| original_glrlm_LongRunEmphasis | wavelet-LLL_glrlm_LongRunEmphasis |
| original_glrlm_LongRunHighGrayLevelEmphasis | wavelet-LLL_glrlm_LongRunHighGrayLevelEmphasis |
| original_glrlm_LongRunLowGrayLevelEmphasis | wavelet-LLL_glrlm_LongRunLowGrayLevelEmphasis |
| original_glrlm_LowGrayLevelRunEmphasis | wavelet-LLL_glrlm_LowGrayLevelRunEmphasis |
| original_glrlm_RunVariance | wavelet-LLL_glrlm_RunVariance |
| original_glrlm_ShortRunLowGrayLevelEmphasis | wavelet-LLL_glszm_LargeAreaLowGrayLevelEmphasis] |

**Supplementary Table S3:**

1522 radiomic features that got retained post interclass correlation filtering are as displayed in the table below:

| **Feature Class** | **Features** |
| --- | --- |
| original | original_shape_Elongation |
| original | original_shape_Flatness |
| original | original_shape_MajorAxisLength |
| original | original_shape_Maximum2DDiameterColumn |
| original | original_shape_Maximum2DDiameterRow |
| original | original_shape_Maximum2DDiameterSlice |
| original | original_shape_Maximum3DDiameter |
| original | original_shape_Sphericity |
| original | original_shape_SurfaceVolumeRatio |
| original | original_firstorder_10Percentile |
| original | original_firstorder_90Percentile |
| original | original_firstorder_Entropy |
| original | original_firstorder_Kurtosis |
| original | original_firstorder_Maximum |
| original | original_firstorder_Minimum |
| original | original_firstorder_Range |
| original | original_firstorder_Skewness |
| original | original_firstorder_Uniformity |
| original | original_glcm_Autocorrelation |
| original | original_glcm_ClusterProminence |
| original | original_glcm_ClusterShade |
| original | original_glcm_Correlation |
| original | original_glcm_Id |
| original | original_glcm_Idm |
| original | original_glcm_Idmn |
| original | original_glcm_Idn |
| original | original_glcm_Imc1 |
| original | original_glcm_Imc2 |
| original | original_glcm_InverseVariance |
| original | original_glcm_JointAverage |
| original | original_glcm_JointEnergy |
| original | original_glcm_MCC |
| original | original_glcm_MaximumProbability |
| original | original_glcm_SumAverage |
| original | original_glcm_SumEntropy |
| original | original_gldm_DependenceEntropy |
| original | original_gldm_DependenceNonUniformity |
| original | original_gldm_DependenceNonUniformityNormalized |
| original | original_gldm_DependenceVariance |
| original | original_gldm_HighGrayLevelEmphasis |
| original | original_gldm_LargeDependenceEmphasis |
| original | original_gldm_LargeDependenceHighGrayLevelEmphasis |
| original | original_gldm_LargeDependenceLowGrayLevelEmphasis |
| original | original_gldm_LowGrayLevelEmphasis |
| original | original_gldm_SmallDependenceHighGrayLevelEmphasis |
| original | original_gldm_SmallDependenceLowGrayLevelEmphasis |
| original | original_glrlm_GrayLevelNonUniformityNormalized |
| original | original_glrlm_GrayLevelVariance |
| original | original_glrlm_HighGrayLevelRunEmphasis |
| original | original_glrlm_LongRunEmphasis |
| original | original_glrlm_LongRunHighGrayLevelEmphasis |
| original | original_glrlm_LongRunLowGrayLevelEmphasis |
| original | original_glrlm_LowGrayLevelRunEmphasis |
| original | original_glrlm_RunEntropy |
| original | original_glrlm_RunPercentage |
| original | original_glrlm_RunVariance |
| original | original_glrlm_ShortRunEmphasis |
| original | original_glrlm_ShortRunHighGrayLevelEmphasis |
| original | original_glrlm_ShortRunLowGrayLevelEmphasis |
| original | original_glszm_GrayLevelNonUniformityNormalized |
| original | original_glszm_GrayLevelVariance |
| original | original_glszm_HighGrayLevelZoneEmphasis |
| original | original_glszm_LargeAreaLowGrayLevelEmphasis |
| original | original_glszm_LowGrayLevelZoneEmphasis |
| original | original_glszm_SizeZoneNonUniformityNormalized |
| original | original_glszm_SmallAreaEmphasis |
| original | original_glszm_SmallAreaHighGrayLevelEmphasis |
| original | original_glszm_SmallAreaLowGrayLevelEmphasis |
| original | original_glszm_ZoneEntropy |
| original | original_glszm_ZonePercentage |
| original | original_ngtdm_Busyness |
| original | original_ngtdm_Complexity |
| original | original_ngtdm_Contrast |
| original | original_ngtdm_Strength |
| log-sigma-1-0-mm-3D | log-sigma-1-0-mm-3D_firstorder_10Percentile |
| log-sigma-1-0-mm-3D | log-sigma-1-0-mm-3D_firstorder_90Percentile |
| log-sigma-1-0-mm-3D | log-sigma-1-0-mm-3D_firstorder_Kurtosis |
| log-sigma-1-0-mm-3D | log-sigma-1-0-mm-3D_firstorder_Maximum |
| log-sigma-1-0-mm-3D | log-sigma-1-0-mm-3D_firstorder_Mean |
| log-sigma-1-0-mm-3D | log-sigma-1-0-mm-3D_firstorder_Median |
| log-sigma-1-0-mm-3D | log-sigma-1-0-mm-3D_firstorder_Minimum |
| log-sigma-1-0-mm-3D | log-sigma-1-0-mm-3D_firstorder_Range |
| log-sigma-1-0-mm-3D | log-sigma-1-0-mm-3D_firstorder_Skewness |
| log-sigma-1-0-mm-3D | log-sigma-1-0-mm-3D_firstorder_Uniformity |
| log-sigma-1-0-mm-3D | log-sigma-1-0-mm-3D_glcm_Autocorrelation |
| log-sigma-1-0-mm-3D | log-sigma-1-0-mm-3D_glcm_ClusterProminence |
| log-sigma-1-0-mm-3D | log-sigma-1-0-mm-3D_glcm_ClusterShade |
| log-sigma-1-0-mm-3D | log-sigma-1-0-mm-3D_glcm_Correlation |
| log-sigma-1-0-mm-3D | log-sigma-1-0-mm-3D_glcm_Id |
| log-sigma-1-0-mm-3D | log-sigma-1-0-mm-3D_glcm_Idm |
| log-sigma-1-0-mm-3D | log-sigma-1-0-mm-3D_glcm_Idmn |
| log-sigma-1-0-mm-3D | log-sigma-1-0-mm-3D_glcm_Idn |
| log-sigma-1-0-mm-3D | log-sigma-1-0-mm-3D_glcm_Imc1 |
| log-sigma-1-0-mm-3D | log-sigma-1-0-mm-3D_glcm_Imc2 |
| log-sigma-1-0-mm-3D | log-sigma-1-0-mm-3D_glcm_InverseVariance |
| log-sigma-1-0-mm-3D | log-sigma-1-0-mm-3D_glcm_JointAverage |
| log-sigma-1-0-mm-3D | log-sigma-1-0-mm-3D_glcm_JointEnergy |
| log-sigma-1-0-mm-3D | log-sigma-1-0-mm-3D_glcm_MCC |
| log-sigma-1-0-mm-3D | log-sigma-1-0-mm-3D_glcm_MaximumProbability |
| log-sigma-1-0-mm-3D | log-sigma-1-0-mm-3D_glcm_SumAverage |
| log-sigma-1-0-mm-3D | log-sigma-1-0-mm-3D_gldm_DependenceEntropy |
| log-sigma-1-0-mm-3D | log-sigma-1-0-mm-3D_gldm_DependenceNonUniformityNormalized |
| log-sigma-1-0-mm-3D | log-sigma-1-0-mm-3D_gldm_DependenceVariance |
| log-sigma-1-0-mm-3D | log-sigma-1-0-mm-3D_gldm_HighGrayLevelEmphasis |
| log-sigma-1-0-mm-3D | log-sigma-1-0-mm-3D_gldm_LargeDependenceEmphasis |
| log-sigma-1-0-mm-3D | log-sigma-1-0-mm-3D_gldm_LargeDependenceHighGrayLevelEmphasis |
| log-sigma-1-0-mm-3D | log-sigma-1-0-mm-3D_gldm_LargeDependenceLowGrayLevelEmphasis |
| log-sigma-1-0-mm-3D | log-sigma-1-0-mm-3D_gldm_LowGrayLevelEmphasis |
| log-sigma-1-0-mm-3D | log-sigma-1-0-mm-3D_gldm_SmallDependenceEmphasis |
| log-sigma-1-0-mm-3D | log-sigma-1-0-mm-3D_gldm_SmallDependenceHighGrayLevelEmphasis |
| log-sigma-1-0-mm-3D | log-sigma-1-0-mm-3D_gldm_SmallDependenceLowGrayLevelEmphasis |
| log-sigma-1-0-mm-3D | log-sigma-1-0-mm-3D_glrlm_GrayLevelNonUniformityNormalized |
| log-sigma-1-0-mm-3D | log-sigma-1-0-mm-3D_glrlm_HighGrayLevelRunEmphasis |
| log-sigma-1-0-mm-3D | log-sigma-1-0-mm-3D_glrlm_LongRunEmphasis |
| log-sigma-1-0-mm-3D | log-sigma-1-0-mm-3D_glrlm_LongRunHighGrayLevelEmphasis |
| log-sigma-1-0-mm-3D | log-sigma-1-0-mm-3D_glrlm_LongRunLowGrayLevelEmphasis |
| log-sigma-1-0-mm-3D | log-sigma-1-0-mm-3D_glrlm_LowGrayLevelRunEmphasis |
| log-sigma-1-0-mm-3D | log-sigma-1-0-mm-3D_glrlm_RunEntropy |
| log-sigma-1-0-mm-3D | log-sigma-1-0-mm-3D_glrlm_RunPercentage |
| log-sigma-1-0-mm-3D | log-sigma-1-0-mm-3D_glrlm_RunVariance |
| log-sigma-1-0-mm-3D | log-sigma-1-0-mm-3D_glrlm_ShortRunHighGrayLevelEmphasis |
| log-sigma-1-0-mm-3D | log-sigma-1-0-mm-3D_glrlm_ShortRunLowGrayLevelEmphasis |
| log-sigma-1-0-mm-3D | log-sigma-1-0-mm-3D_glszm_GrayLevelNonUniformityNormalized |
| log-sigma-1-0-mm-3D | log-sigma-1-0-mm-3D_glszm_GrayLevelVariance |
| log-sigma-1-0-mm-3D | log-sigma-1-0-mm-3D_glszm_HighGrayLevelZoneEmphasis |
| log-sigma-1-0-mm-3D | log-sigma-1-0-mm-3D_glszm_LowGrayLevelZoneEmphasis |
| log-sigma-1-0-mm-3D | log-sigma-1-0-mm-3D_glszm_SizeZoneNonUniformityNormalized |
| log-sigma-1-0-mm-3D | log-sigma-1-0-mm-3D_glszm_SmallAreaEmphasis |
| log-sigma-1-0-mm-3D | log-sigma-1-0-mm-3D_glszm_SmallAreaHighGrayLevelEmphasis |
| log-sigma-1-0-mm-3D | log-sigma-1-0-mm-3D_glszm_SmallAreaLowGrayLevelEmphasis |
| log-sigma-1-0-mm-3D | log-sigma-1-0-mm-3D_glszm_ZoneEntropy |
| log-sigma-1-0-mm-3D | log-sigma-1-0-mm-3D_glszm_ZonePercentage |
| log-sigma-1-0-mm-3D | log-sigma-1-0-mm-3D_ngtdm_Coarseness |
| log-sigma-1-0-mm-3D | log-sigma-1-0-mm-3D_ngtdm_Complexity |
| log-sigma-1-0-mm-3D | log-sigma-1-0-mm-3D_ngtdm_Contrast |
| log-sigma-1-0-mm-3D | log-sigma-1-0-mm-3D_ngtdm_Strength |
| log-sigma-2-0-mm-3D | log-sigma-2-0-mm-3D_firstorder_10Percentile |
| log-sigma-2-0-mm-3D | log-sigma-2-0-mm-3D_firstorder_90Percentile |
| log-sigma-2-0-mm-3D | log-sigma-2-0-mm-3D_firstorder_Entropy |
| log-sigma-2-0-mm-3D | log-sigma-2-0-mm-3D_firstorder_InterquartileRange |
| log-sigma-2-0-mm-3D | log-sigma-2-0-mm-3D_firstorder_Kurtosis |
| log-sigma-2-0-mm-3D | log-sigma-2-0-mm-3D_firstorder_Maximum |
| log-sigma-2-0-mm-3D | log-sigma-2-0-mm-3D_firstorder_Mean |
| log-sigma-2-0-mm-3D | log-sigma-2-0-mm-3D_firstorder_Median |
| log-sigma-2-0-mm-3D | log-sigma-2-0-mm-3D_firstorder_Minimum |
| log-sigma-2-0-mm-3D | log-sigma-2-0-mm-3D_firstorder_Range |
| log-sigma-2-0-mm-3D | log-sigma-2-0-mm-3D_firstorder_RobustMeanAbsoluteDeviation |
| log-sigma-2-0-mm-3D | log-sigma-2-0-mm-3D_firstorder_RootMeanSquared |
| log-sigma-2-0-mm-3D | log-sigma-2-0-mm-3D_firstorder_Skewness |
| log-sigma-2-0-mm-3D | log-sigma-2-0-mm-3D_firstorder_Uniformity |
| log-sigma-2-0-mm-3D | log-sigma-2-0-mm-3D_firstorder_Variance |
| log-sigma-2-0-mm-3D | log-sigma-2-0-mm-3D_glcm_Autocorrelation |
| log-sigma-2-0-mm-3D | log-sigma-2-0-mm-3D_glcm_ClusterProminence |
| log-sigma-2-0-mm-3D | log-sigma-2-0-mm-3D_glcm_ClusterShade |
| log-sigma-2-0-mm-3D | log-sigma-2-0-mm-3D_glcm_ClusterTendency |
| log-sigma-2-0-mm-3D | log-sigma-2-0-mm-3D_glcm_Contrast |
| log-sigma-2-0-mm-3D | log-sigma-2-0-mm-3D_glcm_Correlation |
| log-sigma-2-0-mm-3D | log-sigma-2-0-mm-3D_glcm_Id |
| log-sigma-2-0-mm-3D | log-sigma-2-0-mm-3D_glcm_Idm |
| log-sigma-2-0-mm-3D | log-sigma-2-0-mm-3D_glcm_Idmn |
| log-sigma-2-0-mm-3D | log-sigma-2-0-mm-3D_glcm_Idn |
| log-sigma-2-0-mm-3D | log-sigma-2-0-mm-3D_glcm_Imc1 |
| log-sigma-2-0-mm-3D | log-sigma-2-0-mm-3D_glcm_Imc2 |
| log-sigma-2-0-mm-3D | log-sigma-2-0-mm-3D_glcm_JointAverage |
| log-sigma-2-0-mm-3D | log-sigma-2-0-mm-3D_glcm_JointEnergy |
| log-sigma-2-0-mm-3D | log-sigma-2-0-mm-3D_glcm_MCC |
| log-sigma-2-0-mm-3D | log-sigma-2-0-mm-3D_glcm_MaximumProbability |
| log-sigma-2-0-mm-3D | log-sigma-2-0-mm-3D_glcm_SumAverage |
| log-sigma-2-0-mm-3D | log-sigma-2-0-mm-3D_glcm_SumEntropy |
| log-sigma-2-0-mm-3D | log-sigma-2-0-mm-3D_glcm_SumSquares |
| log-sigma-2-0-mm-3D | log-sigma-2-0-mm-3D_gldm_DependenceEntropy |
| log-sigma-2-0-mm-3D | log-sigma-2-0-mm-3D_gldm_DependenceNonUniformityNormalized |
| log-sigma-2-0-mm-3D | log-sigma-2-0-mm-3D_gldm_DependenceVariance |
| log-sigma-2-0-mm-3D | log-sigma-2-0-mm-3D_gldm_GrayLevelVariance |
| log-sigma-2-0-mm-3D | log-sigma-2-0-mm-3D_gldm_HighGrayLevelEmphasis |
| log-sigma-2-0-mm-3D | log-sigma-2-0-mm-3D_gldm_LargeDependenceEmphasis |
| log-sigma-2-0-mm-3D | log-sigma-2-0-mm-3D_gldm_LargeDependenceHighGrayLevelEmphasis |
| log-sigma-2-0-mm-3D | log-sigma-2-0-mm-3D_gldm_LargeDependenceLowGrayLevelEmphasis |
| log-sigma-2-0-mm-3D | log-sigma-2-0-mm-3D_gldm_LowGrayLevelEmphasis |
| log-sigma-2-0-mm-3D | log-sigma-2-0-mm-3D_gldm_SmallDependenceEmphasis |
| log-sigma-2-0-mm-3D | log-sigma-2-0-mm-3D_gldm_SmallDependenceHighGrayLevelEmphasis |
| log-sigma-2-0-mm-3D | log-sigma-2-0-mm-3D_gldm_SmallDependenceLowGrayLevelEmphasis |
| log-sigma-2-0-mm-3D | log-sigma-2-0-mm-3D_glrlm_GrayLevelNonUniformityNormalized |
| log-sigma-2-0-mm-3D | log-sigma-2-0-mm-3D_glrlm_GrayLevelVariance |
| log-sigma-2-0-mm-3D | log-sigma-2-0-mm-3D_glrlm_HighGrayLevelRunEmphasis |
| log-sigma-2-0-mm-3D | log-sigma-2-0-mm-3D_glrlm_LongRunEmphasis |
| log-sigma-2-0-mm-3D | log-sigma-2-0-mm-3D_glrlm_LongRunHighGrayLevelEmphasis |
| log-sigma-2-0-mm-3D | log-sigma-2-0-mm-3D_glrlm_LongRunLowGrayLevelEmphasis |
| log-sigma-2-0-mm-3D | log-sigma-2-0-mm-3D_glrlm_LowGrayLevelRunEmphasis |
| log-sigma-2-0-mm-3D | log-sigma-2-0-mm-3D_glrlm_RunEntropy |
| log-sigma-2-0-mm-3D | log-sigma-2-0-mm-3D_glrlm_RunPercentage |
| log-sigma-2-0-mm-3D | log-sigma-2-0-mm-3D_glrlm_RunVariance |
| log-sigma-2-0-mm-3D | log-sigma-2-0-mm-3D_glrlm_ShortRunEmphasis |
| log-sigma-2-0-mm-3D | log-sigma-2-0-mm-3D_glrlm_ShortRunHighGrayLevelEmphasis |
| log-sigma-2-0-mm-3D | log-sigma-2-0-mm-3D_glrlm_ShortRunLowGrayLevelEmphasis |
| log-sigma-2-0-mm-3D | log-sigma-2-0-mm-3D_glszm_GrayLevelNonUniformity |
| log-sigma-2-0-mm-3D | log-sigma-2-0-mm-3D_glszm_GrayLevelNonUniformityNormalized |
| log-sigma-2-0-mm-3D | log-sigma-2-0-mm-3D_glszm_GrayLevelVariance |
| log-sigma-2-0-mm-3D | log-sigma-2-0-mm-3D_glszm_HighGrayLevelZoneEmphasis |
| log-sigma-2-0-mm-3D | log-sigma-2-0-mm-3D_glszm_LowGrayLevelZoneEmphasis |
| log-sigma-2-0-mm-3D | log-sigma-2-0-mm-3D_glszm_SizeZoneNonUniformity |
| log-sigma-2-0-mm-3D | log-sigma-2-0-mm-3D_glszm_SizeZoneNonUniformityNormalized |
| log-sigma-2-0-mm-3D | log-sigma-2-0-mm-3D_glszm_SmallAreaEmphasis |
| log-sigma-2-0-mm-3D | log-sigma-2-0-mm-3D_glszm_SmallAreaHighGrayLevelEmphasis |
| log-sigma-2-0-mm-3D | log-sigma-2-0-mm-3D_glszm_SmallAreaLowGrayLevelEmphasis |
| log-sigma-2-0-mm-3D | log-sigma-2-0-mm-3D_glszm_ZoneEntropy |
| log-sigma-2-0-mm-3D | log-sigma-2-0-mm-3D_glszm_ZonePercentage |
| log-sigma-2-0-mm-3D | log-sigma-2-0-mm-3D_ngtdm_Complexity |
| log-sigma-2-0-mm-3D | log-sigma-2-0-mm-3D_ngtdm_Contrast |
| log-sigma-2-0-mm-3D | log-sigma-2-0-mm-3D_ngtdm_Strength |
| log-sigma-3-0-mm-3D | log-sigma-3-0-mm-3D_firstorder_10Percentile |
| log-sigma-3-0-mm-3D | log-sigma-3-0-mm-3D_firstorder_90Percentile |
| log-sigma-3-0-mm-3D | log-sigma-3-0-mm-3D_firstorder_Energy |
| log-sigma-3-0-mm-3D | log-sigma-3-0-mm-3D_firstorder_Entropy |
| log-sigma-3-0-mm-3D | log-sigma-3-0-mm-3D_firstorder_InterquartileRange |
| log-sigma-3-0-mm-3D | log-sigma-3-0-mm-3D_firstorder_Kurtosis |
| log-sigma-3-0-mm-3D | log-sigma-3-0-mm-3D_firstorder_Maximum |
| log-sigma-3-0-mm-3D | log-sigma-3-0-mm-3D_firstorder_MeanAbsoluteDeviation |
| log-sigma-3-0-mm-3D | log-sigma-3-0-mm-3D_firstorder_Mean |
| log-sigma-3-0-mm-3D | log-sigma-3-0-mm-3D_firstorder_Median |
| log-sigma-3-0-mm-3D | log-sigma-3-0-mm-3D_firstorder_Minimum |
| log-sigma-3-0-mm-3D | log-sigma-3-0-mm-3D_firstorder_Range |
| log-sigma-3-0-mm-3D | log-sigma-3-0-mm-3D_firstorder_RobustMeanAbsoluteDeviation |
| log-sigma-3-0-mm-3D | log-sigma-3-0-mm-3D_firstorder_RootMeanSquared |
| log-sigma-3-0-mm-3D | log-sigma-3-0-mm-3D_firstorder_Skewness |
| log-sigma-3-0-mm-3D | log-sigma-3-0-mm-3D_firstorder_TotalEnergy |
| log-sigma-3-0-mm-3D | log-sigma-3-0-mm-3D_firstorder_Uniformity |
| log-sigma-3-0-mm-3D | log-sigma-3-0-mm-3D_firstorder_Variance |
| log-sigma-3-0-mm-3D | log-sigma-3-0-mm-3D_glcm_Autocorrelation |
| log-sigma-3-0-mm-3D | log-sigma-3-0-mm-3D_glcm_ClusterProminence |
| log-sigma-3-0-mm-3D | log-sigma-3-0-mm-3D_glcm_ClusterShade |
| log-sigma-3-0-mm-3D | log-sigma-3-0-mm-3D_glcm_ClusterTendency |
| log-sigma-3-0-mm-3D | log-sigma-3-0-mm-3D_glcm_Contrast |
| log-sigma-3-0-mm-3D | log-sigma-3-0-mm-3D_glcm_Correlation |
| log-sigma-3-0-mm-3D | log-sigma-3-0-mm-3D_glcm_DifferenceEntropy |
| log-sigma-3-0-mm-3D | log-sigma-3-0-mm-3D_glcm_Id |
| log-sigma-3-0-mm-3D | log-sigma-3-0-mm-3D_glcm_Idm |
| log-sigma-3-0-mm-3D | log-sigma-3-0-mm-3D_glcm_Idmn |
| log-sigma-3-0-mm-3D | log-sigma-3-0-mm-3D_glcm_Idn |
| log-sigma-3-0-mm-3D | log-sigma-3-0-mm-3D_glcm_Imc1 |
| log-sigma-3-0-mm-3D | log-sigma-3-0-mm-3D_glcm_Imc2 |
| log-sigma-3-0-mm-3D | log-sigma-3-0-mm-3D_glcm_JointAverage |
| log-sigma-3-0-mm-3D | log-sigma-3-0-mm-3D_glcm_JointEnergy |
| log-sigma-3-0-mm-3D | log-sigma-3-0-mm-3D_glcm_JointEntropy |
| log-sigma-3-0-mm-3D | log-sigma-3-0-mm-3D_glcm_MCC |
| log-sigma-3-0-mm-3D | log-sigma-3-0-mm-3D_glcm_MaximumProbability |
| log-sigma-3-0-mm-3D | log-sigma-3-0-mm-3D_glcm_SumAverage |
| log-sigma-3-0-mm-3D | log-sigma-3-0-mm-3D_glcm_SumEntropy |
| log-sigma-3-0-mm-3D | log-sigma-3-0-mm-3D_glcm_SumSquares |
| log-sigma-3-0-mm-3D | log-sigma-3-0-mm-3D_gldm_DependenceEntropy |
| log-sigma-3-0-mm-3D | log-sigma-3-0-mm-3D_gldm_DependenceNonUniformityNormalized |
| log-sigma-3-0-mm-3D | log-sigma-3-0-mm-3D_gldm_DependenceVariance |
| log-sigma-3-0-mm-3D | log-sigma-3-0-mm-3D_gldm_GrayLevelVariance |
| log-sigma-3-0-mm-3D | log-sigma-3-0-mm-3D_gldm_HighGrayLevelEmphasis |
| log-sigma-3-0-mm-3D | log-sigma-3-0-mm-3D_gldm_LargeDependenceEmphasis |
| log-sigma-3-0-mm-3D | log-sigma-3-0-mm-3D_gldm_LargeDependenceHighGrayLevelEmphasis |
| log-sigma-3-0-mm-3D | log-sigma-3-0-mm-3D_gldm_LargeDependenceLowGrayLevelEmphasis |
| log-sigma-3-0-mm-3D | log-sigma-3-0-mm-3D_gldm_LowGrayLevelEmphasis |
| log-sigma-3-0-mm-3D | log-sigma-3-0-mm-3D_gldm_SmallDependenceEmphasis |
| log-sigma-3-0-mm-3D | log-sigma-3-0-mm-3D_gldm_SmallDependenceHighGrayLevelEmphasis |
| log-sigma-3-0-mm-3D | log-sigma-3-0-mm-3D_gldm_SmallDependenceLowGrayLevelEmphasis |
| log-sigma-3-0-mm-3D | log-sigma-3-0-mm-3D_glrlm_GrayLevelNonUniformityNormalized |
| log-sigma-3-0-mm-3D | log-sigma-3-0-mm-3D_glrlm_GrayLevelVariance |
| log-sigma-3-0-mm-3D | log-sigma-3-0-mm-3D_glrlm_HighGrayLevelRunEmphasis |
| log-sigma-3-0-mm-3D | log-sigma-3-0-mm-3D_glrlm_LongRunEmphasis |
| log-sigma-3-0-mm-3D | log-sigma-3-0-mm-3D_glrlm_LongRunHighGrayLevelEmphasis |
| log-sigma-3-0-mm-3D | log-sigma-3-0-mm-3D_glrlm_LongRunLowGrayLevelEmphasis |
| log-sigma-3-0-mm-3D | log-sigma-3-0-mm-3D_glrlm_LowGrayLevelRunEmphasis |
| log-sigma-3-0-mm-3D | log-sigma-3-0-mm-3D_glrlm_RunEntropy |
| log-sigma-3-0-mm-3D | log-sigma-3-0-mm-3D_glrlm_RunVariance |
| log-sigma-3-0-mm-3D | log-sigma-3-0-mm-3D_glrlm_ShortRunHighGrayLevelEmphasis |
| log-sigma-3-0-mm-3D | log-sigma-3-0-mm-3D_glrlm_ShortRunLowGrayLevelEmphasis |
| log-sigma-3-0-mm-3D | log-sigma-3-0-mm-3D_glszm_GrayLevelNonUniformity |
| log-sigma-3-0-mm-3D | log-sigma-3-0-mm-3D_glszm_GrayLevelNonUniformityNormalized |
| log-sigma-3-0-mm-3D | log-sigma-3-0-mm-3D_glszm_GrayLevelVariance |
| log-sigma-3-0-mm-3D | log-sigma-3-0-mm-3D_glszm_HighGrayLevelZoneEmphasis |
| log-sigma-3-0-mm-3D | log-sigma-3-0-mm-3D_glszm_LowGrayLevelZoneEmphasis |
| log-sigma-3-0-mm-3D | log-sigma-3-0-mm-3D_glszm_SizeZoneNonUniformity |
| log-sigma-3-0-mm-3D | log-sigma-3-0-mm-3D_glszm_SizeZoneNonUniformityNormalized |
| log-sigma-3-0-mm-3D | log-sigma-3-0-mm-3D_glszm_SmallAreaEmphasis |
| log-sigma-3-0-mm-3D | log-sigma-3-0-mm-3D_glszm_SmallAreaHighGrayLevelEmphasis |
| log-sigma-3-0-mm-3D | log-sigma-3-0-mm-3D_glszm_SmallAreaLowGrayLevelEmphasis |
| log-sigma-3-0-mm-3D | log-sigma-3-0-mm-3D_glszm_ZoneEntropy |
| log-sigma-3-0-mm-3D | log-sigma-3-0-mm-3D_glszm_ZonePercentage |
| log-sigma-3-0-mm-3D | log-sigma-3-0-mm-3D_ngtdm_Coarseness |
| log-sigma-3-0-mm-3D | log-sigma-3-0-mm-3D_ngtdm_Complexity |
| log-sigma-3-0-mm-3D | log-sigma-3-0-mm-3D_ngtdm_Contrast |
| log-sigma-3-0-mm-3D | log-sigma-3-0-mm-3D_ngtdm_Strength |
| log-sigma-4-0-mm-3D | log-sigma-4-0-mm-3D_firstorder_10Percentile |
| log-sigma-4-0-mm-3D | log-sigma-4-0-mm-3D_firstorder_90Percentile |
| log-sigma-4-0-mm-3D | log-sigma-4-0-mm-3D_firstorder_Energy |
| log-sigma-4-0-mm-3D | log-sigma-4-0-mm-3D_firstorder_Entropy |
| log-sigma-4-0-mm-3D | log-sigma-4-0-mm-3D_firstorder_InterquartileRange |
| log-sigma-4-0-mm-3D | log-sigma-4-0-mm-3D_firstorder_Kurtosis |
| log-sigma-4-0-mm-3D | log-sigma-4-0-mm-3D_firstorder_Maximum |
| log-sigma-4-0-mm-3D | log-sigma-4-0-mm-3D_firstorder_MeanAbsoluteDeviation |
| log-sigma-4-0-mm-3D | log-sigma-4-0-mm-3D_firstorder_Mean |
| log-sigma-4-0-mm-3D | log-sigma-4-0-mm-3D_firstorder_Median |
| log-sigma-4-0-mm-3D | log-sigma-4-0-mm-3D_firstorder_Minimum |
| log-sigma-4-0-mm-3D | log-sigma-4-0-mm-3D_firstorder_Range |
| log-sigma-4-0-mm-3D | log-sigma-4-0-mm-3D_firstorder_RobustMeanAbsoluteDeviation |
| log-sigma-4-0-mm-3D | log-sigma-4-0-mm-3D_firstorder_RootMeanSquared |
| log-sigma-4-0-mm-3D | log-sigma-4-0-mm-3D_firstorder_Skewness |
| log-sigma-4-0-mm-3D | log-sigma-4-0-mm-3D_firstorder_TotalEnergy |
| log-sigma-4-0-mm-3D | log-sigma-4-0-mm-3D_firstorder_Uniformity |
| log-sigma-4-0-mm-3D | log-sigma-4-0-mm-3D_firstorder_Variance |
| log-sigma-4-0-mm-3D | log-sigma-4-0-mm-3D_glcm_Autocorrelation |
| log-sigma-4-0-mm-3D | log-sigma-4-0-mm-3D_glcm_ClusterProminence |
| log-sigma-4-0-mm-3D | log-sigma-4-0-mm-3D_glcm_ClusterShade |
| log-sigma-4-0-mm-3D | log-sigma-4-0-mm-3D_glcm_ClusterTendency |
| log-sigma-4-0-mm-3D | log-sigma-4-0-mm-3D_glcm_Correlation |
| log-sigma-4-0-mm-3D | log-sigma-4-0-mm-3D_glcm_DifferenceEntropy |
| log-sigma-4-0-mm-3D | log-sigma-4-0-mm-3D_glcm_Id |
| log-sigma-4-0-mm-3D | log-sigma-4-0-mm-3D_glcm_Idm |
| log-sigma-4-0-mm-3D | log-sigma-4-0-mm-3D_glcm_Idmn |
| log-sigma-4-0-mm-3D | log-sigma-4-0-mm-3D_glcm_Idn |
| log-sigma-4-0-mm-3D | log-sigma-4-0-mm-3D_glcm_Imc1 |
| log-sigma-4-0-mm-3D | log-sigma-4-0-mm-3D_glcm_Imc2 |
| log-sigma-4-0-mm-3D | log-sigma-4-0-mm-3D_glcm_JointAverage |
| log-sigma-4-0-mm-3D | log-sigma-4-0-mm-3D_glcm_JointEnergy |
| log-sigma-4-0-mm-3D | log-sigma-4-0-mm-3D_glcm_JointEntropy |
| log-sigma-4-0-mm-3D | log-sigma-4-0-mm-3D_glcm_MCC |
| log-sigma-4-0-mm-3D | log-sigma-4-0-mm-3D_glcm_MaximumProbability |
| log-sigma-4-0-mm-3D | log-sigma-4-0-mm-3D_glcm_SumAverage |
| log-sigma-4-0-mm-3D | log-sigma-4-0-mm-3D_glcm_SumEntropy |
| log-sigma-4-0-mm-3D | log-sigma-4-0-mm-3D_glcm_SumSquares |
| log-sigma-4-0-mm-3D | log-sigma-4-0-mm-3D_gldm_DependenceEntropy |
| log-sigma-4-0-mm-3D | log-sigma-4-0-mm-3D_gldm_DependenceNonUniformityNormalized |
| log-sigma-4-0-mm-3D | log-sigma-4-0-mm-3D_gldm_DependenceVariance |
| log-sigma-4-0-mm-3D | log-sigma-4-0-mm-3D_gldm_GrayLevelVariance |
| log-sigma-4-0-mm-3D | log-sigma-4-0-mm-3D_gldm_HighGrayLevelEmphasis |
| log-sigma-4-0-mm-3D | log-sigma-4-0-mm-3D_gldm_LargeDependenceEmphasis |
| log-sigma-4-0-mm-3D | log-sigma-4-0-mm-3D_gldm_LargeDependenceHighGrayLevelEmphasis |
| log-sigma-4-0-mm-3D | log-sigma-4-0-mm-3D_gldm_LargeDependenceLowGrayLevelEmphasis |
| log-sigma-4-0-mm-3D | log-sigma-4-0-mm-3D_gldm_LowGrayLevelEmphasis |
| log-sigma-4-0-mm-3D | log-sigma-4-0-mm-3D_gldm_SmallDependenceEmphasis |
| log-sigma-4-0-mm-3D | log-sigma-4-0-mm-3D_gldm_SmallDependenceHighGrayLevelEmphasis |
| log-sigma-4-0-mm-3D | log-sigma-4-0-mm-3D_gldm_SmallDependenceLowGrayLevelEmphasis |
| log-sigma-4-0-mm-3D | log-sigma-4-0-mm-3D_glrlm_GrayLevelNonUniformityNormalized |
| log-sigma-4-0-mm-3D | log-sigma-4-0-mm-3D_glrlm_GrayLevelVariance |
| log-sigma-4-0-mm-3D | log-sigma-4-0-mm-3D_glrlm_HighGrayLevelRunEmphasis |
| log-sigma-4-0-mm-3D | log-sigma-4-0-mm-3D_glrlm_LongRunEmphasis |
| log-sigma-4-0-mm-3D | log-sigma-4-0-mm-3D_glrlm_LongRunHighGrayLevelEmphasis |
| log-sigma-4-0-mm-3D | log-sigma-4-0-mm-3D_glrlm_LongRunLowGrayLevelEmphasis |
| log-sigma-4-0-mm-3D | log-sigma-4-0-mm-3D_glrlm_LowGrayLevelRunEmphasis |
| log-sigma-4-0-mm-3D | log-sigma-4-0-mm-3D_glrlm_RunEntropy |
| log-sigma-4-0-mm-3D | log-sigma-4-0-mm-3D_glrlm_RunPercentage |
| log-sigma-4-0-mm-3D | log-sigma-4-0-mm-3D_glrlm_ShortRunEmphasis |
| log-sigma-4-0-mm-3D | log-sigma-4-0-mm-3D_glrlm_ShortRunHighGrayLevelEmphasis |
| log-sigma-4-0-mm-3D | log-sigma-4-0-mm-3D_glrlm_ShortRunLowGrayLevelEmphasis |
| log-sigma-4-0-mm-3D | log-sigma-4-0-mm-3D_glszm_GrayLevelNonUniformity |
| log-sigma-4-0-mm-3D | log-sigma-4-0-mm-3D_glszm_GrayLevelNonUniformityNormalized |
| log-sigma-4-0-mm-3D | log-sigma-4-0-mm-3D_glszm_GrayLevelVariance |
| log-sigma-4-0-mm-3D | log-sigma-4-0-mm-3D_glszm_HighGrayLevelZoneEmphasis |
| log-sigma-4-0-mm-3D | log-sigma-4-0-mm-3D_glszm_LowGrayLevelZoneEmphasis |
| log-sigma-4-0-mm-3D | log-sigma-4-0-mm-3D_glszm_SizeZoneNonUniformity |
| log-sigma-4-0-mm-3D | log-sigma-4-0-mm-3D_glszm_SizeZoneNonUniformityNormalized |
| log-sigma-4-0-mm-3D | log-sigma-4-0-mm-3D_glszm_SmallAreaEmphasis |
| log-sigma-4-0-mm-3D | log-sigma-4-0-mm-3D_glszm_SmallAreaHighGrayLevelEmphasis |
| log-sigma-4-0-mm-3D | log-sigma-4-0-mm-3D_glszm_SmallAreaLowGrayLevelEmphasis |
| log-sigma-4-0-mm-3D | log-sigma-4-0-mm-3D_glszm_ZoneEntropy |
| log-sigma-4-0-mm-3D | log-sigma-4-0-mm-3D_glszm_ZonePercentage |
| log-sigma-4-0-mm-3D | log-sigma-4-0-mm-3D_ngtdm_Coarseness |
| log-sigma-4-0-mm-3D | log-sigma-4-0-mm-3D_ngtdm_Complexity |
| log-sigma-4-0-mm-3D | log-sigma-4-0-mm-3D_ngtdm_Contrast |
| log-sigma-4-0-mm-3D | log-sigma-4-0-mm-3D_ngtdm_Strength |
| log-sigma-5-0-mm-3D | log-sigma-5-0-mm-3D_firstorder_10Percentile |
| log-sigma-5-0-mm-3D | log-sigma-5-0-mm-3D_firstorder_90Percentile |
| log-sigma-5-0-mm-3D | log-sigma-5-0-mm-3D_firstorder_Energy |
| log-sigma-5-0-mm-3D | log-sigma-5-0-mm-3D_firstorder_Entropy |
| log-sigma-5-0-mm-3D | log-sigma-5-0-mm-3D_firstorder_InterquartileRange |
| log-sigma-5-0-mm-3D | log-sigma-5-0-mm-3D_firstorder_Kurtosis |
| log-sigma-5-0-mm-3D | log-sigma-5-0-mm-3D_firstorder_Maximum |
| log-sigma-5-0-mm-3D | log-sigma-5-0-mm-3D_firstorder_MeanAbsoluteDeviation |
| log-sigma-5-0-mm-3D | log-sigma-5-0-mm-3D_firstorder_Mean |
| log-sigma-5-0-mm-3D | log-sigma-5-0-mm-3D_firstorder_Median |
| log-sigma-5-0-mm-3D | log-sigma-5-0-mm-3D_firstorder_Minimum |
| log-sigma-5-0-mm-3D | log-sigma-5-0-mm-3D_firstorder_Range |
| log-sigma-5-0-mm-3D | log-sigma-5-0-mm-3D_firstorder_RobustMeanAbsoluteDeviation |
| log-sigma-5-0-mm-3D | log-sigma-5-0-mm-3D_firstorder_RootMeanSquared |
| log-sigma-5-0-mm-3D | log-sigma-5-0-mm-3D_firstorder_Skewness |
| log-sigma-5-0-mm-3D | log-sigma-5-0-mm-3D_firstorder_TotalEnergy |
| log-sigma-5-0-mm-3D | log-sigma-5-0-mm-3D_firstorder_Uniformity |
| log-sigma-5-0-mm-3D | log-sigma-5-0-mm-3D_firstorder_Variance |
| log-sigma-5-0-mm-3D | log-sigma-5-0-mm-3D_glcm_Autocorrelation |
| log-sigma-5-0-mm-3D | log-sigma-5-0-mm-3D_glcm_ClusterProminence |
| log-sigma-5-0-mm-3D | log-sigma-5-0-mm-3D_glcm_ClusterShade |
| log-sigma-5-0-mm-3D | log-sigma-5-0-mm-3D_glcm_ClusterTendency |
| log-sigma-5-0-mm-3D | log-sigma-5-0-mm-3D_glcm_Contrast |
| log-sigma-5-0-mm-3D | log-sigma-5-0-mm-3D_glcm_Correlation |
| log-sigma-5-0-mm-3D | log-sigma-5-0-mm-3D_glcm_DifferenceAverage |
| log-sigma-5-0-mm-3D | log-sigma-5-0-mm-3D_glcm_DifferenceEntropy |
| log-sigma-5-0-mm-3D | log-sigma-5-0-mm-3D_glcm_DifferenceVariance |
| log-sigma-5-0-mm-3D | log-sigma-5-0-mm-3D_glcm_Id |
| log-sigma-5-0-mm-3D | log-sigma-5-0-mm-3D_glcm_Idm |
| log-sigma-5-0-mm-3D | log-sigma-5-0-mm-3D_glcm_Idmn |
| log-sigma-5-0-mm-3D | log-sigma-5-0-mm-3D_glcm_Idn |
| log-sigma-5-0-mm-3D | log-sigma-5-0-mm-3D_glcm_Imc1 |
| log-sigma-5-0-mm-3D | log-sigma-5-0-mm-3D_glcm_Imc2 |
| log-sigma-5-0-mm-3D | log-sigma-5-0-mm-3D_glcm_InverseVariance |
| log-sigma-5-0-mm-3D | log-sigma-5-0-mm-3D_glcm_JointAverage |
| log-sigma-5-0-mm-3D | log-sigma-5-0-mm-3D_glcm_JointEnergy |
| log-sigma-5-0-mm-3D | log-sigma-5-0-mm-3D_glcm_JointEntropy |
| log-sigma-5-0-mm-3D | log-sigma-5-0-mm-3D_glcm_MCC |
| log-sigma-5-0-mm-3D | log-sigma-5-0-mm-3D_glcm_MaximumProbability |
| log-sigma-5-0-mm-3D | log-sigma-5-0-mm-3D_glcm_SumAverage |
| log-sigma-5-0-mm-3D | log-sigma-5-0-mm-3D_glcm_SumEntropy |
| log-sigma-5-0-mm-3D | log-sigma-5-0-mm-3D_glcm_SumSquares |
| log-sigma-5-0-mm-3D | log-sigma-5-0-mm-3D_gldm_DependenceEntropy |
| log-sigma-5-0-mm-3D | log-sigma-5-0-mm-3D_gldm_DependenceNonUniformityNormalized |
| log-sigma-5-0-mm-3D | log-sigma-5-0-mm-3D_gldm_DependenceVariance |
| log-sigma-5-0-mm-3D | log-sigma-5-0-mm-3D_gldm_GrayLevelVariance |
| log-sigma-5-0-mm-3D | log-sigma-5-0-mm-3D_gldm_HighGrayLevelEmphasis |
| log-sigma-5-0-mm-3D | log-sigma-5-0-mm-3D_gldm_LargeDependenceEmphasis |
| log-sigma-5-0-mm-3D | log-sigma-5-0-mm-3D_gldm_LargeDependenceHighGrayLevelEmphasis |
| log-sigma-5-0-mm-3D | log-sigma-5-0-mm-3D_gldm_LargeDependenceLowGrayLevelEmphasis |
| log-sigma-5-0-mm-3D | log-sigma-5-0-mm-3D_gldm_LowGrayLevelEmphasis |
| log-sigma-5-0-mm-3D | log-sigma-5-0-mm-3D_gldm_SmallDependenceEmphasis |
| log-sigma-5-0-mm-3D | log-sigma-5-0-mm-3D_gldm_SmallDependenceHighGrayLevelEmphasis |
| log-sigma-5-0-mm-3D | log-sigma-5-0-mm-3D_gldm_SmallDependenceLowGrayLevelEmphasis |
| log-sigma-5-0-mm-3D | log-sigma-5-0-mm-3D_glrlm_GrayLevelNonUniformityNormalized |
| log-sigma-5-0-mm-3D | log-sigma-5-0-mm-3D_glrlm_GrayLevelVariance |
| log-sigma-5-0-mm-3D | log-sigma-5-0-mm-3D_glrlm_HighGrayLevelRunEmphasis |
| log-sigma-5-0-mm-3D | log-sigma-5-0-mm-3D_glrlm_LongRunHighGrayLevelEmphasis |
| log-sigma-5-0-mm-3D | log-sigma-5-0-mm-3D_glrlm_LongRunLowGrayLevelEmphasis |
| log-sigma-5-0-mm-3D | log-sigma-5-0-mm-3D_glrlm_LowGrayLevelRunEmphasis |
| log-sigma-5-0-mm-3D | log-sigma-5-0-mm-3D_glrlm_RunEntropy |
| log-sigma-5-0-mm-3D | log-sigma-5-0-mm-3D_glrlm_RunLengthNonUniformityNormalized |
| log-sigma-5-0-mm-3D | log-sigma-5-0-mm-3D_glrlm_RunPercentage |
| log-sigma-5-0-mm-3D | log-sigma-5-0-mm-3D_glrlm_ShortRunEmphasis |
| log-sigma-5-0-mm-3D | log-sigma-5-0-mm-3D_glrlm_ShortRunHighGrayLevelEmphasis |
| log-sigma-5-0-mm-3D | log-sigma-5-0-mm-3D_glrlm_ShortRunLowGrayLevelEmphasis |
| log-sigma-5-0-mm-3D | log-sigma-5-0-mm-3D_glszm_GrayLevelNonUniformity |
| log-sigma-5-0-mm-3D | log-sigma-5-0-mm-3D_glszm_GrayLevelNonUniformityNormalized |
| log-sigma-5-0-mm-3D | log-sigma-5-0-mm-3D_glszm_GrayLevelVariance |
| log-sigma-5-0-mm-3D | log-sigma-5-0-mm-3D_glszm_HighGrayLevelZoneEmphasis |
| log-sigma-5-0-mm-3D | log-sigma-5-0-mm-3D_glszm_LowGrayLevelZoneEmphasis |
| log-sigma-5-0-mm-3D | log-sigma-5-0-mm-3D_glszm_SizeZoneNonUniformity |
| log-sigma-5-0-mm-3D | log-sigma-5-0-mm-3D_glszm_SizeZoneNonUniformityNormalized |
| log-sigma-5-0-mm-3D | log-sigma-5-0-mm-3D_glszm_SmallAreaEmphasis |
| log-sigma-5-0-mm-3D | log-sigma-5-0-mm-3D_glszm_SmallAreaHighGrayLevelEmphasis |
| log-sigma-5-0-mm-3D | log-sigma-5-0-mm-3D_glszm_SmallAreaLowGrayLevelEmphasis |
| log-sigma-5-0-mm-3D | log-sigma-5-0-mm-3D_glszm_ZoneEntropy |
| log-sigma-5-0-mm-3D | log-sigma-5-0-mm-3D_glszm_ZonePercentage |
| log-sigma-5-0-mm-3D | log-sigma-5-0-mm-3D_ngtdm_Complexity |
| log-sigma-5-0-mm-3D | log-sigma-5-0-mm-3D_ngtdm_Contrast |
| log-sigma-5-0-mm-3D | log-sigma-5-0-mm-3D_ngtdm_Strength |
| wavelet-LLH | wavelet-LLH_firstorder_10Percentile |
| wavelet-LLH | wavelet-LLH_firstorder_90Percentile |
| wavelet-LLH | wavelet-LLH_firstorder_InterquartileRange |
| wavelet-LLH | wavelet-LLH_firstorder_Kurtosis |
| wavelet-LLH | wavelet-LLH_firstorder_Maximum |
| wavelet-LLH | wavelet-LLH_firstorder_Mean |
| wavelet-LLH | wavelet-LLH_firstorder_Median |
| wavelet-LLH | wavelet-LLH_firstorder_Minimum |
| wavelet-LLH | wavelet-LLH_firstorder_Range |
| wavelet-LLH | wavelet-LLH_firstorder_RobustMeanAbsoluteDeviation |
| wavelet-LLH | wavelet-LLH_firstorder_Skewness |
| wavelet-LLH | wavelet-LLH_firstorder_Uniformity |
| wavelet-LLH | wavelet-LLH_glcm_Autocorrelation |
| wavelet-LLH | wavelet-LLH_glcm_ClusterProminence |
| wavelet-LLH | wavelet-LLH_glcm_ClusterShade |
| wavelet-LLH | wavelet-LLH_glcm_Correlation |
| wavelet-LLH | wavelet-LLH_glcm_DifferenceVariance |
| wavelet-LLH | wavelet-LLH_glcm_Id |
| wavelet-LLH | wavelet-LLH_glcm_Idm |
| wavelet-LLH | wavelet-LLH_glcm_Idmn |
| wavelet-LLH | wavelet-LLH_glcm_Idn |
| wavelet-LLH | wavelet-LLH_glcm_Imc1 |
| wavelet-LLH | wavelet-LLH_glcm_Imc2 |
| wavelet-LLH | wavelet-LLH_glcm_InverseVariance |
| wavelet-LLH | wavelet-LLH_glcm_JointAverage |
| wavelet-LLH | wavelet-LLH_glcm_JointEnergy |
| wavelet-LLH | wavelet-LLH_glcm_MCC |
| wavelet-LLH | wavelet-LLH_glcm_MaximumProbability |
| wavelet-LLH | wavelet-LLH_glcm_SumAverage |
| wavelet-LLH | wavelet-LLH_gldm_DependenceEntropy |
| wavelet-LLH | wavelet-LLH_gldm_DependenceNonUniformityNormalized |
| wavelet-LLH | wavelet-LLH_gldm_DependenceVariance |
| wavelet-LLH | wavelet-LLH_gldm_HighGrayLevelEmphasis |
| wavelet-LLH | wavelet-LLH_gldm_LargeDependenceEmphasis |
| wavelet-LLH | wavelet-LLH_gldm_LargeDependenceHighGrayLevelEmphasis |
| wavelet-LLH | wavelet-LLH_gldm_LargeDependenceLowGrayLevelEmphasis |
| wavelet-LLH | wavelet-LLH_gldm_LowGrayLevelEmphasis |
| wavelet-LLH | wavelet-LLH_gldm_SmallDependenceEmphasis |
| wavelet-LLH | wavelet-LLH_gldm_SmallDependenceHighGrayLevelEmphasis |
| wavelet-LLH | wavelet-LLH_gldm_SmallDependenceLowGrayLevelEmphasis |
| wavelet-LLH | wavelet-LLH_glrlm_GrayLevelNonUniformityNormalized |
| wavelet-LLH | wavelet-LLH_glrlm_HighGrayLevelRunEmphasis |
| wavelet-LLH | wavelet-LLH_glrlm_LongRunEmphasis |
| wavelet-LLH | wavelet-LLH_glrlm_LongRunHighGrayLevelEmphasis |
| wavelet-LLH | wavelet-LLH_glrlm_LongRunLowGrayLevelEmphasis |
| wavelet-LLH | wavelet-LLH_glrlm_LowGrayLevelRunEmphasis |
| wavelet-LLH | wavelet-LLH_glrlm_RunEntropy |
| wavelet-LLH | wavelet-LLH_glrlm_RunLengthNonUniformityNormalized |
| wavelet-LLH | wavelet-LLH_glrlm_RunPercentage |
| wavelet-LLH | wavelet-LLH_glrlm_RunVariance |
| wavelet-LLH | wavelet-LLH_glrlm_ShortRunEmphasis |
| wavelet-LLH | wavelet-LLH_glrlm_ShortRunHighGrayLevelEmphasis |
| wavelet-LLH | wavelet-LLH_glrlm_ShortRunLowGrayLevelEmphasis |
| wavelet-LLH | wavelet-LLH_glszm_GrayLevelNonUniformityNormalized |
| wavelet-LLH | wavelet-LLH_glszm_GrayLevelVariance |
| wavelet-LLH | wavelet-LLH_glszm_HighGrayLevelZoneEmphasis |
| wavelet-LLH | wavelet-LLH_glszm_LargeAreaLowGrayLevelEmphasis |
| wavelet-LLH | wavelet-LLH_glszm_LowGrayLevelZoneEmphasis |
| wavelet-LLH | wavelet-LLH_glszm_SizeZoneNonUniformityNormalized |
| wavelet-LLH | wavelet-LLH_glszm_SmallAreaEmphasis |
| wavelet-LLH | wavelet-LLH_glszm_SmallAreaHighGrayLevelEmphasis |
| wavelet-LLH | wavelet-LLH_glszm_SmallAreaLowGrayLevelEmphasis |
| wavelet-LLH | wavelet-LLH_glszm_ZoneEntropy |
| wavelet-LLH | wavelet-LLH_glszm_ZonePercentage |
| wavelet-LLH | wavelet-LLH_ngtdm_Busyness |
| wavelet-LLH | wavelet-LLH_ngtdm_Coarseness |
| wavelet-LLH | wavelet-LLH_ngtdm_Complexity |
| wavelet-LLH | wavelet-LLH_ngtdm_Contrast |
| wavelet-LLH | wavelet-LLH_ngtdm_Strength |
| wavelet-LHL | wavelet-LHL_firstorder_10Percentile |
| wavelet-LHL | wavelet-LHL_firstorder_Kurtosis |
| wavelet-LHL | wavelet-LHL_firstorder_Maximum |
| wavelet-LHL | wavelet-LHL_firstorder_Mean |
| wavelet-LHL | wavelet-LHL_firstorder_Median |
| wavelet-LHL | wavelet-LHL_firstorder_Minimum |
| wavelet-LHL | wavelet-LHL_firstorder_Range |
| wavelet-LHL | wavelet-LHL_firstorder_Skewness |
| wavelet-LHL | wavelet-LHL_firstorder_Uniformity |
| wavelet-LHL | wavelet-LHL_glcm_Autocorrelation |
| wavelet-LHL | wavelet-LHL_glcm_ClusterProminence |
| wavelet-LHL | wavelet-LHL_glcm_ClusterShade |
| wavelet-LHL | wavelet-LHL_glcm_Correlation |
| wavelet-LHL | wavelet-LHL_glcm_Idmn |
| wavelet-LHL | wavelet-LHL_glcm_Idn |
| wavelet-LHL | wavelet-LHL_glcm_Imc1 |
| wavelet-LHL | wavelet-LHL_glcm_Imc2 |
| wavelet-LHL | wavelet-LHL_glcm_InverseVariance |
| wavelet-LHL | wavelet-LHL_glcm_JointAverage |
| wavelet-LHL | wavelet-LHL_glcm_JointEnergy |
| wavelet-LHL | wavelet-LHL_glcm_MCC |
| wavelet-LHL | wavelet-LHL_glcm_MaximumProbability |
| wavelet-LHL | wavelet-LHL_glcm_SumAverage |
| wavelet-LHL | wavelet-LHL_gldm_DependenceEntropy |
| wavelet-LHL | wavelet-LHL_gldm_DependenceNonUniformityNormalized |
| wavelet-LHL | wavelet-LHL_gldm_DependenceVariance |
| wavelet-LHL | wavelet-LHL_gldm_HighGrayLevelEmphasis |
| wavelet-LHL | wavelet-LHL_gldm_LargeDependenceEmphasis |
| wavelet-LHL | wavelet-LHL_gldm_LargeDependenceHighGrayLevelEmphasis |
| wavelet-LHL | wavelet-LHL_gldm_LargeDependenceLowGrayLevelEmphasis |
| wavelet-LHL | wavelet-LHL_gldm_LowGrayLevelEmphasis |
| wavelet-LHL | wavelet-LHL_gldm_SmallDependenceLowGrayLevelEmphasis |
| wavelet-LHL | wavelet-LHL_glrlm_GrayLevelNonUniformityNormalized |
| wavelet-LHL | wavelet-LHL_glrlm_HighGrayLevelRunEmphasis |
| wavelet-LHL | wavelet-LHL_glrlm_LongRunEmphasis |
| wavelet-LHL | wavelet-LHL_glrlm_LongRunHighGrayLevelEmphasis |
| wavelet-LHL | wavelet-LHL_glrlm_LongRunLowGrayLevelEmphasis |
| wavelet-LHL | wavelet-LHL_glrlm_LowGrayLevelRunEmphasis |
| wavelet-LHL | wavelet-LHL_glrlm_RunEntropy |
| wavelet-LHL | wavelet-LHL_glrlm_RunVariance |
| wavelet-LHL | wavelet-LHL_glrlm_ShortRunHighGrayLevelEmphasis |
| wavelet-LHL | wavelet-LHL_glrlm_ShortRunLowGrayLevelEmphasis |
| wavelet-LHL | wavelet-LHL_glszm_GrayLevelNonUniformityNormalized |
| wavelet-LHL | wavelet-LHL_glszm_GrayLevelVariance |
| wavelet-LHL | wavelet-LHL_glszm_HighGrayLevelZoneEmphasis |
| wavelet-LHL | wavelet-LHL_glszm_LargeAreaEmphasis |
| wavelet-LHL | wavelet-LHL_glszm_LargeAreaLowGrayLevelEmphasis |
| wavelet-LHL | wavelet-LHL_glszm_LowGrayLevelZoneEmphasis |
| wavelet-LHL | wavelet-LHL_glszm_SizeZoneNonUniformityNormalized |
| wavelet-LHL | wavelet-LHL_glszm_SmallAreaEmphasis |
| wavelet-LHL | wavelet-LHL_glszm_SmallAreaHighGrayLevelEmphasis |
| wavelet-LHL | wavelet-LHL_glszm_SmallAreaLowGrayLevelEmphasis |
| wavelet-LHL | wavelet-LHL_glszm_ZoneEntropy |
| wavelet-LHL | wavelet-LHL_glszm_ZoneVariance |
| wavelet-LHL | wavelet-LHL_ngtdm_Busyness |
| wavelet-LHL | wavelet-LHL_ngtdm_Complexity |
| wavelet-LHL | wavelet-LHL_ngtdm_Contrast |
| wavelet-LHL | wavelet-LHL_ngtdm_Strength |
| wavelet-LHH | wavelet-LHH_firstorder_10Percentile |
| wavelet-LHH | wavelet-LHH_firstorder_Kurtosis |
| wavelet-LHH | wavelet-LHH_firstorder_Maximum |
| wavelet-LHH | wavelet-LHH_firstorder_Mean |
| wavelet-LHH | wavelet-LHH_firstorder_Median |
| wavelet-LHH | wavelet-LHH_firstorder_Minimum |
| wavelet-LHH | wavelet-LHH_firstorder_Range |
| wavelet-LHH | wavelet-LHH_firstorder_Skewness |
| wavelet-LHH | wavelet-LHH_firstorder_Uniformity |
| wavelet-LHH | wavelet-LHH_glcm_Autocorrelation |
| wavelet-LHH | wavelet-LHH_glcm_ClusterProminence |
| wavelet-LHH | wavelet-LHH_glcm_ClusterShade |
| wavelet-LHH | wavelet-LHH_glcm_Correlation |
| wavelet-LHH | wavelet-LHH_glcm_Id |
| wavelet-LHH | wavelet-LHH_glcm_Idm |
| wavelet-LHH | wavelet-LHH_glcm_Idmn |
| wavelet-LHH | wavelet-LHH_glcm_Idn |
| wavelet-LHH | wavelet-LHH_glcm_Imc1 |
| wavelet-LHH | wavelet-LHH_glcm_Imc2 |
| wavelet-LHH | wavelet-LHH_glcm_InverseVariance |
| wavelet-LHH | wavelet-LHH_glcm_JointAverage |
| wavelet-LHH | wavelet-LHH_glcm_JointEnergy |
| wavelet-LHH | wavelet-LHH_glcm_MCC |
| wavelet-LHH | wavelet-LHH_glcm_MaximumProbability |
| wavelet-LHH | wavelet-LHH_glcm_SumAverage |
| wavelet-LHH | wavelet-LHH_gldm_DependenceEntropy |
| wavelet-LHH | wavelet-LHH_gldm_DependenceNonUniformityNormalized |
| wavelet-LHH | wavelet-LHH_gldm_DependenceVariance |
| wavelet-LHH | wavelet-LHH_gldm_HighGrayLevelEmphasis |
| wavelet-LHH | wavelet-LHH_gldm_LargeDependenceEmphasis |
| wavelet-LHH | wavelet-LHH_gldm_LargeDependenceHighGrayLevelEmphasis |
| wavelet-LHH | wavelet-LHH_gldm_LargeDependenceLowGrayLevelEmphasis |
| wavelet-LHH | wavelet-LHH_gldm_LowGrayLevelEmphasis |
| wavelet-LHH | wavelet-LHH_gldm_SmallDependenceHighGrayLevelEmphasis |
| wavelet-LHH | wavelet-LHH_gldm_SmallDependenceLowGrayLevelEmphasis |
| wavelet-LHH | wavelet-LHH_glrlm_GrayLevelNonUniformityNormalized |
| wavelet-LHH | wavelet-LHH_glrlm_HighGrayLevelRunEmphasis |
| wavelet-LHH | wavelet-LHH_glrlm_LongRunEmphasis |
| wavelet-LHH | wavelet-LHH_glrlm_LongRunHighGrayLevelEmphasis |
| wavelet-LHH | wavelet-LHH_glrlm_LongRunLowGrayLevelEmphasis |
| wavelet-LHH | wavelet-LHH_glrlm_LowGrayLevelRunEmphasis |
| wavelet-LHH | wavelet-LHH_glrlm_RunEntropy |
| wavelet-LHH | wavelet-LHH_glrlm_RunVariance |
| wavelet-LHH | wavelet-LHH_glrlm_ShortRunHighGrayLevelEmphasis |
| wavelet-LHH | wavelet-LHH_glrlm_ShortRunLowGrayLevelEmphasis |
| wavelet-LHH | wavelet-LHH_glszm_GrayLevelNonUniformityNormalized |
| wavelet-LHH | wavelet-LHH_glszm_GrayLevelVariance |
| wavelet-LHH | wavelet-LHH_glszm_HighGrayLevelZoneEmphasis |
| wavelet-LHH | wavelet-LHH_glszm_LargeAreaEmphasis |
| wavelet-LHH | wavelet-LHH_glszm_LargeAreaLowGrayLevelEmphasis |
| wavelet-LHH | wavelet-LHH_glszm_LowGrayLevelZoneEmphasis |
| wavelet-LHH | wavelet-LHH_glszm_SizeZoneNonUniformityNormalized |
| wavelet-LHH | wavelet-LHH_glszm_SmallAreaEmphasis |
| wavelet-LHH | wavelet-LHH_glszm_SmallAreaHighGrayLevelEmphasis |
| wavelet-LHH | wavelet-LHH_glszm_SmallAreaLowGrayLevelEmphasis |
| wavelet-LHH | wavelet-LHH_glszm_ZoneEntropy |
| wavelet-LHH | wavelet-LHH_glszm_ZoneVariance |
| wavelet-LHH | wavelet-LHH_ngtdm_Busyness |
| wavelet-LHH | wavelet-LHH_ngtdm_Complexity |
| wavelet-LHH | wavelet-LHH_ngtdm_Contrast |
| wavelet-LHH | wavelet-LHH_ngtdm_Strength |
| wavelet-HLL | wavelet-HLL_firstorder_10Percentile |
| wavelet-HLL | wavelet-HLL_firstorder_InterquartileRange |
| wavelet-HLL | wavelet-HLL_firstorder_Kurtosis |
| wavelet-HLL | wavelet-HLL_firstorder_Maximum |
| wavelet-HLL | wavelet-HLL_firstorder_Mean |
| wavelet-HLL | wavelet-HLL_firstorder_Median |
| wavelet-HLL | wavelet-HLL_firstorder_Minimum |
| wavelet-HLL | wavelet-HLL_firstorder_Range |
| wavelet-HLL | wavelet-HLL_firstorder_Skewness |
| wavelet-HLL | wavelet-HLL_firstorder_Uniformity |
| wavelet-HLL | wavelet-HLL_glcm_Autocorrelation |
| wavelet-HLL | wavelet-HLL_glcm_ClusterProminence |
| wavelet-HLL | wavelet-HLL_glcm_ClusterShade |
| wavelet-HLL | wavelet-HLL_glcm_Correlation |
| wavelet-HLL | wavelet-HLL_glcm_DifferenceVariance |
| wavelet-HLL | wavelet-HLL_glcm_Id |
| wavelet-HLL | wavelet-HLL_glcm_Idm |
| wavelet-HLL | wavelet-HLL_glcm_Idmn |
| wavelet-HLL | wavelet-HLL_glcm_Idn |
| wavelet-HLL | wavelet-HLL_glcm_Imc1 |
| wavelet-HLL | wavelet-HLL_glcm_Imc2 |
| wavelet-HLL | wavelet-HLL_glcm_InverseVariance |
| wavelet-HLL | wavelet-HLL_glcm_JointAverage |
| wavelet-HLL | wavelet-HLL_glcm_JointEnergy |
| wavelet-HLL | wavelet-HLL_glcm_MCC |
| wavelet-HLL | wavelet-HLL_glcm_MaximumProbability |
| wavelet-HLL | wavelet-HLL_glcm_SumAverage |
| wavelet-HLL | wavelet-HLL_gldm_DependenceEntropy |
| wavelet-HLL | wavelet-HLL_gldm_DependenceNonUniformityNormalized |
| wavelet-HLL | wavelet-HLL_gldm_DependenceVariance |
| wavelet-HLL | wavelet-HLL_gldm_HighGrayLevelEmphasis |
| wavelet-HLL | wavelet-HLL_gldm_LargeDependenceHighGrayLevelEmphasis |
| wavelet-HLL | wavelet-HLL_gldm_LargeDependenceLowGrayLevelEmphasis |
| wavelet-HLL | wavelet-HLL_gldm_LowGrayLevelEmphasis |
| wavelet-HLL | wavelet-HLL_gldm_SmallDependenceHighGrayLevelEmphasis |
| wavelet-HLL | wavelet-HLL_gldm_SmallDependenceLowGrayLevelEmphasis |
| wavelet-HLL | wavelet-HLL_glrlm_GrayLevelNonUniformityNormalized |
| wavelet-HLL | wavelet-HLL_glrlm_HighGrayLevelRunEmphasis |
| wavelet-HLL | wavelet-HLL_glrlm_LongRunEmphasis |
| wavelet-HLL | wavelet-HLL_glrlm_LongRunHighGrayLevelEmphasis |
| wavelet-HLL | wavelet-HLL_glrlm_LongRunLowGrayLevelEmphasis |
| wavelet-HLL | wavelet-HLL_glrlm_LowGrayLevelRunEmphasis |
| wavelet-HLL | wavelet-HLL_glrlm_RunEntropy |
| wavelet-HLL | wavelet-HLL_glrlm_RunVariance |
| wavelet-HLL | wavelet-HLL_glrlm_ShortRunHighGrayLevelEmphasis |
| wavelet-HLL | wavelet-HLL_glrlm_ShortRunLowGrayLevelEmphasis |
| wavelet-HLL | wavelet-HLL_glszm_GrayLevelNonUniformityNormalized |
| wavelet-HLL | wavelet-HLL_glszm_GrayLevelVariance |
| wavelet-HLL | wavelet-HLL_glszm_HighGrayLevelZoneEmphasis |
| wavelet-HLL | wavelet-HLL_glszm_LargeAreaLowGrayLevelEmphasis |
| wavelet-HLL | wavelet-HLL_glszm_LowGrayLevelZoneEmphasis |
| wavelet-HLL | wavelet-HLL_glszm_SizeZoneNonUniformityNormalized |
| wavelet-HLL | wavelet-HLL_glszm_SmallAreaEmphasis |
| wavelet-HLL | wavelet-HLL_glszm_SmallAreaHighGrayLevelEmphasis |
| wavelet-HLL | wavelet-HLL_glszm_SmallAreaLowGrayLevelEmphasis |
| wavelet-HLL | wavelet-HLL_glszm_ZoneEntropy |
| wavelet-HLL | wavelet-HLL_ngtdm_Busyness |
| wavelet-HLL | wavelet-HLL_ngtdm_Coarseness |
| wavelet-HLL | wavelet-HLL_ngtdm_Complexity |
| wavelet-HLL | wavelet-HLL_ngtdm_Contrast |
| wavelet-HLL | wavelet-HLL_ngtdm_Strength |
| wavelet-HLH | wavelet-HLH_firstorder_10Percentile |
| wavelet-HLH | wavelet-HLH_firstorder_Kurtosis |
| wavelet-HLH | wavelet-HLH_firstorder_Maximum |
| wavelet-HLH | wavelet-HLH_firstorder_Mean |
| wavelet-HLH | wavelet-HLH_firstorder_Median |
| wavelet-HLH | wavelet-HLH_firstorder_Minimum |
| wavelet-HLH | wavelet-HLH_firstorder_Range |
| wavelet-HLH | wavelet-HLH_firstorder_Skewness |
| wavelet-HLH | wavelet-HLH_firstorder_Uniformity |
| wavelet-HLH | wavelet-HLH_glcm_Autocorrelation |
| wavelet-HLH | wavelet-HLH_glcm_ClusterProminence |
| wavelet-HLH | wavelet-HLH_glcm_ClusterShade |
| wavelet-HLH | wavelet-HLH_glcm_ClusterTendency |
| wavelet-HLH | wavelet-HLH_glcm_Correlation |
| wavelet-HLH | wavelet-HLH_glcm_DifferenceVariance |
| wavelet-HLH | wavelet-HLH_glcm_Id |
| wavelet-HLH | wavelet-HLH_glcm_Idm |
| wavelet-HLH | wavelet-HLH_glcm_Idmn |
| wavelet-HLH | wavelet-HLH_glcm_Idn |
| wavelet-HLH | wavelet-HLH_glcm_Imc1 |
| wavelet-HLH | wavelet-HLH_glcm_Imc2 |
| wavelet-HLH | wavelet-HLH_glcm_InverseVariance |
| wavelet-HLH | wavelet-HLH_glcm_JointAverage |
| wavelet-HLH | wavelet-HLH_glcm_JointEnergy |
| wavelet-HLH | wavelet-HLH_glcm_MCC |
| wavelet-HLH | wavelet-HLH_glcm_MaximumProbability |
| wavelet-HLH | wavelet-HLH_glcm_SumAverage |
| wavelet-HLH | wavelet-HLH_glcm_SumSquares |
| wavelet-HLH | wavelet-HLH_gldm_DependenceEntropy |
| wavelet-HLH | wavelet-HLH_gldm_DependenceNonUniformityNormalized |
| wavelet-HLH | wavelet-HLH_gldm_DependenceVariance |
| wavelet-HLH | wavelet-HLH_gldm_GrayLevelVariance |
| wavelet-HLH | wavelet-HLH_gldm_HighGrayLevelEmphasis |
| wavelet-HLH | wavelet-HLH_gldm_LargeDependenceEmphasis |
| wavelet-HLH | wavelet-HLH_gldm_LargeDependenceHighGrayLevelEmphasis |
| wavelet-HLH | wavelet-HLH_gldm_LargeDependenceLowGrayLevelEmphasis |
| wavelet-HLH | wavelet-HLH_gldm_LowGrayLevelEmphasis |
| wavelet-HLH | wavelet-HLH_gldm_SmallDependenceHighGrayLevelEmphasis |
| wavelet-HLH | wavelet-HLH_gldm_SmallDependenceLowGrayLevelEmphasis |
| wavelet-HLH | wavelet-HLH_glrlm_GrayLevelNonUniformityNormalized |
| wavelet-HLH | wavelet-HLH_glrlm_HighGrayLevelRunEmphasis |
| wavelet-HLH | wavelet-HLH_glrlm_LongRunEmphasis |
| wavelet-HLH | wavelet-HLH_glrlm_LongRunHighGrayLevelEmphasis |
| wavelet-HLH | wavelet-HLH_glrlm_LongRunLowGrayLevelEmphasis |
| wavelet-HLH | wavelet-HLH_glrlm_LowGrayLevelRunEmphasis |
| wavelet-HLH | wavelet-HLH_glrlm_RunEntropy |
| wavelet-HLH | wavelet-HLH_glrlm_RunLengthNonUniformityNormalized |
| wavelet-HLH | wavelet-HLH_glrlm_RunPercentage |
| wavelet-HLH | wavelet-HLH_glrlm_RunVariance |
| wavelet-HLH | wavelet-HLH_glrlm_ShortRunEmphasis |
| wavelet-HLH | wavelet-HLH_glrlm_ShortRunHighGrayLevelEmphasis |
| wavelet-HLH | wavelet-HLH_glrlm_ShortRunLowGrayLevelEmphasis |
| wavelet-HLH | wavelet-HLH_glszm_GrayLevelNonUniformityNormalized |
| wavelet-HLH | wavelet-HLH_glszm_GrayLevelVariance |
| wavelet-HLH | wavelet-HLH_glszm_HighGrayLevelZoneEmphasis |
| wavelet-HLH | wavelet-HLH_glszm_LargeAreaEmphasis |
| wavelet-HLH | wavelet-HLH_glszm_LargeAreaHighGrayLevelEmphasis |
| wavelet-HLH | wavelet-HLH_glszm_LargeAreaLowGrayLevelEmphasis |
| wavelet-HLH | wavelet-HLH_glszm_LowGrayLevelZoneEmphasis |
| wavelet-HLH | wavelet-HLH_glszm_SizeZoneNonUniformityNormalized |
| wavelet-HLH | wavelet-HLH_glszm_SmallAreaEmphasis |
| wavelet-HLH | wavelet-HLH_glszm_SmallAreaHighGrayLevelEmphasis |
| wavelet-HLH | wavelet-HLH_glszm_SmallAreaLowGrayLevelEmphasis |
| wavelet-HLH | wavelet-HLH_glszm_ZoneEntropy |
| wavelet-HLH | wavelet-HLH_glszm_ZoneVariance |
| wavelet-HLH | wavelet-HLH_ngtdm_Busyness |
| wavelet-HLH | wavelet-HLH_ngtdm_Complexity |
| wavelet-HLH | wavelet-HLH_ngtdm_Contrast |
| wavelet-HLH | wavelet-HLH_ngtdm_Strength |
| wavelet-HHL | wavelet-HHL_firstorder_10Percentile |
| wavelet-HHL | wavelet-HHL_firstorder_Kurtosis |
| wavelet-HHL | wavelet-HHL_firstorder_Maximum |
| wavelet-HHL | wavelet-HHL_firstorder_Mean |
| wavelet-HHL | wavelet-HHL_firstorder_Median |
| wavelet-HHL | wavelet-HHL_firstorder_Minimum |
| wavelet-HHL | wavelet-HHL_firstorder_Skewness |
| wavelet-HHL | wavelet-HHL_firstorder_Uniformity |
| wavelet-HHL | wavelet-HHL_glcm_Autocorrelation |
| wavelet-HHL | wavelet-HHL_glcm_ClusterProminence |
| wavelet-HHL | wavelet-HHL_glcm_ClusterShade |
| wavelet-HHL | wavelet-HHL_glcm_Correlation |
| wavelet-HHL | wavelet-HHL_glcm_Id |
| wavelet-HHL | wavelet-HHL_glcm_Idm |
| wavelet-HHL | wavelet-HHL_glcm_Idmn |
| wavelet-HHL | wavelet-HHL_glcm_Idn |
| wavelet-HHL | wavelet-HHL_glcm_Imc1 |
| wavelet-HHL | wavelet-HHL_glcm_Imc2 |
| wavelet-HHL | wavelet-HHL_glcm_InverseVariance |
| wavelet-HHL | wavelet-HHL_glcm_JointAverage |
| wavelet-HHL | wavelet-HHL_glcm_JointEnergy |
| wavelet-HHL | wavelet-HHL_glcm_MCC |
| wavelet-HHL | wavelet-HHL_glcm_MaximumProbability |
| wavelet-HHL | wavelet-HHL_glcm_SumAverage |
| wavelet-HHL | wavelet-HHL_gldm_DependenceNonUniformityNormalized |
| wavelet-HHL | wavelet-HHL_gldm_DependenceVariance |
| wavelet-HHL | wavelet-HHL_gldm_HighGrayLevelEmphasis |
| wavelet-HHL | wavelet-HHL_gldm_LargeDependenceEmphasis |
| wavelet-HHL | wavelet-HHL_gldm_LargeDependenceHighGrayLevelEmphasis |
| wavelet-HHL | wavelet-HHL_gldm_LargeDependenceLowGrayLevelEmphasis |
| wavelet-HHL | wavelet-HHL_gldm_LowGrayLevelEmphasis |
| wavelet-HHL | wavelet-HHL_gldm_SmallDependenceLowGrayLevelEmphasis |
| wavelet-HHL | wavelet-HHL_glrlm_GrayLevelNonUniformityNormalized |
| wavelet-HHL | wavelet-HHL_glrlm_HighGrayLevelRunEmphasis |
| wavelet-HHL | wavelet-HHL_glrlm_LongRunEmphasis |
| wavelet-HHL | wavelet-HHL_glrlm_LongRunHighGrayLevelEmphasis |
| wavelet-HHL | wavelet-HHL_glrlm_LongRunLowGrayLevelEmphasis |
| wavelet-HHL | wavelet-HHL_glrlm_LowGrayLevelRunEmphasis |
| wavelet-HHL | wavelet-HHL_glrlm_RunEntropy |
| wavelet-HHL | wavelet-HHL_glrlm_RunVariance |
| wavelet-HHL | wavelet-HHL_glrlm_ShortRunHighGrayLevelEmphasis |
| wavelet-HHL | wavelet-HHL_glrlm_ShortRunLowGrayLevelEmphasis |
| wavelet-HHL | wavelet-HHL_glszm_GrayLevelNonUniformityNormalized |
| wavelet-HHL | wavelet-HHL_glszm_HighGrayLevelZoneEmphasis |
| wavelet-HHL | wavelet-HHL_glszm_LargeAreaEmphasis |
| wavelet-HHL | wavelet-HHL_glszm_LargeAreaLowGrayLevelEmphasis |
| wavelet-HHL | wavelet-HHL_glszm_LowGrayLevelZoneEmphasis |
| wavelet-HHL | wavelet-HHL_glszm_SizeZoneNonUniformityNormalized |
| wavelet-HHL | wavelet-HHL_glszm_SmallAreaEmphasis |
| wavelet-HHL | wavelet-HHL_glszm_SmallAreaHighGrayLevelEmphasis |
| wavelet-HHL | wavelet-HHL_glszm_SmallAreaLowGrayLevelEmphasis |
| wavelet-HHL | wavelet-HHL_glszm_ZoneEntropy |
| wavelet-HHL | wavelet-HHL_glszm_ZoneVariance |
| wavelet-HHL | wavelet-HHL_ngtdm_Busyness |
| wavelet-HHL | wavelet-HHL_ngtdm_Complexity |
| wavelet-HHL | wavelet-HHL_ngtdm_Contrast |
| wavelet-HHL | wavelet-HHL_ngtdm_Strength |
| wavelet-HHH | wavelet-HHH_firstorder_10Percentile |
| wavelet-HHH | wavelet-HHH_firstorder_Entropy |
| wavelet-HHH | wavelet-HHH_firstorder_Kurtosis |
| wavelet-HHH | wavelet-HHH_firstorder_Maximum |
| wavelet-HHH | wavelet-HHH_firstorder_Mean |
| wavelet-HHH | wavelet-HHH_firstorder_Median |
| wavelet-HHH | wavelet-HHH_firstorder_Minimum |
| wavelet-HHH | wavelet-HHH_firstorder_Skewness |
| wavelet-HHH | wavelet-HHH_firstorder_Uniformity |
| wavelet-HHH | wavelet-HHH_glcm_Autocorrelation |
| wavelet-HHH | wavelet-HHH_glcm_ClusterProminence |
| wavelet-HHH | wavelet-HHH_glcm_ClusterShade |
| wavelet-HHH | wavelet-HHH_glcm_ClusterTendency |
| wavelet-HHH | wavelet-HHH_glcm_Contrast |
| wavelet-HHH | wavelet-HHH_glcm_Correlation |
| wavelet-HHH | wavelet-HHH_glcm_DifferenceAverage |
| wavelet-HHH | wavelet-HHH_glcm_DifferenceEntropy |
| wavelet-HHH | wavelet-HHH_glcm_DifferenceVariance |
| wavelet-HHH | wavelet-HHH_glcm_Id |
| wavelet-HHH | wavelet-HHH_glcm_Idm |
| wavelet-HHH | wavelet-HHH_glcm_Idmn |
| wavelet-HHH | wavelet-HHH_glcm_Idn |
| wavelet-HHH | wavelet-HHH_glcm_Imc1 |
| wavelet-HHH | wavelet-HHH_glcm_Imc2 |
| wavelet-HHH | wavelet-HHH_glcm_InverseVariance |
| wavelet-HHH | wavelet-HHH_glcm_JointAverage |
| wavelet-HHH | wavelet-HHH_glcm_JointEnergy |
| wavelet-HHH | wavelet-HHH_glcm_JointEntropy |
| wavelet-HHH | wavelet-HHH_glcm_MCC |
| wavelet-HHH | wavelet-HHH_glcm_MaximumProbability |
| wavelet-HHH | wavelet-HHH_glcm_SumAverage |
| wavelet-HHH | wavelet-HHH_glcm_SumEntropy |
| wavelet-HHH | wavelet-HHH_glcm_SumSquares |
| wavelet-HHH | wavelet-HHH_gldm_DependenceEntropy |
| wavelet-HHH | wavelet-HHH_gldm_DependenceNonUniformityNormalized |
| wavelet-HHH | wavelet-HHH_gldm_DependenceVariance |
| wavelet-HHH | wavelet-HHH_gldm_GrayLevelVariance |
| wavelet-HHH | wavelet-HHH_gldm_HighGrayLevelEmphasis |
| wavelet-HHH | wavelet-HHH_gldm_LargeDependenceEmphasis |
| wavelet-HHH | wavelet-HHH_gldm_LargeDependenceHighGrayLevelEmphasis |
| wavelet-HHH | wavelet-HHH_gldm_LargeDependenceLowGrayLevelEmphasis |
| wavelet-HHH | wavelet-HHH_gldm_LowGrayLevelEmphasis |
| wavelet-HHH | wavelet-HHH_gldm_SmallDependenceEmphasis |
| wavelet-HHH | wavelet-HHH_gldm_SmallDependenceLowGrayLevelEmphasis |
| wavelet-HHH | wavelet-HHH_glrlm_GrayLevelNonUniformityNormalized |
| wavelet-HHH | wavelet-HHH_glrlm_GrayLevelVariance |
| wavelet-HHH | wavelet-HHH_glrlm_HighGrayLevelRunEmphasis |
| wavelet-HHH | wavelet-HHH_glrlm_LongRunEmphasis |
| wavelet-HHH | wavelet-HHH_glrlm_LongRunHighGrayLevelEmphasis |
| wavelet-HHH | wavelet-HHH_glrlm_LongRunLowGrayLevelEmphasis |
| wavelet-HHH | wavelet-HHH_glrlm_LowGrayLevelRunEmphasis |
| wavelet-HHH | wavelet-HHH_glrlm_RunEntropy |
| wavelet-HHH | wavelet-HHH_glrlm_RunLengthNonUniformityNormalized |
| wavelet-HHH | wavelet-HHH_glrlm_RunPercentage |
| wavelet-HHH | wavelet-HHH_glrlm_RunVariance |
| wavelet-HHH | wavelet-HHH_glrlm_ShortRunEmphasis |
| wavelet-HHH | wavelet-HHH_glrlm_ShortRunHighGrayLevelEmphasis |
| wavelet-HHH | wavelet-HHH_glrlm_ShortRunLowGrayLevelEmphasis |
| wavelet-HHH | wavelet-HHH_glszm_GrayLevelNonUniformity |
| wavelet-HHH | wavelet-HHH_glszm_GrayLevelNonUniformityNormalized |
| wavelet-HHH | wavelet-HHH_glszm_GrayLevelVariance |
| wavelet-HHH | wavelet-HHH_glszm_HighGrayLevelZoneEmphasis |
| wavelet-HHH | wavelet-HHH_glszm_LargeAreaEmphasis |
| wavelet-HHH | wavelet-HHH_glszm_LargeAreaHighGrayLevelEmphasis |
| wavelet-HHH | wavelet-HHH_glszm_LargeAreaLowGrayLevelEmphasis |
| wavelet-HHH | wavelet-HHH_glszm_LowGrayLevelZoneEmphasis |
| wavelet-HHH | wavelet-HHH_glszm_SizeZoneNonUniformity |
| wavelet-HHH | wavelet-HHH_glszm_SizeZoneNonUniformityNormalized |
| wavelet-HHH | wavelet-HHH_glszm_SmallAreaEmphasis |
| wavelet-HHH | wavelet-HHH_glszm_SmallAreaHighGrayLevelEmphasis |
| wavelet-HHH | wavelet-HHH_glszm_SmallAreaLowGrayLevelEmphasis |
| wavelet-HHH | wavelet-HHH_glszm_ZoneEntropy |
| wavelet-HHH | wavelet-HHH_glszm_ZonePercentage |
| wavelet-HHH | wavelet-HHH_glszm_ZoneVariance |
| wavelet-HHH | wavelet-HHH_ngtdm_Busyness |
| wavelet-HHH | wavelet-HHH_ngtdm_Complexity |
| wavelet-HHH | wavelet-HHH_ngtdm_Contrast |
| wavelet-HHH | wavelet-HHH_ngtdm_Strength |
| wavelet-LLL | wavelet-LLL_firstorder_10Percentile |
| wavelet-LLL | wavelet-LLL_firstorder_90Percentile |
| wavelet-LLL | wavelet-LLL_firstorder_Entropy |
| wavelet-LLL | wavelet-LLL_firstorder_InterquartileRange |
| wavelet-LLL | wavelet-LLL_firstorder_Kurtosis |
| wavelet-LLL | wavelet-LLL_firstorder_Maximum |
| wavelet-LLL | wavelet-LLL_firstorder_Minimum |
| wavelet-LLL | wavelet-LLL_firstorder_Range |
| wavelet-LLL | wavelet-LLL_firstorder_Skewness |
| wavelet-LLL | wavelet-LLL_firstorder_Uniformity |
| wavelet-LLL | wavelet-LLL_glcm_Autocorrelation |
| wavelet-LLL | wavelet-LLL_glcm_ClusterProminence |
| wavelet-LLL | wavelet-LLL_glcm_ClusterShade |
| wavelet-LLL | wavelet-LLL_glcm_Correlation |
| wavelet-LLL | wavelet-LLL_glcm_Id |
| wavelet-LLL | wavelet-LLL_glcm_Idm |
| wavelet-LLL | wavelet-LLL_glcm_Idmn |
| wavelet-LLL | wavelet-LLL_glcm_Idn |
| wavelet-LLL | wavelet-LLL_glcm_Imc1 |
| wavelet-LLL | wavelet-LLL_glcm_Imc2 |
| wavelet-LLL | wavelet-LLL_glcm_InverseVariance |
| wavelet-LLL | wavelet-LLL_glcm_JointAverage |
| wavelet-LLL | wavelet-LLL_glcm_JointEnergy |
| wavelet-LLL | wavelet-LLL_glcm_JointEntropy |
| wavelet-LLL | wavelet-LLL_glcm_MCC |
| wavelet-LLL | wavelet-LLL_glcm_MaximumProbability |
| wavelet-LLL | wavelet-LLL_glcm_SumAverage |
| wavelet-LLL | wavelet-LLL_glcm_SumEntropy |
| wavelet-LLL | wavelet-LLL_gldm_DependenceEntropy |
| wavelet-LLL | wavelet-LLL_gldm_DependenceNonUniformityNormalized |
| wavelet-LLL | wavelet-LLL_gldm_DependenceVariance |
| wavelet-LLL | wavelet-LLL_gldm_HighGrayLevelEmphasis |
| wavelet-LLL | wavelet-LLL_gldm_LargeDependenceEmphasis |
| wavelet-LLL | wavelet-LLL_gldm_LargeDependenceHighGrayLevelEmphasis |
| wavelet-LLL | wavelet-LLL_gldm_LargeDependenceLowGrayLevelEmphasis |
| wavelet-LLL | wavelet-LLL_gldm_LowGrayLevelEmphasis |
| wavelet-LLL | wavelet-LLL_gldm_SmallDependenceHighGrayLevelEmphasis |
| wavelet-LLL | wavelet-LLL_gldm_SmallDependenceLowGrayLevelEmphasis |
| wavelet-LLL | wavelet-LLL_glrlm_GrayLevelNonUniformityNormalized |
| wavelet-LLL | wavelet-LLL_glrlm_HighGrayLevelRunEmphasis |
| wavelet-LLL | wavelet-LLL_glrlm_LongRunEmphasis |
| wavelet-LLL | wavelet-LLL_glrlm_LongRunHighGrayLevelEmphasis |
| wavelet-LLL | wavelet-LLL_glrlm_LongRunLowGrayLevelEmphasis |
| wavelet-LLL | wavelet-LLL_glrlm_LowGrayLevelRunEmphasis |
| wavelet-LLL | wavelet-LLL_glrlm_RunEntropy |
| wavelet-LLL | wavelet-LLL_glrlm_RunLengthNonUniformityNormalized |
| wavelet-LLL | wavelet-LLL_glrlm_RunPercentage |
| wavelet-LLL | wavelet-LLL_glrlm_RunVariance |
| wavelet-LLL | wavelet-LLL_glrlm_ShortRunEmphasis |
| wavelet-LLL | wavelet-LLL_glrlm_ShortRunHighGrayLevelEmphasis |
| wavelet-LLL | wavelet-LLL_glrlm_ShortRunLowGrayLevelEmphasis |
| wavelet-LLL | wavelet-LLL_glszm_GrayLevelNonUniformityNormalized |
| wavelet-LLL | wavelet-LLL_glszm_GrayLevelVariance |
| wavelet-LLL | wavelet-LLL_glszm_HighGrayLevelZoneEmphasis |
| wavelet-LLL | wavelet-LLL_glszm_LargeAreaLowGrayLevelEmphasis |
| wavelet-LLL | wavelet-LLL_glszm_LowGrayLevelZoneEmphasis |
| wavelet-LLL | wavelet-LLL_glszm_SizeZoneNonUniformityNormalized |
| wavelet-LLL | wavelet-LLL_glszm_SmallAreaEmphasis |
| wavelet-LLL | wavelet-LLL_glszm_SmallAreaHighGrayLevelEmphasis |
| wavelet-LLL | wavelet-LLL_glszm_SmallAreaLowGrayLevelEmphasis |
| wavelet-LLL | wavelet-LLL_glszm_ZoneEntropy |
| wavelet-LLL | wavelet-LLL_glszm_ZonePercentage |
| wavelet-LLL | wavelet-LLL_ngtdm_Complexity |
| wavelet-LLL | wavelet-LLL_ngtdm_Contrast |
| wavelet-LLL | wavelet-LLL_ngtdm_Strength |
| square | square_firstorder_10Percentile |
| square | square_firstorder_90Percentile |
| square | square_firstorder_Entropy |
| square | square_firstorder_InterquartileRange |
| square | square_firstorder_Kurtosis |
| square | square_firstorder_Maximum |
| square | square_firstorder_Minimum |
| square | square_firstorder_Range |
| square | square_firstorder_Skewness |
| square | square_firstorder_Uniformity |
| square | square_glcm_ClusterProminence |
| square | square_glcm_ClusterShade |
| square | square_glcm_Correlation |
| square | square_glcm_Id |
| square | square_glcm_Idm |
| square | square_glcm_Idmn |
| square | square_glcm_Idn |
| square | square_glcm_Imc1 |
| square | square_glcm_Imc2 |
| square | square_glcm_InverseVariance |
| square | square_glcm_JointEnergy |
| square | square_glcm_JointEntropy |
| square | square_glcm_MCC |
| square | square_glcm_MaximumProbability |
| square | square_glcm_SumEntropy |
| square | square_gldm_DependenceEntropy |
| square | square_gldm_DependenceNonUniformityNormalized |
| square | square_gldm_DependenceVariance |
| square | square_gldm_LargeDependenceEmphasis |
| square | square_gldm_LargeDependenceHighGrayLevelEmphasis |
| square | square_gldm_LargeDependenceLowGrayLevelEmphasis |
| square | square_gldm_LowGrayLevelEmphasis |
| square | square_gldm_SmallDependenceEmphasis |
| square | square_gldm_SmallDependenceHighGrayLevelEmphasis |
| square | square_gldm_SmallDependenceLowGrayLevelEmphasis |
| square | square_glrlm_GrayLevelNonUniformityNormalized |
| square | square_glrlm_GrayLevelVariance |
| square | square_glrlm_LongRunEmphasis |
| square | square_glrlm_LongRunHighGrayLevelEmphasis |
| square | square_glrlm_LongRunLowGrayLevelEmphasis |
| square | square_glrlm_LowGrayLevelRunEmphasis |
| square | square_glrlm_RunEntropy |
| square | square_glrlm_RunLengthNonUniformityNormalized |
| square | square_glrlm_RunPercentage |
| square | square_glrlm_RunVariance |
| square | square_glrlm_ShortRunEmphasis |
| square | square_glrlm_ShortRunHighGrayLevelEmphasis |
| square | square_glrlm_ShortRunLowGrayLevelEmphasis |
| square | square_glszm_GrayLevelNonUniformityNormalized |
| square | square_glszm_GrayLevelVariance |
| square | square_glszm_HighGrayLevelZoneEmphasis |
| square | square_glszm_LargeAreaLowGrayLevelEmphasis |
| square | square_glszm_LowGrayLevelZoneEmphasis |
| square | square_glszm_SizeZoneNonUniformityNormalized |
| square | square_glszm_SmallAreaEmphasis |
| square | square_glszm_SmallAreaHighGrayLevelEmphasis |
| square | square_glszm_SmallAreaLowGrayLevelEmphasis |
| square | square_glszm_ZoneEntropy |
| square | square_glszm_ZonePercentage |
| square | square_ngtdm_Busyness |
| square | square_ngtdm_Complexity |
| square | square_ngtdm_Contrast |
| square | square_ngtdm_Strength |
| squareroot | squareroot_firstorder_10Percentile |
| squareroot | squareroot_firstorder_90Percentile |
| squareroot | squareroot_firstorder_Entropy |
| squareroot | squareroot_firstorder_Kurtosis |
| squareroot | squareroot_firstorder_Maximum |
| squareroot | squareroot_firstorder_Minimum |
| squareroot | squareroot_firstorder_Range |
| squareroot | squareroot_firstorder_Skewness |
| squareroot | squareroot_firstorder_Uniformity |
| squareroot | squareroot_glcm_Autocorrelation |
| squareroot | squareroot_glcm_ClusterProminence |
| squareroot | squareroot_glcm_ClusterShade |
| squareroot | squareroot_glcm_Correlation |
| squareroot | squareroot_glcm_DifferenceAverage |
| squareroot | squareroot_glcm_Id |
| squareroot | squareroot_glcm_Idm |
| squareroot | squareroot_glcm_Idmn |
| squareroot | squareroot_glcm_Idn |
| squareroot | squareroot_glcm_Imc1 |
| squareroot | squareroot_glcm_Imc2 |
| squareroot | squareroot_glcm_InverseVariance |
| squareroot | squareroot_glcm_JointAverage |
| squareroot | squareroot_glcm_JointEnergy |
| squareroot | squareroot_glcm_JointEntropy |
| squareroot | squareroot_glcm_MCC |
| squareroot | squareroot_glcm_MaximumProbability |
| squareroot | squareroot_glcm_SumAverage |
| squareroot | squareroot_glcm_SumEntropy |
| squareroot | squareroot_gldm_DependenceEntropy |
| squareroot | squareroot_gldm_DependenceNonUniformityNormalized |
| squareroot | squareroot_gldm_DependenceVariance |
| squareroot | squareroot_gldm_HighGrayLevelEmphasis |
| squareroot | squareroot_gldm_LargeDependenceEmphasis |
| squareroot | squareroot_gldm_LargeDependenceHighGrayLevelEmphasis |
| squareroot | squareroot_gldm_LargeDependenceLowGrayLevelEmphasis |
| squareroot | squareroot_gldm_LowGrayLevelEmphasis |
| squareroot | squareroot_gldm_SmallDependenceHighGrayLevelEmphasis |
| squareroot | squareroot_gldm_SmallDependenceLowGrayLevelEmphasis |
| squareroot | squareroot_glrlm_GrayLevelNonUniformityNormalized |
| squareroot | squareroot_glrlm_GrayLevelVariance |
| squareroot | squareroot_glrlm_HighGrayLevelRunEmphasis |
| squareroot | squareroot_glrlm_LongRunEmphasis |
| squareroot | squareroot_glrlm_LongRunLowGrayLevelEmphasis |
| squareroot | squareroot_glrlm_LowGrayLevelRunEmphasis |
| squareroot | squareroot_glrlm_RunEntropy |
| squareroot | squareroot_glrlm_RunPercentage |
| squareroot | squareroot_glrlm_ShortRunEmphasis |
| squareroot | squareroot_glrlm_ShortRunHighGrayLevelEmphasis |
| squareroot | squareroot_glrlm_ShortRunLowGrayLevelEmphasis |
| squareroot | squareroot_glszm_GrayLevelNonUniformityNormalized |
| squareroot | squareroot_glszm_GrayLevelVariance |
| squareroot | squareroot_glszm_HighGrayLevelZoneEmphasis |
| squareroot | squareroot_glszm_LargeAreaLowGrayLevelEmphasis |
| squareroot | squareroot_glszm_LowGrayLevelZoneEmphasis |
| squareroot | squareroot_glszm_SizeZoneNonUniformityNormalized |
| squareroot | squareroot_glszm_SmallAreaEmphasis |
| squareroot | squareroot_glszm_SmallAreaHighGrayLevelEmphasis |
| squareroot | squareroot_glszm_SmallAreaLowGrayLevelEmphasis |
| squareroot | squareroot_glszm_ZoneEntropy |
| squareroot | squareroot_glszm_ZonePercentage |
| squareroot | squareroot_ngtdm_Busyness |
| squareroot | squareroot_ngtdm_Coarseness |
| squareroot | squareroot_ngtdm_Complexity |
| squareroot | squareroot_ngtdm_Contrast |
| squareroot | squareroot_ngtdm_Strength |
| logarithm | logarithm_firstorder_10Percentile |
| logarithm | logarithm_firstorder_90Percentile |
| logarithm | logarithm_firstorder_Entropy |
| logarithm | logarithm_firstorder_InterquartileRange |
| logarithm | logarithm_firstorder_Kurtosis |
| logarithm | logarithm_firstorder_Maximum |
| logarithm | logarithm_firstorder_Minimum |
| logarithm | logarithm_firstorder_Range |
| logarithm | logarithm_firstorder_Skewness |
| logarithm | logarithm_firstorder_Uniformity |
| logarithm | logarithm_glcm_Autocorrelation |
| logarithm | logarithm_glcm_ClusterProminence |
| logarithm | logarithm_glcm_ClusterShade |
| logarithm | logarithm_glcm_ClusterTendency |
| logarithm | logarithm_glcm_Contrast |
| logarithm | logarithm_glcm_Correlation |
| logarithm | logarithm_glcm_DifferenceAverage |
| logarithm | logarithm_glcm_DifferenceEntropy |
| logarithm | logarithm_glcm_Id |
| logarithm | logarithm_glcm_Idm |
| logarithm | logarithm_glcm_Idmn |
| logarithm | logarithm_glcm_Idn |
| logarithm | logarithm_glcm_Imc1 |
| logarithm | logarithm_glcm_Imc2 |
| logarithm | logarithm_glcm_InverseVariance |
| logarithm | logarithm_glcm_JointAverage |
| logarithm | logarithm_glcm_JointEnergy |
| logarithm | logarithm_glcm_JointEntropy |
| logarithm | logarithm_glcm_MCC |
| logarithm | logarithm_glcm_MaximumProbability |
| logarithm | logarithm_glcm_SumAverage |
| logarithm | logarithm_glcm_SumEntropy |
| logarithm | logarithm_glcm_SumSquares |
| logarithm | logarithm_gldm_DependenceEntropy |
| logarithm | logarithm_gldm_DependenceNonUniformityNormalized |
| logarithm | logarithm_gldm_DependenceVariance |
| logarithm | logarithm_gldm_HighGrayLevelEmphasis |
| logarithm | logarithm_gldm_LargeDependenceEmphasis |
| logarithm | logarithm_gldm_LargeDependenceHighGrayLevelEmphasis |
| logarithm | logarithm_gldm_LargeDependenceLowGrayLevelEmphasis |
| logarithm | logarithm_gldm_LowGrayLevelEmphasis |
| logarithm | logarithm_gldm_SmallDependenceEmphasis |
| logarithm | logarithm_gldm_SmallDependenceHighGrayLevelEmphasis |
| logarithm | logarithm_gldm_SmallDependenceLowGrayLevelEmphasis |
| logarithm | logarithm_glrlm_GrayLevelNonUniformityNormalized |
| logarithm | logarithm_glrlm_GrayLevelVariance |
| logarithm | logarithm_glrlm_HighGrayLevelRunEmphasis |
| logarithm | logarithm_glrlm_LongRunEmphasis |
| logarithm | logarithm_glrlm_LongRunHighGrayLevelEmphasis |
| logarithm | logarithm_glrlm_LongRunLowGrayLevelEmphasis |
| logarithm | logarithm_glrlm_LowGrayLevelRunEmphasis |
| logarithm | logarithm_glrlm_RunEntropy |
| logarithm | logarithm_glrlm_RunLengthNonUniformityNormalized |
| logarithm | logarithm_glrlm_RunPercentage |
| logarithm | logarithm_glrlm_RunVariance |
| logarithm | logarithm_glrlm_ShortRunEmphasis |
| logarithm | logarithm_glrlm_ShortRunHighGrayLevelEmphasis |
| logarithm | logarithm_glrlm_ShortRunLowGrayLevelEmphasis |
| logarithm | logarithm_glszm_GrayLevelNonUniformityNormalized |
| logarithm | logarithm_glszm_GrayLevelVariance |
| logarithm | logarithm_glszm_HighGrayLevelZoneEmphasis |
| logarithm | logarithm_glszm_LargeAreaEmphasis |
| logarithm | logarithm_glszm_LargeAreaLowGrayLevelEmphasis |
| logarithm | logarithm_glszm_LowGrayLevelZoneEmphasis |
| logarithm | logarithm_glszm_SizeZoneNonUniformityNormalized |
| logarithm | logarithm_glszm_SmallAreaEmphasis |
| logarithm | logarithm_glszm_SmallAreaHighGrayLevelEmphasis |
| logarithm | logarithm_glszm_SmallAreaLowGrayLevelEmphasis |
| logarithm | logarithm_glszm_ZoneEntropy |
| logarithm | logarithm_glszm_ZonePercentage |
| logarithm | logarithm_glszm_ZoneVariance |
| logarithm | logarithm_ngtdm_Busyness |
| logarithm | logarithm_ngtdm_Coarseness |
| logarithm | logarithm_ngtdm_Complexity |
| logarithm | logarithm_ngtdm_Contrast |
| logarithm | logarithm_ngtdm_Strength |
| exponential | exponential_firstorder_10Percentile |
| exponential | exponential_firstorder_90Percentile |
| exponential | exponential_firstorder_Entropy |
| exponential | exponential_firstorder_InterquartileRange |
| exponential | exponential_firstorder_Kurtosis |
| exponential | exponential_firstorder_Maximum |
| exponential | exponential_firstorder_MeanAbsoluteDeviation |
| exponential | exponential_firstorder_Minimum |
| exponential | exponential_firstorder_Range |
| exponential | exponential_firstorder_RobustMeanAbsoluteDeviation |
| exponential | exponential_firstorder_Skewness |
| exponential | exponential_firstorder_Uniformity |
| exponential | exponential_firstorder_Variance |
| exponential | exponential_glcm_ClusterProminence |
| exponential | exponential_glcm_ClusterShade |
| exponential | exponential_glcm_ClusterTendency |
| exponential | exponential_glcm_Contrast |
| exponential | exponential_glcm_Correlation |
| exponential | exponential_glcm_DifferenceAverage |
| exponential | exponential_glcm_DifferenceEntropy |
| exponential | exponential_glcm_DifferenceVariance |
| exponential | exponential_glcm_Id |
| exponential | exponential_glcm_Idm |
| exponential | exponential_glcm_Idmn |
| exponential | exponential_glcm_Idn |
| exponential | exponential_glcm_Imc1 |
| exponential | exponential_glcm_Imc2 |
| exponential | exponential_glcm_InverseVariance |
| exponential | exponential_glcm_JointEnergy |
| exponential | exponential_glcm_JointEntropy |
| exponential | exponential_glcm_MCC |
| exponential | exponential_glcm_MaximumProbability |
| exponential | exponential_glcm_SumEntropy |
| exponential | exponential_glcm_SumSquares |
| exponential | exponential_gldm_DependenceEntropy |
| exponential | exponential_gldm_DependenceNonUniformityNormalized |
| exponential | exponential_gldm_DependenceVariance |
| exponential | exponential_gldm_GrayLevelVariance |
| exponential | exponential_gldm_HighGrayLevelEmphasis |
| exponential | exponential_gldm_LargeDependenceEmphasis |
| exponential | exponential_gldm_LargeDependenceHighGrayLevelEmphasis |
| exponential | exponential_gldm_LargeDependenceLowGrayLevelEmphasis |
| exponential | exponential_gldm_LowGrayLevelEmphasis |
| exponential | exponential_gldm_SmallDependenceEmphasis |
| exponential | exponential_gldm_SmallDependenceHighGrayLevelEmphasis |
| exponential | exponential_gldm_SmallDependenceLowGrayLevelEmphasis |
| exponential | exponential_glrlm_GrayLevelNonUniformityNormalized |
| exponential | exponential_glrlm_GrayLevelVariance |
| exponential | exponential_glrlm_HighGrayLevelRunEmphasis |
| exponential | exponential_glrlm_LongRunEmphasis |
| exponential | exponential_glrlm_LongRunHighGrayLevelEmphasis |
| exponential | exponential_glrlm_LongRunLowGrayLevelEmphasis |
| exponential | exponential_glrlm_LowGrayLevelRunEmphasis |
| exponential | exponential_glrlm_RunEntropy |
| exponential | exponential_glrlm_RunLengthNonUniformityNormalized |
| exponential | exponential_glrlm_RunPercentage |
| exponential | exponential_glrlm_RunVariance |
| exponential | exponential_glrlm_ShortRunEmphasis |
| exponential | exponential_glrlm_ShortRunHighGrayLevelEmphasis |
| exponential | exponential_glrlm_ShortRunLowGrayLevelEmphasis |
| exponential | exponential_glszm_GrayLevelNonUniformityNormalized |
| exponential | exponential_glszm_GrayLevelVariance |
| exponential | exponential_glszm_HighGrayLevelZoneEmphasis |
| exponential | exponential_glszm_LargeAreaEmphasis |
| exponential | exponential_glszm_LargeAreaHighGrayLevelEmphasis |
| exponential | exponential_glszm_LargeAreaLowGrayLevelEmphasis |
| exponential | exponential_glszm_LowGrayLevelZoneEmphasis |
| exponential | exponential_glszm_SizeZoneNonUniformityNormalized |
| exponential | exponential_glszm_SmallAreaEmphasis |
| exponential | exponential_glszm_SmallAreaHighGrayLevelEmphasis |
| exponential | exponential_glszm_SmallAreaLowGrayLevelEmphasis |
| exponential | exponential_glszm_ZoneEntropy |
| exponential | exponential_glszm_ZonePercentage |
| exponential | exponential_glszm_ZoneVariance |
| exponential | exponential_ngtdm_Coarseness |
| exponential | exponential_ngtdm_Complexity |
| exponential | exponential_ngtdm_Contrast |
| exponential | exponential_ngtdm_Strength |
| gradient | gradient_firstorder_10Percentile |
| gradient | gradient_firstorder_InterquartileRange |
| gradient | gradient_firstorder_Kurtosis |
| gradient | gradient_firstorder_Maximum |
| gradient | gradient_firstorder_MeanAbsoluteDeviation |
| gradient | gradient_firstorder_Median |
| gradient | gradient_firstorder_Minimum |
| gradient | gradient_firstorder_Range |
| gradient | gradient_firstorder_RobustMeanAbsoluteDeviation |
| gradient | gradient_firstorder_Skewness |
| gradient | gradient_firstorder_Uniformity |
| gradient | gradient_firstorder_Variance |
| gradient | gradient_glcm_ClusterProminence |
| gradient | gradient_glcm_ClusterShade |
| gradient | gradient_glcm_Correlation |
| gradient | gradient_glcm_Id |
| gradient | gradient_glcm_Idm |
| gradient | gradient_glcm_Idmn |
| gradient | gradient_glcm_Idn |
| gradient | gradient_glcm_Imc1 |
| gradient | gradient_glcm_Imc2 |
| gradient | gradient_glcm_InverseVariance |
| gradient | gradient_glcm_JointEnergy |
| gradient | gradient_glcm_MCC |
| gradient | gradient_glcm_MaximumProbability |
| gradient | gradient_gldm_DependenceEntropy |
| gradient | gradient_gldm_DependenceNonUniformityNormalized |
| gradient | gradient_gldm_DependenceVariance |
| gradient | gradient_gldm_LargeDependenceEmphasis |
| gradient | gradient_gldm_LargeDependenceHighGrayLevelEmphasis |
| gradient | gradient_gldm_LargeDependenceLowGrayLevelEmphasis |
| gradient | gradient_gldm_LowGrayLevelEmphasis |
| gradient | gradient_gldm_SmallDependenceHighGrayLevelEmphasis |
| gradient | gradient_gldm_SmallDependenceLowGrayLevelEmphasis |
| gradient | gradient_glrlm_GrayLevelNonUniformityNormalized |
| gradient | gradient_glrlm_GrayLevelVariance |
| gradient | gradient_glrlm_HighGrayLevelRunEmphasis |
| gradient | gradient_glrlm_LowGrayLevelRunEmphasis |
| gradient | gradient_glrlm_RunEntropy |
| gradient | gradient_glrlm_RunLengthNonUniformityNormalized |
| gradient | gradient_glrlm_ShortRunEmphasis |
| gradient | gradient_glrlm_ShortRunHighGrayLevelEmphasis |
| gradient | gradient_glrlm_ShortRunLowGrayLevelEmphasis |
| gradient | gradient_glszm_GrayLevelNonUniformity |
| gradient | gradient_glszm_GrayLevelNonUniformityNormalized |
| gradient | gradient_glszm_GrayLevelVariance |
| gradient | gradient_glszm_HighGrayLevelZoneEmphasis |
| gradient | gradient_glszm_LowGrayLevelZoneEmphasis |
| gradient | gradient_glszm_SizeZoneNonUniformityNormalized |
| gradient | gradient_glszm_SmallAreaEmphasis |
| gradient | gradient_glszm_SmallAreaHighGrayLevelEmphasis |
| gradient | gradient_glszm_SmallAreaLowGrayLevelEmphasis |
| gradient | gradient_glszm_ZoneEntropy |
| gradient | gradient_glszm_ZonePercentage |
| gradient | gradient_ngtdm_Complexity |
| gradient | gradient_ngtdm_Contrast |
| gradient | gradient_ngtdm_Strength |
| lbp-3D-m1 | lbp-3D-m1_firstorder_10Percentile |
| lbp-3D-m1 | lbp-3D-m1_firstorder_90Percentile |
| lbp-3D-m1 | lbp-3D-m1_firstorder_Entropy |
| lbp-3D-m1 | lbp-3D-m1_firstorder_InterquartileRange |
| lbp-3D-m1 | lbp-3D-m1_firstorder_Kurtosis |
| lbp-3D-m1 | lbp-3D-m1_firstorder_Maximum |
| lbp-3D-m1 | lbp-3D-m1_firstorder_MeanAbsoluteDeviation |
| lbp-3D-m1 | lbp-3D-m1_firstorder_Mean |
| lbp-3D-m1 | lbp-3D-m1_firstorder_Median |
| lbp-3D-m1 | lbp-3D-m1_firstorder_Minimum |
| lbp-3D-m1 | lbp-3D-m1_firstorder_Range |
| lbp-3D-m1 | lbp-3D-m1_firstorder_RobustMeanAbsoluteDeviation |
| lbp-3D-m1 | lbp-3D-m1_firstorder_RootMeanSquared |
| lbp-3D-m1 | lbp-3D-m1_firstorder_Skewness |
| lbp-3D-m1 | lbp-3D-m1_firstorder_Uniformity |
| lbp-3D-m1 | lbp-3D-m1_firstorder_Variance |
| lbp-3D-m1 | lbp-3D-m1_glcm_Autocorrelation |
| lbp-3D-m1 | lbp-3D-m1_glcm_ClusterProminence |
| lbp-3D-m1 | lbp-3D-m1_glcm_ClusterShade |
| lbp-3D-m1 | lbp-3D-m1_glcm_ClusterTendency |
| lbp-3D-m1 | lbp-3D-m1_glcm_Contrast |
| lbp-3D-m1 | lbp-3D-m1_glcm_Correlation |
| lbp-3D-m1 | lbp-3D-m1_glcm_DifferenceAverage |
| lbp-3D-m1 | lbp-3D-m1_glcm_DifferenceEntropy |
| lbp-3D-m1 | lbp-3D-m1_glcm_DifferenceVariance |
| lbp-3D-m1 | lbp-3D-m1_glcm_Id |
| lbp-3D-m1 | lbp-3D-m1_glcm_Idm |
| lbp-3D-m1 | lbp-3D-m1_glcm_Idmn |
| lbp-3D-m1 | lbp-3D-m1_glcm_Idn |
| lbp-3D-m1 | lbp-3D-m1_glcm_Imc1 |
| lbp-3D-m1 | lbp-3D-m1_glcm_Imc2 |
| lbp-3D-m1 | lbp-3D-m1_glcm_InverseVariance |
| lbp-3D-m1 | lbp-3D-m1_glcm_JointAverage |
| lbp-3D-m1 | lbp-3D-m1_glcm_JointEnergy |
| lbp-3D-m1 | lbp-3D-m1_glcm_JointEntropy |
| lbp-3D-m1 | lbp-3D-m1_glcm_MCC |
| lbp-3D-m1 | lbp-3D-m1_glcm_MaximumProbability |
| lbp-3D-m1 | lbp-3D-m1_glcm_SumAverage |
| lbp-3D-m1 | lbp-3D-m1_glcm_SumEntropy |
| lbp-3D-m1 | lbp-3D-m1_glcm_SumSquares |
| lbp-3D-m1 | lbp-3D-m1_gldm_DependenceEntropy |
| lbp-3D-m1 | lbp-3D-m1_gldm_DependenceNonUniformityNormalized |
| lbp-3D-m1 | lbp-3D-m1_gldm_DependenceVariance |
| lbp-3D-m1 | lbp-3D-m1_gldm_GrayLevelVariance |
| lbp-3D-m1 | lbp-3D-m1_gldm_HighGrayLevelEmphasis |
| lbp-3D-m1 | lbp-3D-m1_gldm_LargeDependenceEmphasis |
| lbp-3D-m1 | lbp-3D-m1_gldm_LargeDependenceHighGrayLevelEmphasis |
| lbp-3D-m1 | lbp-3D-m1_gldm_LargeDependenceLowGrayLevelEmphasis |
| lbp-3D-m1 | lbp-3D-m1_gldm_LowGrayLevelEmphasis |
| lbp-3D-m1 | lbp-3D-m1_gldm_SmallDependenceEmphasis |
| lbp-3D-m1 | lbp-3D-m1_gldm_SmallDependenceHighGrayLevelEmphasis |
| lbp-3D-m1 | lbp-3D-m1_gldm_SmallDependenceLowGrayLevelEmphasis |
| lbp-3D-m1 | lbp-3D-m1_glrlm_GrayLevelNonUniformityNormalized |
| lbp-3D-m1 | lbp-3D-m1_glrlm_GrayLevelVariance |
| lbp-3D-m1 | lbp-3D-m1_glrlm_HighGrayLevelRunEmphasis |
| lbp-3D-m1 | lbp-3D-m1_glrlm_LowGrayLevelRunEmphasis |
| lbp-3D-m1 | lbp-3D-m1_glrlm_RunEntropy |
| lbp-3D-m1 | lbp-3D-m1_glrlm_RunLengthNonUniformityNormalized |
| lbp-3D-m1 | lbp-3D-m1_glrlm_RunPercentage |
| lbp-3D-m1 | lbp-3D-m1_glrlm_ShortRunEmphasis |
| lbp-3D-m1 | lbp-3D-m1_glrlm_ShortRunHighGrayLevelEmphasis |
| lbp-3D-m1 | lbp-3D-m1_glrlm_ShortRunLowGrayLevelEmphasis |
| lbp-3D-m1 | lbp-3D-m1_glszm_GrayLevelNonUniformity |
| lbp-3D-m1 | lbp-3D-m1_glszm_GrayLevelNonUniformityNormalized |
| lbp-3D-m1 | lbp-3D-m1_glszm_GrayLevelVariance |
| lbp-3D-m1 | lbp-3D-m1_glszm_HighGrayLevelZoneEmphasis |
| lbp-3D-m1 | lbp-3D-m1_glszm_LowGrayLevelZoneEmphasis |
| lbp-3D-m1 | lbp-3D-m1_glszm_SizeZoneNonUniformity |
| lbp-3D-m1 | lbp-3D-m1_glszm_SizeZoneNonUniformityNormalized |
| lbp-3D-m1 | lbp-3D-m1_glszm_SmallAreaEmphasis |
| lbp-3D-m1 | lbp-3D-m1_glszm_SmallAreaHighGrayLevelEmphasis |
| lbp-3D-m1 | lbp-3D-m1_glszm_SmallAreaLowGrayLevelEmphasis |
| lbp-3D-m1 | lbp-3D-m1_glszm_ZoneEntropy |
| lbp-3D-m1 | lbp-3D-m1_glszm_ZoneVariance |
| lbp-3D-m1 | lbp-3D-m1_ngtdm_Busyness |
| lbp-3D-m1 | lbp-3D-m1_ngtdm_Coarseness |
| lbp-3D-m1 | lbp-3D-m1_ngtdm_Complexity |
| lbp-3D-m1 | lbp-3D-m1_ngtdm_Contrast |
| lbp-3D-m1 | lbp-3D-m1_ngtdm_Strength |
| lbp-3D-m2 | lbp-3D-m2_firstorder_10Percentile |
| lbp-3D-m2 | lbp-3D-m2_firstorder_90Percentile |
| lbp-3D-m2 | lbp-3D-m2_firstorder_Entropy |
| lbp-3D-m2 | lbp-3D-m2_firstorder_InterquartileRange |
| lbp-3D-m2 | lbp-3D-m2_firstorder_Kurtosis |
| lbp-3D-m2 | lbp-3D-m2_firstorder_Maximum |
| lbp-3D-m2 | lbp-3D-m2_firstorder_MeanAbsoluteDeviation |
| lbp-3D-m2 | lbp-3D-m2_firstorder_Mean |
| lbp-3D-m2 | lbp-3D-m2_firstorder_Median |
| lbp-3D-m2 | lbp-3D-m2_firstorder_Minimum |
| lbp-3D-m2 | lbp-3D-m2_firstorder_Range |
| lbp-3D-m2 | lbp-3D-m2_firstorder_RobustMeanAbsoluteDeviation |
| lbp-3D-m2 | lbp-3D-m2_firstorder_RootMeanSquared |
| lbp-3D-m2 | lbp-3D-m2_firstorder_Skewness |
| lbp-3D-m2 | lbp-3D-m2_firstorder_Uniformity |
| lbp-3D-m2 | lbp-3D-m2_firstorder_Variance |
| lbp-3D-m2 | lbp-3D-m2_glcm_Autocorrelation |
| lbp-3D-m2 | lbp-3D-m2_glcm_ClusterProminence |
| lbp-3D-m2 | lbp-3D-m2_glcm_ClusterShade |
| lbp-3D-m2 | lbp-3D-m2_glcm_ClusterTendency |
| lbp-3D-m2 | lbp-3D-m2_glcm_Contrast |
| lbp-3D-m2 | lbp-3D-m2_glcm_Correlation |
| lbp-3D-m2 | lbp-3D-m2_glcm_DifferenceAverage |
| lbp-3D-m2 | lbp-3D-m2_glcm_DifferenceEntropy |
| lbp-3D-m2 | lbp-3D-m2_glcm_DifferenceVariance |
| lbp-3D-m2 | lbp-3D-m2_glcm_Id |
| lbp-3D-m2 | lbp-3D-m2_glcm_Idm |
| lbp-3D-m2 | lbp-3D-m2_glcm_Idmn |
| lbp-3D-m2 | lbp-3D-m2_glcm_Idn |
| lbp-3D-m2 | lbp-3D-m2_glcm_Imc1 |
| lbp-3D-m2 | lbp-3D-m2_glcm_Imc2 |
| lbp-3D-m2 | lbp-3D-m2_glcm_InverseVariance |
| lbp-3D-m2 | lbp-3D-m2_glcm_JointAverage |
| lbp-3D-m2 | lbp-3D-m2_glcm_JointEnergy |
| lbp-3D-m2 | lbp-3D-m2_glcm_JointEntropy |
| lbp-3D-m2 | lbp-3D-m2_glcm_MCC |
| lbp-3D-m2 | lbp-3D-m2_glcm_MaximumProbability |
| lbp-3D-m2 | lbp-3D-m2_glcm_SumAverage |
| lbp-3D-m2 | lbp-3D-m2_glcm_SumEntropy |
| lbp-3D-m2 | lbp-3D-m2_glcm_SumSquares |
| lbp-3D-m2 | lbp-3D-m2_gldm_DependenceEntropy |
| lbp-3D-m2 | lbp-3D-m2_gldm_DependenceNonUniformityNormalized |
| lbp-3D-m2 | lbp-3D-m2_gldm_DependenceVariance |
| lbp-3D-m2 | lbp-3D-m2_gldm_GrayLevelVariance |
| lbp-3D-m2 | lbp-3D-m2_gldm_HighGrayLevelEmphasis |
| lbp-3D-m2 | lbp-3D-m2_gldm_LargeDependenceEmphasis |
| lbp-3D-m2 | lbp-3D-m2_gldm_LargeDependenceHighGrayLevelEmphasis |
| lbp-3D-m2 | lbp-3D-m2_gldm_LargeDependenceLowGrayLevelEmphasis |
| lbp-3D-m2 | lbp-3D-m2_gldm_LowGrayLevelEmphasis |
| lbp-3D-m2 | lbp-3D-m2_gldm_SmallDependenceEmphasis |
| lbp-3D-m2 | lbp-3D-m2_gldm_SmallDependenceHighGrayLevelEmphasis |
| lbp-3D-m2 | lbp-3D-m2_gldm_SmallDependenceLowGrayLevelEmphasis |
| lbp-3D-m2 | lbp-3D-m2_glrlm_GrayLevelNonUniformityNormalized |
| lbp-3D-m2 | lbp-3D-m2_glrlm_GrayLevelVariance |
| lbp-3D-m2 | lbp-3D-m2_glrlm_HighGrayLevelRunEmphasis |
| lbp-3D-m2 | lbp-3D-m2_glrlm_LowGrayLevelRunEmphasis |
| lbp-3D-m2 | lbp-3D-m2_glrlm_RunEntropy |
| lbp-3D-m2 | lbp-3D-m2_glrlm_RunLengthNonUniformityNormalized |
| lbp-3D-m2 | lbp-3D-m2_glrlm_RunPercentage |
| lbp-3D-m2 | lbp-3D-m2_glrlm_ShortRunEmphasis |
| lbp-3D-m2 | lbp-3D-m2_glrlm_ShortRunHighGrayLevelEmphasis |
| lbp-3D-m2 | lbp-3D-m2_glrlm_ShortRunLowGrayLevelEmphasis |
| lbp-3D-m2 | lbp-3D-m2_glszm_GrayLevelNonUniformity |
| lbp-3D-m2 | lbp-3D-m2_glszm_GrayLevelNonUniformityNormalized |
| lbp-3D-m2 | lbp-3D-m2_glszm_GrayLevelVariance |
| lbp-3D-m2 | lbp-3D-m2_glszm_HighGrayLevelZoneEmphasis |
| lbp-3D-m2 | lbp-3D-m2_glszm_LowGrayLevelZoneEmphasis |
| lbp-3D-m2 | lbp-3D-m2_glszm_SizeZoneNonUniformity |
| lbp-3D-m2 | lbp-3D-m2_glszm_SizeZoneNonUniformityNormalized |
| lbp-3D-m2 | lbp-3D-m2_glszm_SmallAreaEmphasis |
| lbp-3D-m2 | lbp-3D-m2_glszm_SmallAreaHighGrayLevelEmphasis |
| lbp-3D-m2 | lbp-3D-m2_glszm_SmallAreaLowGrayLevelEmphasis |
| lbp-3D-m2 | lbp-3D-m2_glszm_ZoneEntropy |
| lbp-3D-m2 | lbp-3D-m2_glszm_ZoneVariance |
| lbp-3D-m2 | lbp-3D-m2_ngtdm_Busyness |
| lbp-3D-m2 | lbp-3D-m2_ngtdm_Coarseness |
| lbp-3D-m2 | lbp-3D-m2_ngtdm_Complexity |
| lbp-3D-m2 | lbp-3D-m2_ngtdm_Contrast |
| lbp-3D-m2 | lbp-3D-m2_ngtdm_Strength |
| lbp-3D-k | lbp-3D-k_firstorder_10Percentile |
| lbp-3D-k | lbp-3D-k_firstorder_InterquartileRange |
| lbp-3D-k | lbp-3D-k_firstorder_Kurtosis |
| lbp-3D-k | lbp-3D-k_firstorder_Maximum |
| lbp-3D-k | lbp-3D-k_firstorder_MeanAbsoluteDeviation |
| lbp-3D-k | lbp-3D-k_firstorder_Median |
| lbp-3D-k | lbp-3D-k_firstorder_Minimum |
| lbp-3D-k | lbp-3D-k_firstorder_Range |
| lbp-3D-k | lbp-3D-k_firstorder_RobustMeanAbsoluteDeviation |
| lbp-3D-k | lbp-3D-k_firstorder_RootMeanSquared |
| lbp-3D-k | lbp-3D-k_firstorder_Skewness |
| lbp-3D-k | lbp-3D-k_firstorder_Uniformity |
| lbp-3D-k | lbp-3D-k_firstorder_Variance |
| lbp-3D-k | lbp-3D-k_glcm_ClusterShade |
| lbp-3D-k | lbp-3D-k_glcm_Correlation |
| lbp-3D-k | lbp-3D-k_glcm_Id |
| lbp-3D-k | lbp-3D-k_glcm_Idm |
| lbp-3D-k | lbp-3D-k_glcm_Idmn |
| lbp-3D-k | lbp-3D-k_glcm_Idn |
| lbp-3D-k | lbp-3D-k_glcm_Imc1 |
| lbp-3D-k | lbp-3D-k_glcm_Imc2 |
| lbp-3D-k | lbp-3D-k_glcm_JointEnergy |
| lbp-3D-k | lbp-3D-k_glcm_MCC |
| lbp-3D-k | lbp-3D-k_glcm_MaximumProbability |
| lbp-3D-k | lbp-3D-k_gldm_DependenceEntropy |
| lbp-3D-k | lbp-3D-k_gldm_DependenceNonUniformityNormalized |
| lbp-3D-k | lbp-3D-k_gldm_DependenceVariance |
| lbp-3D-k | lbp-3D-k_gldm_LargeDependenceEmphasis |
| lbp-3D-k | lbp-3D-k_gldm_LargeDependenceHighGrayLevelEmphasis |
| lbp-3D-k | lbp-3D-k_gldm_LargeDependenceLowGrayLevelEmphasis |
| lbp-3D-k | lbp-3D-k_gldm_LowGrayLevelEmphasis |
| lbp-3D-k | lbp-3D-k_gldm_SmallDependenceEmphasis |
| lbp-3D-k | lbp-3D-k_gldm_SmallDependenceHighGrayLevelEmphasis |
| lbp-3D-k | lbp-3D-k_gldm_SmallDependenceLowGrayLevelEmphasis |
| lbp-3D-k | lbp-3D-k_glrlm_GrayLevelNonUniformityNormalized |
| lbp-3D-k | lbp-3D-k_glrlm_GrayLevelVariance |
| lbp-3D-k | lbp-3D-k_glrlm_HighGrayLevelRunEmphasis |
| lbp-3D-k | lbp-3D-k_glrlm_LongRunEmphasis |
| lbp-3D-k | lbp-3D-k_glrlm_LongRunHighGrayLevelEmphasis |
| lbp-3D-k | lbp-3D-k_glrlm_LongRunLowGrayLevelEmphasis |
| lbp-3D-k | lbp-3D-k_glrlm_LowGrayLevelRunEmphasis |
| lbp-3D-k | lbp-3D-k_glrlm_RunEntropy |
| lbp-3D-k | lbp-3D-k_glrlm_RunLengthNonUniformityNormalized |
| lbp-3D-k | lbp-3D-k_glrlm_RunPercentage |
| lbp-3D-k | lbp-3D-k_glrlm_RunVariance |
| lbp-3D-k | lbp-3D-k_glrlm_ShortRunEmphasis |
| lbp-3D-k | lbp-3D-k_glrlm_ShortRunHighGrayLevelEmphasis |
| lbp-3D-k | lbp-3D-k_glrlm_ShortRunLowGrayLevelEmphasis |
| lbp-3D-k | lbp-3D-k_glszm_GrayLevelNonUniformityNormalized |
| lbp-3D-k | lbp-3D-k_glszm_GrayLevelVariance |
| lbp-3D-k | lbp-3D-k_glszm_HighGrayLevelZoneEmphasis |
| lbp-3D-k | lbp-3D-k_glszm_LowGrayLevelZoneEmphasis |
| lbp-3D-k | lbp-3D-k_glszm_SizeZoneNonUniformityNormalized |
| lbp-3D-k | lbp-3D-k_glszm_SmallAreaEmphasis |
| lbp-3D-k | lbp-3D-k_glszm_SmallAreaHighGrayLevelEmphasis |
| lbp-3D-k | lbp-3D-k_glszm_SmallAreaLowGrayLevelEmphasis |
| lbp-3D-k | lbp-3D-k_glszm_ZoneEntropy |
| lbp-3D-k | lbp-3D-k_glszm_ZonePercentage |
| lbp-3D-k | lbp-3D-k_ngtdm_Coarseness |
| lbp-3D-k | lbp-3D-k_ngtdm_Strength |

**Supplementary Figure S1:**

Non-small cell lung cancer tumor region of interest (ROI) segmentation from CT scans using the Dune-AI algorithm. Selected ROIs were then inspected visually using ITK-SNAP software for both case1 (on left) and case 2  (on right).


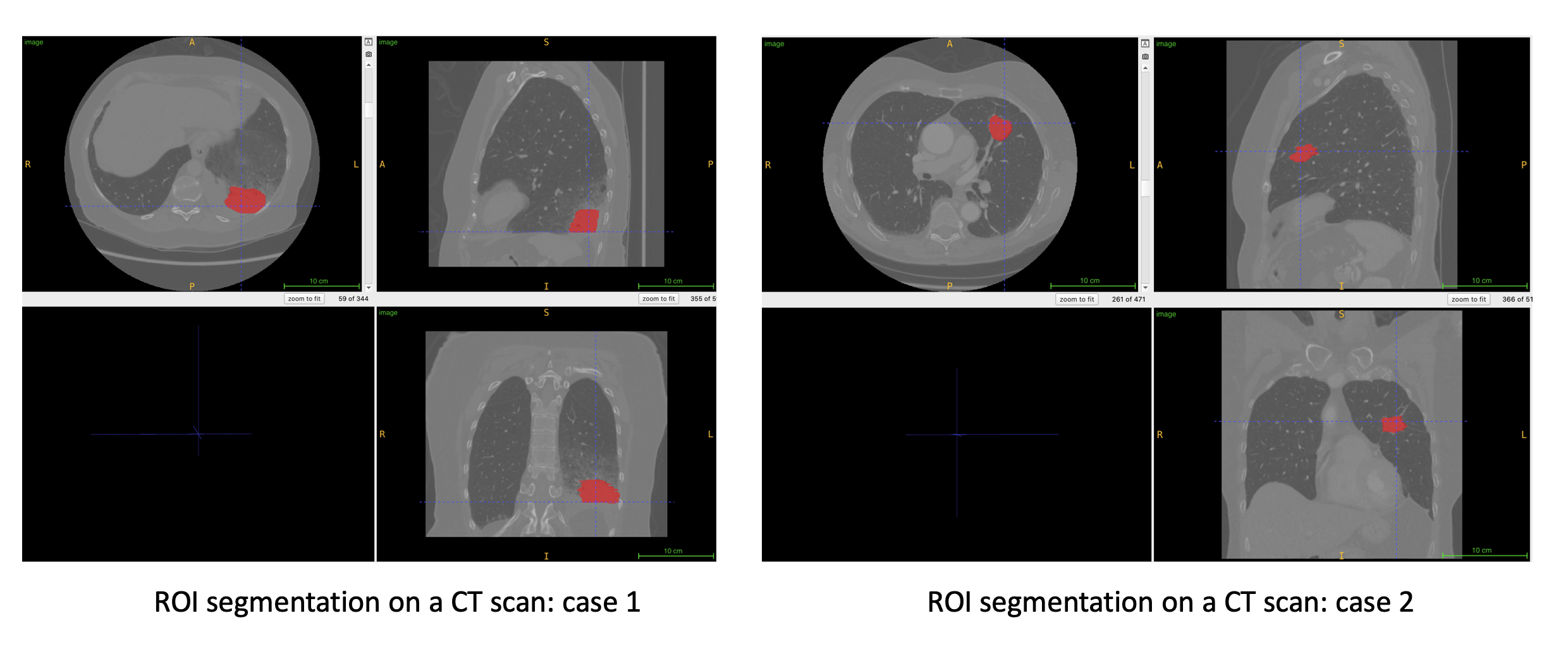


**Supplementary Figure S2:**

Co-expression scores based on RNA expression patterns, and on protein co-regulation provided by ProteomeHD. Genes co-expressing with BCL2L1 are shaded from light to dark red based on their co-expression scores.


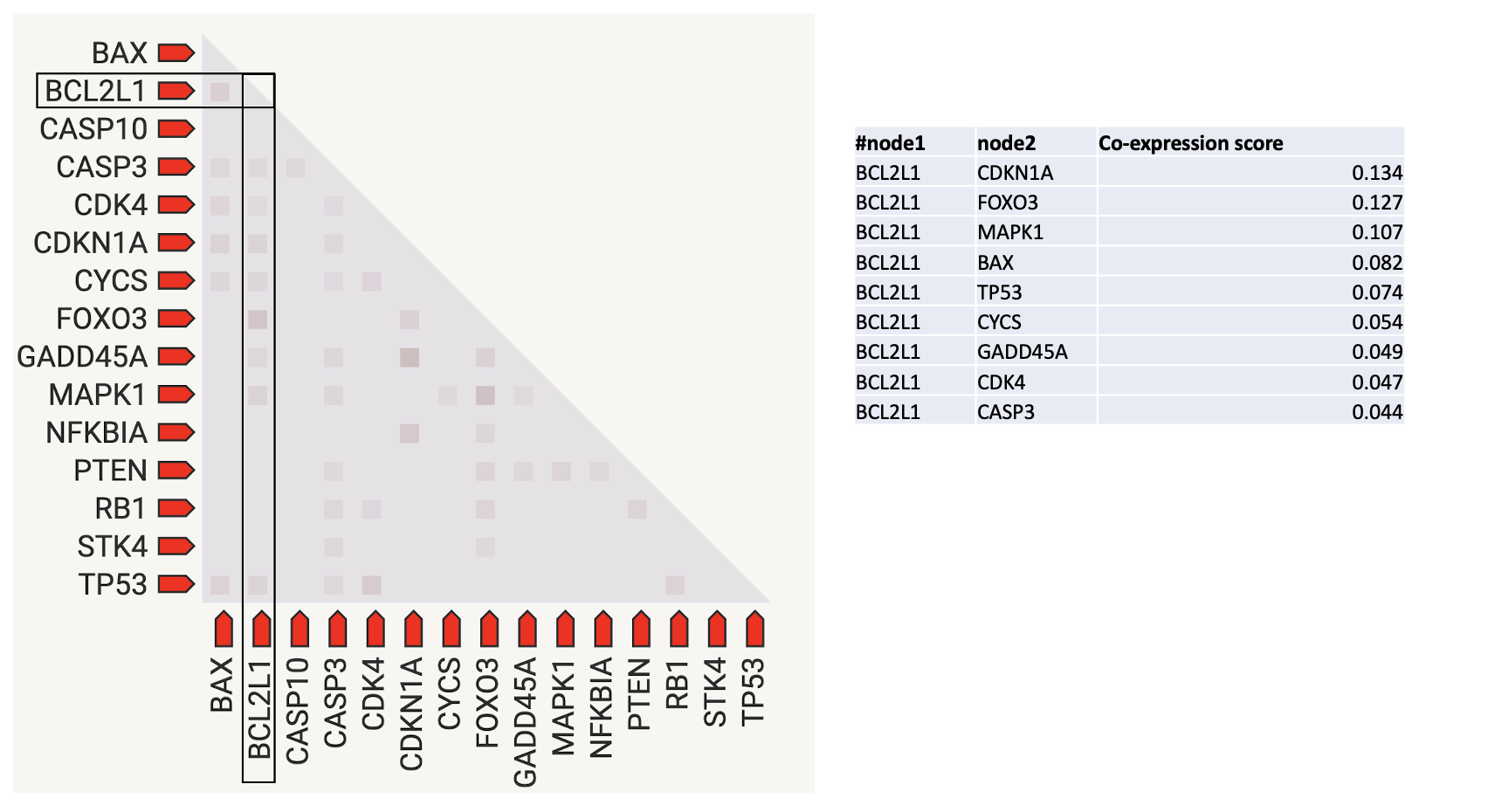


**Supplementary Figure S3:**

Interactions of BCL2L1 and MAPK1 proteins in biological pathways measured using StringDB. Green line indicates neighborhood evidence and Black line indicates co-expression evidence between the two genes.


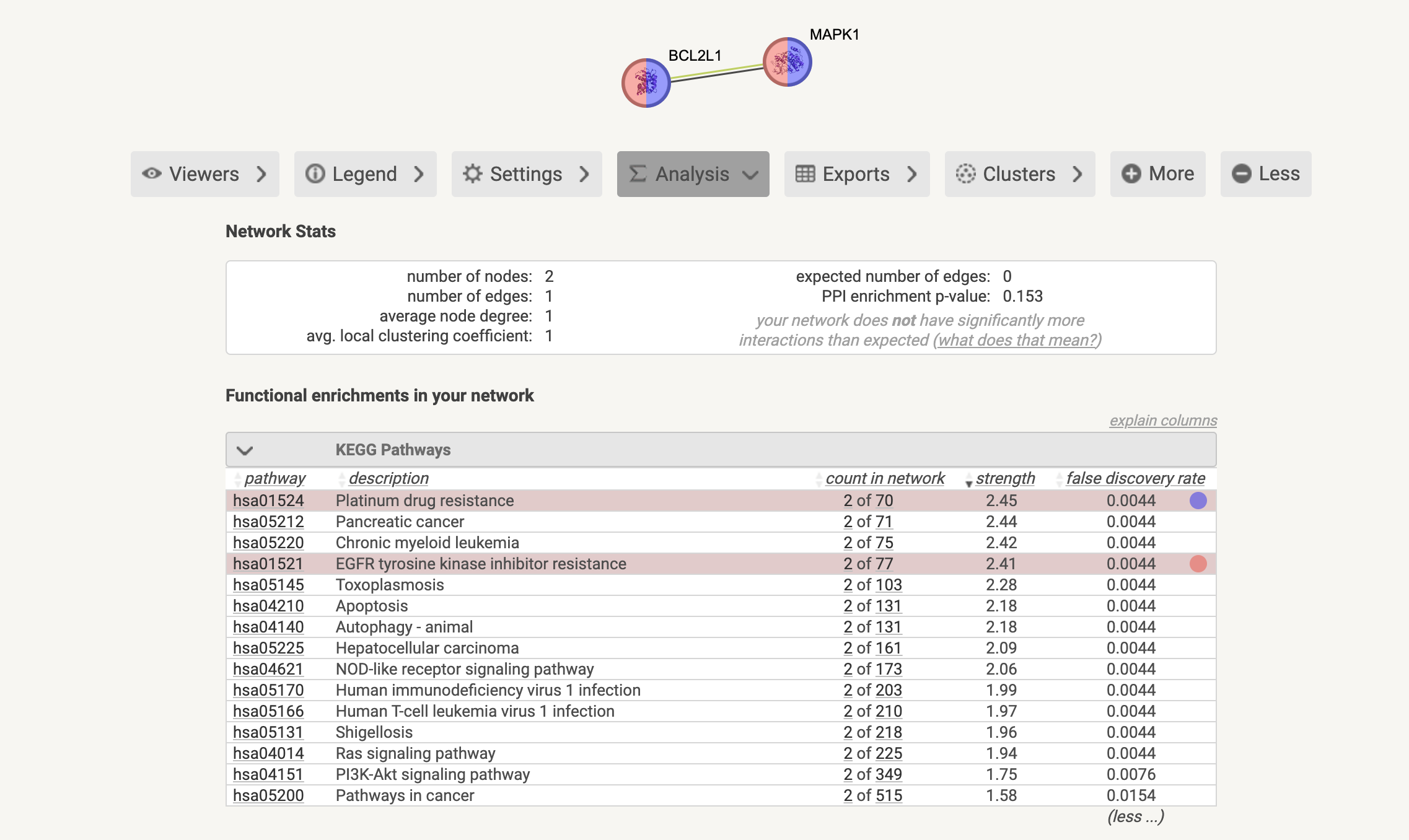

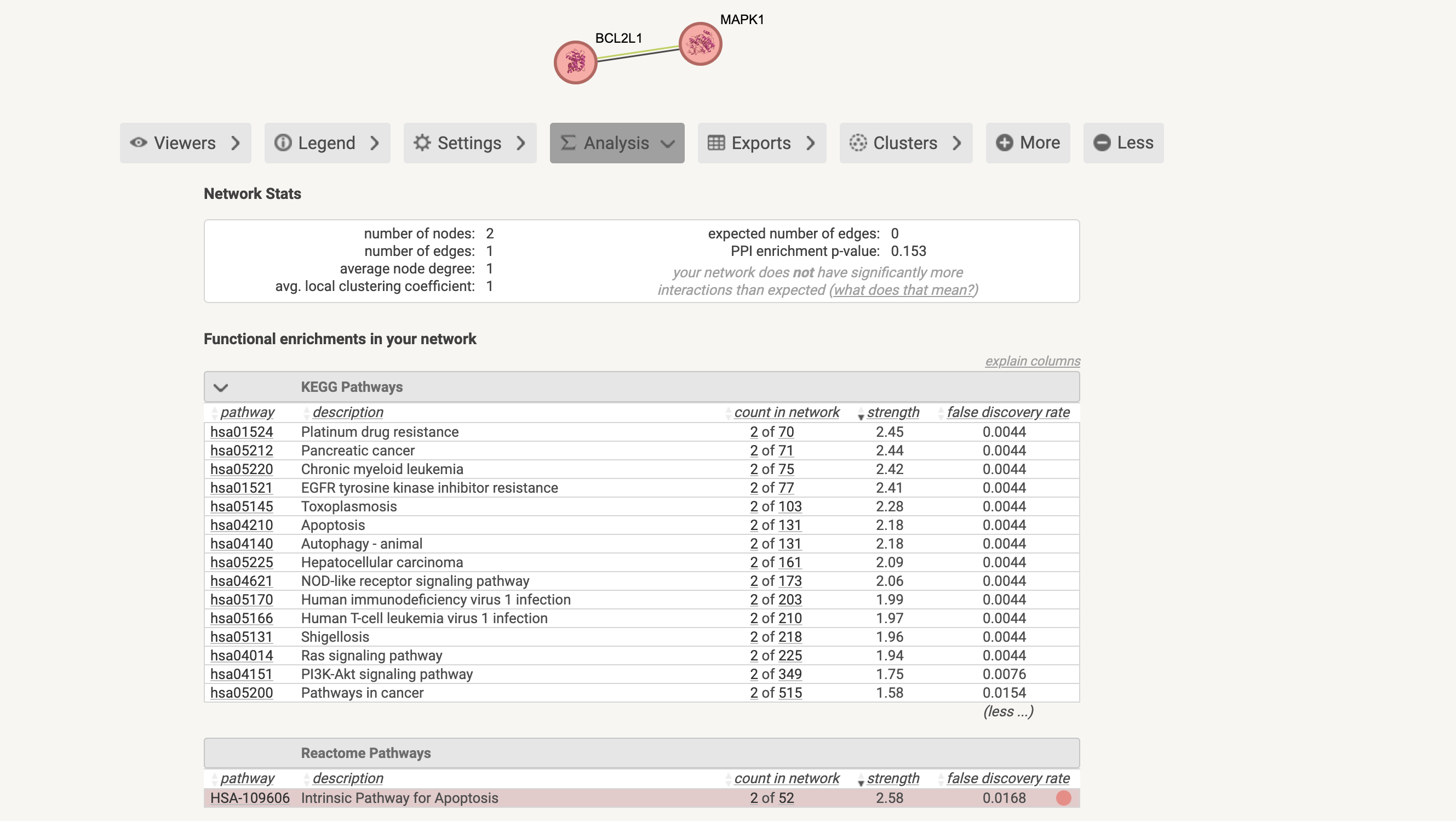

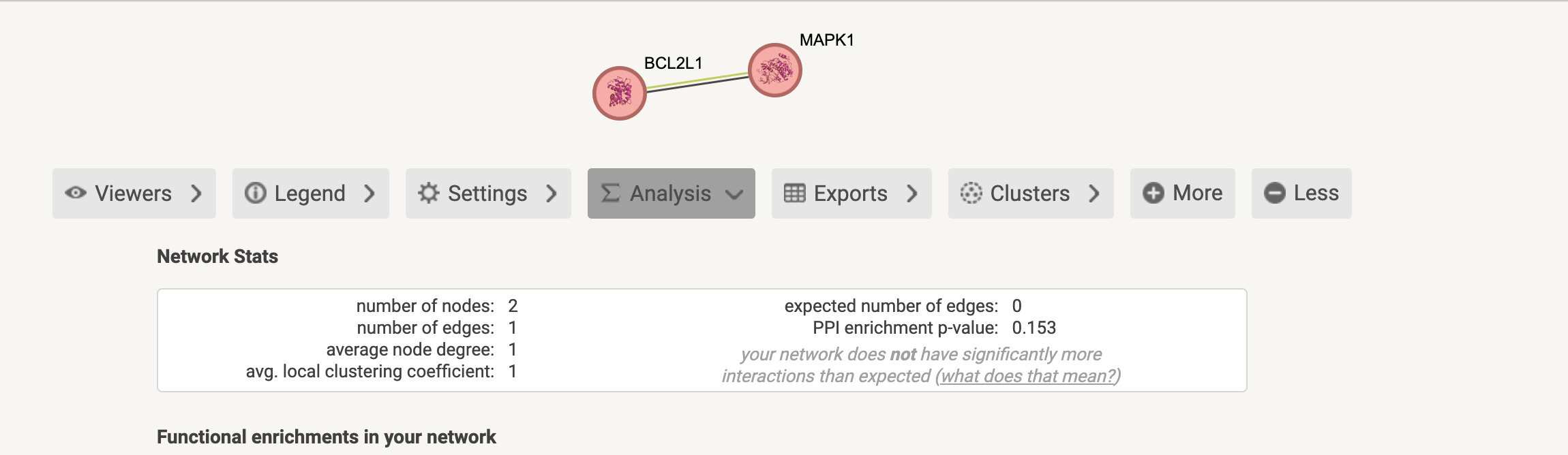

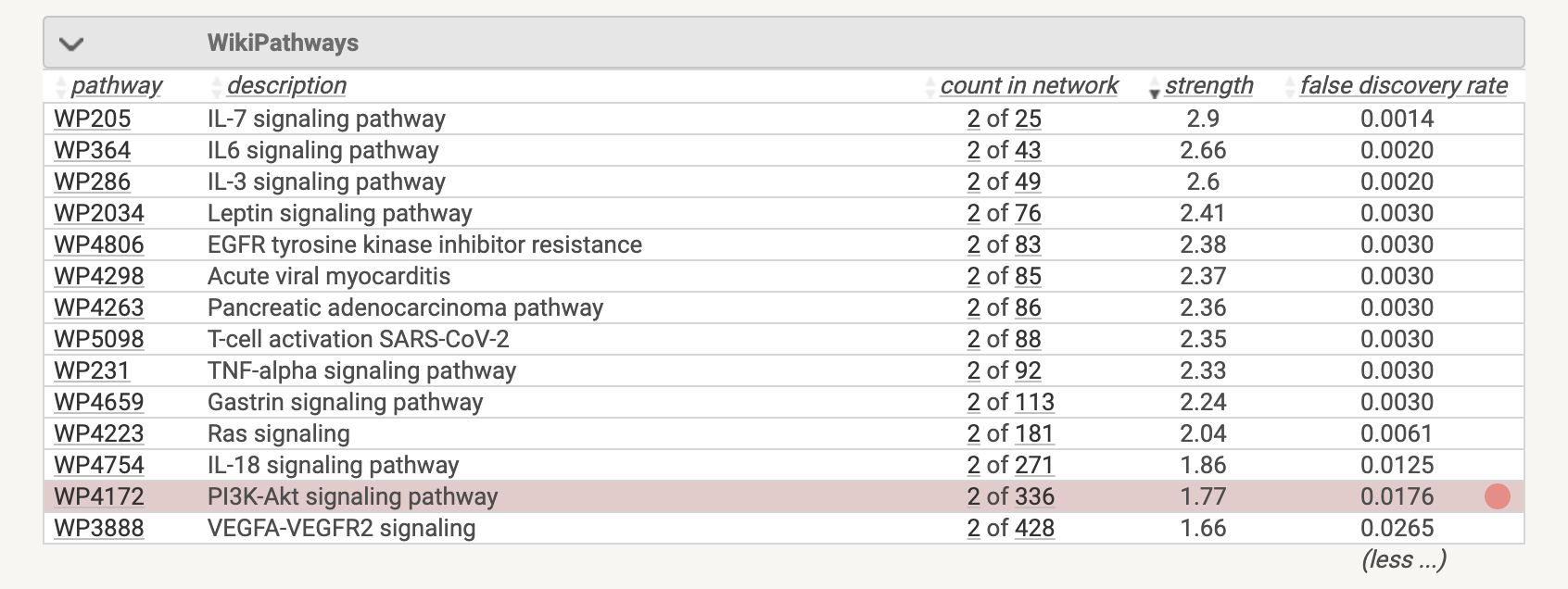

Supplement: Supplementary file 1 — Data S1. [file CAM4-13-e70509-s001.docx]
